# Biogenic Quorum-Sensing Amides from *Streptomyces* sp. NP10

**DOI:** 10.3390/molecules31010155

**Published:** 2026-01-01

**Authors:** Marija S. Genčić, Tatjana Ilic-Tomic, Marko Z. Mladenović, Milena Z. Živković Stošić, Jasmina Nikodinovic-Runic, Niko S. Radulović

**Affiliations:** 1 Department of Chemistry, Faculty of Sciences and Mathematics, University of Niš, Višegradska 33, 18000 Niš, Serbia; denijum@yahoo.com (M.S.G.); mika.zivkovic1987@gmail.com (M.Z.Ž.S.); 2 Institute of Molecular Genetics and Genetic Engineering, University of Belgrade, Vojvode Stepe 444a, 11042 Belgrade, Serbia; jasmina.nikodinovic@imgge.bg.ac.rs; 3 Department of Sciences and Mathematics, State University of Novi Pazar, Vuka Karadžića 9, 36300 Novi Pazar, Serbia

**Keywords:** *Streptomyces* sp. NP10, volatile organic compounds, biogenic amides, *Pseudomonas aeruginosa* PAO1, quorum sensing, biofilm formation, *N*-acyl homoserine lactones (AHLs), 2-alkyl-4-quinolones (AHQs), ^1^H NMR spectral simulation

## Abstract

Volatile organic compounds produced by microbes are increasingly recognized as modulators of microbial interactions and mediators of both intra- and inter-kingdom communication. This study explored the possible ecophysiological roles of nine amides from *Streptomyces* sp. NP10 in quorum sensing (QS) and biofilm formation in *Pseudomonas aeruginosa* PAO1. GC-MS profiling, synthesis, spectral validation, and co-injection experiments confirmed compound identities. Notably, *N*-(3-methyl-2-butenyl)acetamide is reported as a new natural product and *N*-(2-methylbutyl)acetamide as a new *Streptomyces*-produced metabolite. At subinhibitory concentrations (250 μg/mL), most of the amides enhanced *P. aeruginosa* biofilm formation, with *N*-(2-methylbutyl)acetamide, *N*-(3-methyl-2-butenyl)acetamide, and 2-phenylacetamide showing the strongest effects. Simultaneously, these compounds suppressed QS by reducing the production of *N*-acyl homoserine lactones (AHLs) and 2-alkyl-4-quinolones (AHQs). Aliphatic acetamides preferentially inhibited short-chain AHLs, while *N*-acetyltyramine and 2-phenylacetamide mainly affected quinolone signaling. These opposing effects on QS and biofilm are consistent with the involvement of alternative regulatory circuits. Motility assays showed biofilm stimulation was not correlated with altered swarming or twitching. Cross-species assays revealed limited QS inhibition, with only *N*-acetyltryptamine reducing violacein production in *Chromobacterium violaceum* CV026. Most of the amides were non-cytotoxic at 100 μM (10.5–20.2 μg/mL), except for 2-phenylacetamide. Overall, these amides likely serve as microbial signals influencing QS and biofilm formation, offering leads for anti-virulence strategies.

## 1. Introduction

Streptomycetes are a large group of mostly soil filamentous bacteria, characterized by a complex lifecycle, and they are spread across different environments of the world. They are best recognized for their remarkable ability to produce a wide array of secondary metabolites. This genus is responsible for nearly half of the antibiotics used in modern medicine, along with other therapeutically valuable compounds such as anticancer agents, antifungals, immunosuppressants, and biocontrol molecules [1,2]. While these secondary metabolites are not crucial for the growth of *Streptomyces*, they play important roles in helping these bacteria adapt to their surroundings. For instance, many of these compounds exhibit antimicrobial properties, enabling *Streptomyces* to inhibit rival microorganisms, thus securing nutrients and improving their chances of survival and reproduction in the soil ecosystem. This competitive advantage underscores the ecological significance of *Streptomyces* and their diverse metabolic capabilities [3]. Additionally, *Streptomyces* species produce enzymes with significant biotechnological potential [4], serve as non-conventional hosts for recombinant protein production [5], and have demonstrated biodegradation capabilities, e.g., our recent study showed that the NP10 strain (the strain was taxonomically identified by multi-locus sequence typing as *Streptomyces rubiginosohelvolus*) accelerated cotton textile decomposition by 14% in composting tests [6].

Quorum sensing (QS) is a form of cell-to-cell communication that allows bacteria, and sometimes fungi, to regulate collective behaviors through signaling molecules. In Gram-negative bacteria such as *Pseudomonas* species, QS typically relies on signaling molecules called autoinducers, particularly *N*-acyl homoserine lactones (AHLs), which are synthesized by LuxI-type enzymes and detected by LuxR-type receptors. Once a threshold concentration is reached, these signals trigger coordinated expression of genes linked to survival and competitiveness, including biofilm formation, spore production, antibiotic biosynthesis, bioluminescence, and plasmid transfer [7]. Disruption of this system, termed quorum quenching, can occur through inhibition of signal production, degradation of signaling molecules, or interference with receptor binding. Quorum-sensing inhibitors (QSIs), which interfere with these pathways without applying strong selective pressure, have promising applications in medicine, agriculture, and food preservation [8].

Recent findings by Zhan et al. [9] have highlighted the key role of AHLs in regulating spoilage-associated phenotypes in *Pseudomonas fluorescens*, such as biofilm formation, exopolysaccharide production, motility, and siderophore biosynthesis. Moreover, AHLs contribute to biogenic amine (BA) accumulation by modulating extracellular protease activity and amino acid metabolism. These results advance understanding of the molecular mechanisms driving BAs formation and spoilage in aquatic products, and position AHLs as pivotal targets for innovative spoilage control strategies.

*Streptomyces* strains with QS inhibitory activity, and the search for new QSIs is a promising strategy that opens up a new perspective for controlling QS-mediated bacterial pathogens [10]. In our previous study, we discovered that *Streptomyces* sp. NP10 (ISS613), an environmental isolate, produces and releases significant quantities of free long-chain fatty acids into its growth medium [11,12]. A lipidomics analysis revealed a wide structural diversity of fatty acids, with over 50 different types, including *n*- and branched chains, saturated and unsaturated, as well as cyclopropane fatty acids (C_7_–C_30_) produced by this strain. Although these free fatty acids exhibited only moderate antimicrobial activity, our findings suggest they may play an ecophysiological role in interspecies signaling with the soil microorganism *P. aeruginosa*.

As part of our ongoing search for novel, structurally diverse, and potentially bioactive metabolites from *Streptomyces*, we have now focused on the production of small volatile organic compounds (VOCs) by the *Streptomyces* sp. NP10 strain because VOCs emitted by microorganisms may play a significant role in microbial interactions. For instance, they can inhibit or stimulate the growth of microorganisms and plants, induce systemic resistance in plants, and affect various organisms like insects and nematodes [13]. Moreover, VOCs are crucial for microbial competition in ecological niches such as the rhizosphere and soil, and they influence relationships between plant-pathogenic and beneficial bacteria, as well as microorganisms in human and animal microbiota. Additionally, they may act as infochemicals, facilitating communication between organisms over long distances [14].

**Figure 1:**
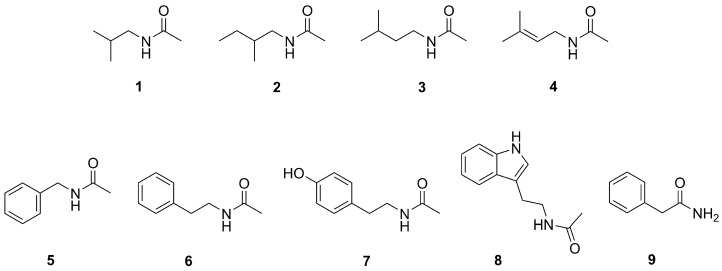
Structures of volatile amides **1**–**9** produced by *Streptomyces* sp. NP10.

In this work, VOCs are considered to be those compounds that are volatile under the conditions of the GC-MS analysis. Accordingly, we conducted a detailed GC-MS analysis of the ethyl acetate extract from the whole culture of the strain *Streptomyces* sp. NP10 to investigate its repertoire of volatile metabolites. The MS data revealed nine volatile amides (**1**–**9**; Figure 1), including one new natural product and one identified for the first time as a metabolite of *Streptomyces*. Due to their low abundance in the extract, we synthesized authentic standards and confirmed their identities by spectral analysis and co-injection experiments. Most of the amides were acetylated forms of common BAs, such as isobutylamine, isopentylamine, phenethylamine, tyramine, and tryptamine. Among these, *N*-acetyltyramine (**7**) and its precursor tyramine are known QS modulators produced by the marine bacterium *Vibrio alginolyticus* [15]. These molecules have been shown to inhibit violacein production in *Chromobacterium violaceum* ATCC 12472, as well as suppress virulence-associated phenotypes in *P. aeruginosa* PAO1, including pyoverdine production and motility. Our synthetic approach provided access to sufficient quantities of these metabolites, allowing us to explore their potential (eco)physiological roles, including cytotoxicity toward eukaryotic cells, antimicrobial properties, and QS modulation across diverse bacterial (microbial) species. This study aimed to expand our understanding of the chemical ecology of *Streptomyces* and highlight the potential of minor metabolites as modulators of microbial behavior.

## 2. Results and Discussion

### 2.1. Identification and Synthesis of Amides ***1***–***9***

GC–MS analysis of the ethyl acetate extract from *Streptomyces* sp. NP10 revealed four branched-chain aliphatic acetamides eluting within the first 10 min of the GC run. These were preliminarily identified as *N*-(2-methylpropyl)acetamide (**1**), *N*-(2-methylbutyl)acetamide (**2**), *N*-(3-methylbutyl)acetamide (**3**), and *N*-(3-methyl-2-butenyl)acetamide (**4**) (Figure 1) based on a combination of data coming from their mass spectra and/or gas chromatographic retention behavior. In the mass spectra of acetamides **1** and **2**, an abundant acetyl ion fragment was observed at *m*/*z* 43, along with characteristic ions at *m*/*z* 72 and *m*/*z* 100, which resulted from bond cleavage adjacent to the tertiary carbon atom [16,17]. Acetamide **2** eluted slightly faster than its regioisomer **3** (ΔRI = 7), and these two could also be distinguished by their differing mass fragmentation patterns. For acetamide **3**, cleavage at adjacent bonds to the tertiary carbon produced ions [M–CH_3_] at *m*/*z* 114 and [M–C_3_H_7_] at *m*/*z* 86 [17]. These three aliphatic acetamides **1**–**3** were only occasionally reported from bacteria [18,19,20,21,22]; compounds **1** and **3** were each detected only once as volatiles from *Streptomyces antimycoticus* [20], whereas compound **2** has not been found in any *Streptomyces* species to date. The parent ion of compound **4** at *m*/*z* 127 indicated the molecular formula C_7_H_13_NO, suggesting it is the acetamide of a monounsaturated amine with five carbon atoms. The unusual base peak at *m*/*z* 70 and fragment ions at *m*/*z* 84 and 122, corresponding to [M–CH_3_CO]^+^ and [M–CH_3_]^+^, respectively, implied that this metabolite was likely *N*-(3-methyl-2-butenyl)acetamide [23], a compound that has not been previously reported as a natural product. The linear retention index (RI) value of acetamides **1**, **2,** and **3** aligned with previously published data [17,18,24], whereas retention data for acetamide **4** were not found in the literature.

A structure-based literature and database search was conducted using SciFinder (CAS) on 13 November 2025. Both exact structure and substructure queries were performed for all identified amides, including *N*-(3-methyl-2-butenyl)acetamide. No records were found indicating its prior isolation as a natural product, supporting its annotation as a new natural product. Similarly, another aliphatic acetamide, *N*-(2-methylbutyl)acetamide, had no previous reports from *Streptomyces*, suggesting it represents a new *Streptomyces*-derived metabolite.

The identity of *N*-benzylacetamide (**5**) was also proposed based on positive matches of its mass spectrum and RI value with those reported in the literature [25]. Notably, this compound has only been found in *Streptomyces* on one previous occasion, having been isolated from the culture broth of the marine *Streptomyces* sp. isolate B6921 [26]. Three additional acetamides, **6**–**8**, originated from BAs phenethylamine, tyramine, and tryptamine (which are derived from the amino acids phenylalanine, tyrosine, and tryptophan, respectively), and one primary amide **9** (derived from phenylacetic acid) were identified by a straightforward (EI-MS) database comparison procedure. These amides are regarded as more common metabolites in *Streptomyces* and have been shown to exhibit antimicrobial and antioxidant effects [27,28,29,30,31,32,33].

**Figure 2:**
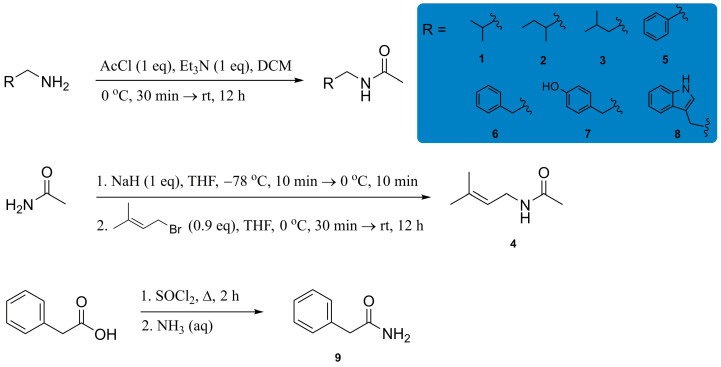
Synthetic routes to amides **1**–**9**.

Synthetic standards of acetamides **1**–**3** and **5**–**8** were prepared via the Schotten-Baumann reaction by treating the corresponding commercially available amines with acetyl chloride (Figure 2). Acetamide **4** was synthesized via the substitution of prenyl bromide with a nitrogen-centered nucleophile, formed upon deprotonation of acetamide with NaH. Amide **9** was synthesized via the conversion of 2-phenylacetic acid to the corresponding acyl chloride, followed by nucleophilic substitution with concentrated aqueous ammonia. Reported yields are isolated yields after chromatographic purification. Reactions were performed to secure material for full spectroscopic characterization and bioassays; conditions were not optimized, and potential side products were not pursued. All synthetic standards were fully spectrally characterized (MS, IR, and NMR; Figures S1–S36; Table S1) and their spectral data were in general agreement with previously published data [16,17,23,34,35,36,37,38,39]. Gas co-chromatography of the obtained amides **1**–**9** with the whole culture ethyl acetate extract of *Streptomyces* sp. NP10 unambiguously confirmed the original assumption of their presence in the extract (Figures S37–S39). To contextualize their prevalence, all were below 0.05% of the detected GC–peak areas, i.e., were present only in trace amounts.

It seems that although ^1^H NMR data for amides **1**–**9** are available in the literature, they are often incomplete or inaccurate, particularly regarding signal multiplicities and the corresponding coupling constant values. This prompted us to perform simulations of the ^1^H NMR spectra for amides **1**–**9** using manual iterative full spin analysis (Figures S40–S48). Comprehensive analyses and discussion of these results are included in the Supplementary Materials [17,23,35,37,38,40,41,42,43,44,45,46,47,48,49,50].

### 2.2. Modulation of QS Systems in Pseudomonas aeruginosa by Amides ***1***–***9***

PAO1 is a well-characterized strain of *Pseudomonas aeruginosa*, a Gram-negative opportunistic pathogen associated with biofilm-related infections in cystic fibrosis, burn wounds, indwelling medical devices, and nosocomial settings [51,52]. This strain is widely recognized as a model organism for elucidating the molecular mechanisms of QS and for studying the ecological and evolutionary basis of collective behaviors in bacterial communities [53]. Many of its virulence traits, including motility and pyocyanin, rhamnolipid, and protease production, are tightly regulated by interconnected QS systems in a cell density-dependent manner, with peak expression typically occurring during the late logarithmic to early stationary growth phases. QS also plays a key role in biofilm formation, particularly in regulating the development and structural integrity of the biofilm matrix. These QS-regulated behaviors contribute significantly to *P. aeruginosa*’s resistance to multiple antibiotics, complicating treatment and posing a major clinical challenge [10].

To date, three major QS systems have been identified in *P. aeruginosa*: the LasI/LasR system, producing *N*-3-oxo-dodecanoyl-homoserine lactone (3-oxo-C12-HSL); the RhlI/RhlR system, producing *N*-butanoyl-homoserine lactone (C4-HSL); and the Pqs system, which uses 2-alkyl-4-quinolones (AHQs) regulated by the transcription factor PqsR. While LasR and RhlR primarily respond to changes in cell population density, the Pqs system is more closely associated with environmental stress responses [54]. Beyond biofilm regulation, the AHL systems govern the expression of other key virulence factors, including LasA protease, phospholipase, exotoxin A, pyocyanin, rhamnolipids, and elastase. Similarly, AHQ signaling controls many of these genes and additionally regulates those involved in iron scavenging [53].

Pyocyanin is a bioactive, redox-active secondary metabolite produced by *P. aeruginosa*, functioning as both a key virulence factor and QS signaling molecule involved in the pathogenesis of infection [55]. In the reference strain PAO1, pyocyanin biosynthesis is regulated primarily through AHL-mediated QS pathways [52]. In our previous study, we found that the crude extract of *Streptomyces* sp. NP10 moderately reduced pyocyanin production in *P. aeruginosa* PAO1 by approximately 20% at a concentration of 100 μg/mL, without affecting bacterial growth [11]. Moreover, one of the identified amides, *N*-acetyltyramine (**7**), has been reported to possess QS inhibitory activity. Specifically, it significantly reduced violacein production in *Chromobacterium violaceum* ATCC 12472, as well as key virulence factors in *P. aeruginosa* PAO1, including pyoverdine production, swarming, and twitching motilities [15]. These findings motivated us to further investigate the potential (eco)physiological roles of volatile amides **1**–**9** by assessing their impact on the QS system of *P. aeruginosa* PAO1 through multiple assays measuring pyocyanin, AHLs, and AHQs production, biofilm formation, motility behaviors, and interactions with bacterial DNA.

#### 2.2.1. Impact of Amides on Pyocyanin Production

Firstly, the antimicrobial activity of amides **1**–**9** against *P. aeruginosa* PAO1 was evaluated using a broth microdilution assay. Several compounds (**1**, **4**, **6**, **7**, and **9**) inhibited the growth of planktonic *P. aeruginosa* PAO1 cells, but only at the highest tested concentration (500 μg/mL; ~50% of inhibition). Based on these findings, and considering the physicochemical properties of amides **1**–**9**, such as their low molecular weight, high volatility, and presumed susceptibility to environmental degradation (e.g., enzymatic breakdown by proteases), they were further assessed for their ability to modulate bacterial cell-to-cell communication in *P. aeruginosa* PAO1. However, no apparent reduction in the characteristic green pigmentation associated with pyocyanin production was observed following treatment with any of the amides at 250 μg/mL, suggesting a lack of significant effect on pyocyanin biosynthesis at subinhibitory concentrations (Figure S49). Previously, the effects of *N*-acetyltyramine (**7**) and its precursor BA, tyramine, on the production of pyoverdine, a fluorescent siderophore critical for iron acquisition and virulence in *P. aeruginosa*, were investigated [15]. Both compounds inhibited pyoverdine production, but only at substantially higher concentrations (1–2 mg/mL) than those tested in our study, with tyramine showing considerably greater activity than its acetylated form. Likewise, the structurally related tyramine derivative hordenine (*N*,*N*-dimethyltyramine) was shown to reduce pyocyanin biosynthesis in *P. aeruginosa* PAO1 by 40% and 80%, but again, only at the much higher concentration of 500 and 1000 μg/mL, respectively [56].

#### 2.2.2. Amide-Induced Enhancement of Biofilm Formation

**Figure 3:**
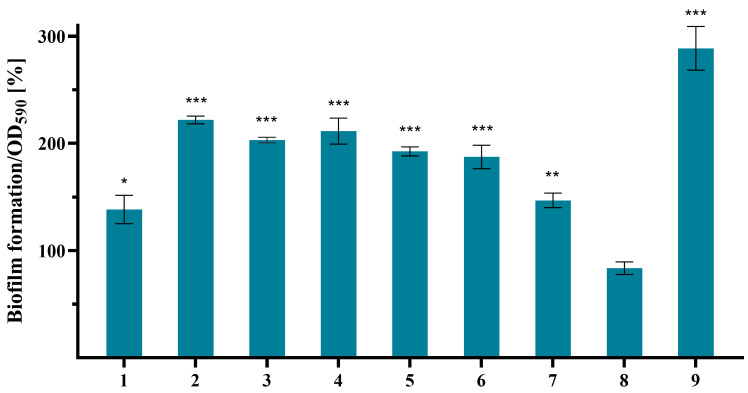
Effect of amides **1**–**9** on biofilm production by *P. aeruginosa* PAO1. Data are presented as mean ± SD (*n* = 2 × 5). Statistical analysis was performed using one-way ANOVA followed by Dunnett’s multiple comparisons test (vs. control). *** *p* < 0.001, ** *p* < 0.01, and * *p* < 0.05 indicate statistically significant differences compared to the control.

Subsequently, the effect of amides **1**–**9** on biofilm formation by *P. aeruginosa* PAO1 was evaluated at a concentration of 250 μg/mL after 24 h of incubation. The results showed that all tested compounds, except *N*-acetyltryptamine (**8**), promoted biofilm formation to varying degrees (Figure 3). The most pronounced stimulatory effects were observed with *N*-(2-methylbutyl)acetamide (**2**), *N*-(3-methyl-2-butenyl)acetamide (**4**), and 2-phenylacetamide (**9**), which induced a 2- to 3-fold increase in biofilm biomass compared to the DMSO control.

In contrast, *N*-acetyltryptamine (**8**) slightly reduced biofilm formation (16%), consistent with previous findings on its parent BA, tryptophan, which caused approximately 30% reduction in biofilm biomass in various *P. aeruginosa* strains at 150 μg/mL [57] and 50 μg/mL [58] after 24 and 48 h of incubation, respectively. This suggests that the free primary amino group may be essential for stronger antibiofilm activity. Supporting this, the plant-derived tyramine derivative hordenine, bearing a free tertiary amino group, showed a similar effect at a concentration of 750 μg/mL [56].

#### 2.2.3. Influence of Amides on Autoinducers Production

**Figure 4:**
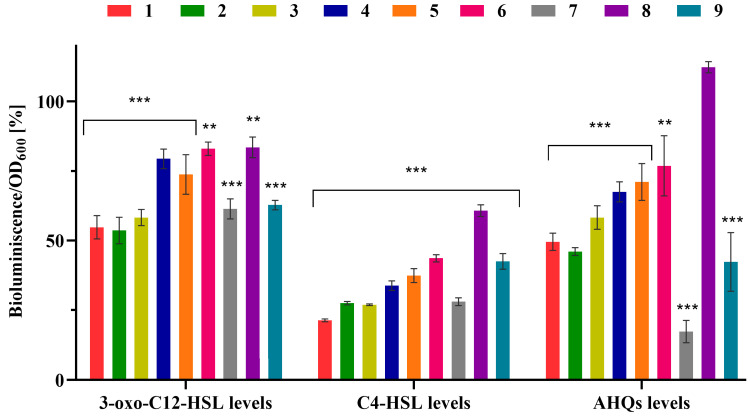
Effect of amides **1**–**9** on acyl homoserine lactone (AHL) and 2-alkyl-4-quinolone (AHQ) production in *P. aeruginosa* at 250 µg/mL. Specific reporter strains were used to assess signal molecule production: PA14-R3ΔlasI *PrsaI:lux* for detection of 3-oxo-C12-HSL, PAO1ΔrhlI *pKD-rhlA* for detection of C4-HSL, and PAO1ΔpqsA *mini-CTX luxPpqsA* for monitoring AHQ levels. Data are presented as mean ± SD (*n* = 3 × 3). Statistical analysis was performed using one-way ANOVA followed by Dunnett’s multiple comparisons test (vs. control). ** *p* < 0.01; *** *p* < 0.001 indicate statistically significant differences compared to control.

To evaluate the potential of amides **1**–**9** to modulate AHL production in *P. aeruginosa*, cultures were treated with each compound at concentrations of 50 or 250 µg/mL. To specifically assess their effects on the Las and Rhl QS systems, two biosensor strains were employed: *P. aeruginosa* PA14-R3Δ*lasI* *PrsaI*::*lux*, which detects long-chain AHLs (3-oxo-C12-HSL), and PAO1 ΔrhlI*pKD*-*rhlA*, which is responsive to short-chain AHLs (C4-HSL). At the higher tested concentration, all amides demonstrated a more pronounced inhibition of short-chain AHL production (C4-HSL; 39–78%) compared to long-chain AHLs (3-oxo-C12-HSL; 16–45%). Aliphatic acetamides, particularly compounds **1**–**3**, as well as *N*-acetyltyramine (**7**), were the most effective in suppressing AHL biosynthesis (Figure 4). At the lower concentration, these inhibitory effects were significantly reduced. Interestingly, at this concentration, *N*-(2-phenylethyl)acetamide (**6**) slightly stimulated the production of both AHLs, with an increase of approximately 30%.

To evaluate the impact of amides **1**–**9** on AHQ production in *P. aeruginosa*, biosensor strain *P. aeruginosa* PAO1 ΔpqsA mini-CTX luxPpqsA was cultured in the presence of each compound at the same two concentrations. At the higher tested concentration (250 μg/mL), with the exception of *N*-acetyltryptamine (**8**), all other tested amides reduced the release of AHQ molecules (Figure 4). The most pronounced inhibition was observed with *N*-acetyltyramine (**7**; 83%), followed by 2-phenylacetamide (**9**; 58%), *N*-(2-methylbutyl)acetamide (**2**; 54%), and *N*-(2-methylpropyl)acetamide (**1**; 50%). Notably, at the lower concentration (50 μg/mL), only amide **9** exhibited a statistically significant suppression of AHQ production in *P. aeruginosa*, with an effect comparable in magnitude to that observed at the higher tested concentration (52% vs. 58%).

Our findings highlight the concentration-dependent inhibition of both AHL and AHQ production by most tested amides, with *N*-acetyltyramine (**7**) exhibiting the strongest overall activity. A structurally related tyramine derivative, hordenine, also demonstrated selective inhibition of short-chain AHLs (69%) over long-chain AHLs (24%), though this effect required a two-fold higher concentration (500 μg/mL) [56], indicating a comparatively lower potency. Notably, amide **9** consistently reduced AHQs production at both concentrations tested and was the only compound to significantly inhibit AHQ levels at the lower dose, underscoring its potential as a robust modulator of quinolone signaling. In contrast, the limited activity of *N*-(2-methylpropyl)acetamide (**1**) at 50 μg/mL is consistent with previous findings showing no significant impact on the expression of *pqsA*, the gene encoding a key enzyme in the biosynthesis of 2-heptyl-3-hydroxy-4-quinolone (PQS), a major quinolone signal molecule in *P. aeruginosa* [34].

Amides **7** and **9**, in particular, appear to modulate the quinolone-mediated QS system in *P. aeruginosa* more effectively than the other tested compounds. Given that many QS signals, such as AHQs and long-chain AHLs, are highly hydrophobic and often transported via specialized mechanisms like membrane vesicles [59], the structural features of **7** and **9**, including their (substituted)phenyl rings, may enhance their interaction with QS components or their integration into these transport systems. This structural similarity to native signaling molecules may account for their enhanced ability to interfere with QS-regulated pathways.

Overall, the results indicated that the tested amides are capable of modulating both AHL- and AHQ-dependent QS systems in *P. aeruginosa* PAO1. Most of the compounds inhibited the production of both AHLs and AHQs, suggesting that their observed stimulatory effect on biofilm formation is consistent with modulation of regulatory pathways beyond classical AHL/AHQ circuits, such as cyclic di-GMP signaling. Given that *Streptomyces* is among the most widespread bacterial genera in natural environments, it is plausible that other microbes, including *P. aeruginosa*, have evolved the ability to detect and respond to VOCs released by *Streptomyces* species, such as amides **1**–**7** and **9**. This interspecies chemical sensing may represent an adaptive survival strategy, wherein biofilm formation is enhanced in response to environmental cues interpreted as signals of microbial competition or stress.

*N*-acetyltryptamine (**8**), the only compound that slightly reduced biofilm formation, also exhibited the weakest inhibition of AHLs production while paradoxically stimulating AHQs biosynthesis. Previous studies have demonstrated that tryptophan, a metabolic precursor of **8**, can suppress biofilm development in *P. aeruginosa* and downregulate key QS-related genes such as *lasR*, *lasB*, and *lasI* [58]. The degradation of tryptophan varies among bacterial species, yielding metabolites such as indole and anthranilate. In *P. aeruginosa*, indole has been reported to enhance biofilm formation, whereas anthranilate, the main catabolite of tryptophan in this species, inhibits it [60]. Therefore, the modest reduction in biofilm observed with compound **8** may be attributed not to the parent molecule itself, but to one of its metabolic degradation products—potentially anthranilate. This observation offers a plausible explanation for the divergent effects exhibited by the other tested amides, which simultaneously stimulated biofilm formation while inhibiting QS autoinducer production, and highlights the potential influence of intracellular metabolic processing on the modulation of QS-associated phenotypes.

#### 2.2.4. Interaction of Amides with Bacterial DNA

Extracellular DNA (eDNA) is a major component of the *P. aeruginosa* biofilm matrix, and its abundance varies depending on biofilm maturity and environmental conditions. The eDNA of the matrix is generated via QS-dependent and QS-independent pathways. The one mediated by QS occurs via the lysis of a small population of cells, while the other liberates only basal levels of eDNA. There are other ways to promote *P. aeruginosa* death and the production of eDNA, such as the intracellular increase in H_2_O_2_ due to pyocyanin exposure [54]. Thus, eDNA represents a promising target for biofilm control, either through enzymatic degradation, direct intercalation-based complex formation, or disruption of its interactions with other matrix components such as proteins or polysaccharides [61]. Therefore, to investigate the potential of amides **1**–**9** to interact with bacterial DNA, their ability to competitively intercalate into double-stranded genomic DNA (gDNA) was evaluated. In the method used, DNA intercalation is assessed based on the ability of test compounds to displace ethidium bromide, leading to reduced fluorescence under UV light, which is visualized by agarose gel electrophoresis. However, none of the amides tested at a concentration of 400 μM (46 to 80.8 μg/mL; Table S2) exhibited appreciable binding affinity for *P. aeruginosa* PAO1 gDNA, as evidenced by the absence of detectable changes in ethidium bromide fluorescence (Figure S50).

#### 2.2.5. Effect of Amides on Motility of *Pseudomonas aeruginosa* PAO1

*Pseudomonas aeruginosa* colonizes surfaces in vitro through either biofilm formation or swarming motility. The selection of these behaviors is influenced by the physical properties of the surface, the availability of specific nutrients, and is tightly regulated by complex signaling networks [62]. In addition to QS, the initiation of biofilm formation by *P. aeruginosa* depends on two critical cell-associated structures: the flagellum and type IV pili. The flagellum enables swimming motility, while type IV pili mediate twitching motility. Both types of motility are essential during the early stages of biofilm development, facilitating initial surface attachment and microcolony formation [52]. To assess whether the amides **1**–**9** affect any of these motility mechanisms, we examined their impact on swimming, swarming, and twitching motilities. Our results indicate that none of the tested amides affected any of the motility types exhibited by *P. aeruginosa* at a concentration of 250 μg/mL (Figure S51). Therefore, the observed stimulation of biofilm formation in the presence of these amides cannot be attributed to enhanced swarming or twitching motility. In contrast, previous study reported that *N*-acetyltyramine (**7**), at a fourfold higher concentration (1 mg/mL), inhibited swimming motility of *P. aeruginosa* PAO1 by 40% and 23%, respectively, while having no observable effect on twitching motility [15]. Interestingly, tyramine at the same concentration reduced swimming, swarming, and twitching motilities by approximately 50%.

### 2.3. Modulation of QS in Chromobacterium violaceum and Serratia marcescens

Finally, the impact of amides **1**–**9** on QS systems in other bacterial species was evaluated based on changes in pigment production in *Chromobacterium violaceum* CV026 and *Serratia marcescens* ATCC 27117. *Chromobacterium violaceum* CV026 is a violacein-deficient mutant that appears white due to the absence of endogenous violacein production; however, it synthesizes violacein in response to exogenously supplied AHLs, making it a widely used biosensor for QS modulation [63]. In this assay, only *N*-acetyltryptamine (**8**) showed QS-inhibitory activity, as evidenced by the formation of colorless (but not transparent) zones around compound-loaded discs (250 µg/disc), indicating interference with violacein synthesis (Figure S52). At twice the concentration (500 µg/disc), branched homologues of amide **6**, 2-methyl-*N*-(2-phenylethyl)propanamide and 3-methyl-*N*-(2-phenylethyl)butanamide, demonstrated strong suppression of violacein production in both *C. violaceum* CV026 and *C. violaceum* ATCC 12472 [64]. The authors suggested that the structural similarity and comparable molecular sizes of these phenethylamides to AHL autoinducers may enable them to act as AHL mimics, competitively binding to quorum-sensing receptors. Previous study has also shown that *N*-acetyltyramine (**7**) can inhibit violacein production in *C. violaceum* ATCC 12472 by approximately 75%, at a concentration of 500 µg/mL, as measured by spectrophotometric assay [15].

The potential impact of amides **1**–**9** on prodigiosin biosynthesis in *S. marcescens* ATCC 27117 was also assessed using a disc diffusion assay. None of the compounds tested showed measurable inhibition of prodigiosin production under the experimental conditions (Figure S53).

### 2.4. Safety of Amides ***1***–***9*** Toward Eukaryotic and Prokaryotic Cells

We have previously found that crude culture extracts of the NP10 strain exhibited antimicrobial activity in a disc-diffusion assay (200 μg per disc) against various microorganisms, including *Saccharomyces cerevisiae* FAV20, *S. cerevisiae* FAS20, *Candida albicans*, *Enterococcus faecalis*, *Staphylococcus aureus*, *Bacillus subtilis*, and *Micrococcus luteus*. Additionally, these extracts displayed a mild cytotoxic effect on human fibroblasts and melanoma cell lines, but this effect was only evident at a high concentration of 1 mg/mL [11].

**Figure 5:**
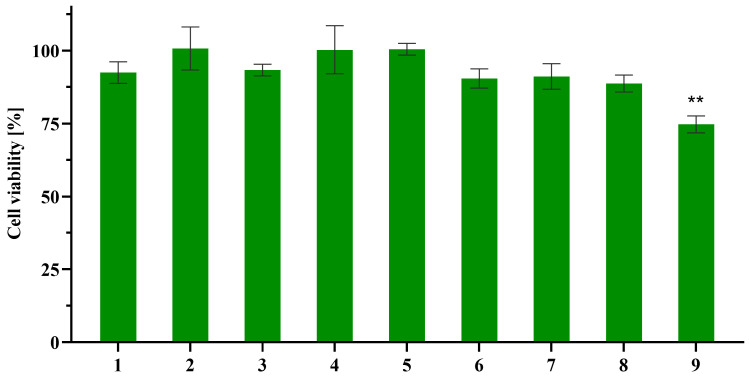
Cytotoxicity of amides **1**–**9** against normal MRC-5 fibroblast cells at 100 μM (11.5–20.2 μg/mL). Data are presented as mean ± SD (*n* = 2 × 4). Statistical analysis was performed using one-way ANOVA followed by Dunnett’s multiple comparisons test (vs. control). ** *p* < 0.01 indicate statistically significant differences compared to control.

Therefore, the in vitro cytotoxicity of amides **1**–**9** was initially assessed against normal human MRC-5 fibroblast cells at three concentrations (25, 50, and 100 μM) using the 3-(4,5-dimethylthiazol-2-yl)-2,5-diphenyltetrazolium bromide (MTT) assay, to evaluate their potential interaction with eukaryotic cells. Among the tested compounds, only the primary amide **9** derived from 2-phenylacetic acid exhibited a mild cytotoxic effect, reducing cell viability by approximately 25% at the highest concentration (100 μM, i.e., 13.5 μg/mL; Figure 5). These results are consistent with our findings regarding the crude extract and previous studies indicating that *N*-(2-phenylethyl)acetamide (**6**) and *N*-acetyltyramine (**7**) exhibit no cytotoxic effects against the A549 human non-small lung cancer cell line and the P388/ADR doxorubicin-resistant murine leukemia cell line, respectively [65,66].

Regarding the bioactivity of the amides described here, the antimicrobial activity of *N*-acetyltyramine (**7**) and *N*-acetyltryptamine (**8**) has been the most extensively studied [28,29,30,31,32,67]. Nevertheless, the reported outcomes remain inconclusive and, at times, contradictory. For example, Driche et al. reported significant antibacterial activity of acetamide **7** against *Escherichia coli* with a minimum inhibitory concentration (MIC) of 20 μg/mL [28]. In contrast, Yang et al. observed no inhibitory effect at a concentration of 200 μg/mL [67]. Similarly, for compound **8**, Ben Ameur Mehdi et al. observed antistaphylococcal activity at 10 μg/disc [30], whereas Zhang et al. reported an absence of activity at 30 μg/disc [31]. Herein, the antimicrobial activity of amides **1**–**9** was assessed using the disk diffusion method against two Gram-positive bacteria (*S. aureus* and *Klebsiella pneumoniae*), two Gram-negative bacteria (*E. coli* and *P. aeruginosa*), and one fungal strain (*C. albicans*). However, no inhibitory effect on microbial growth was observed even at a concentration of 250 μg per disk. These discrepancies in the observed antimicrobial activity may be attributed to variations in experimental protocols, bacterial strains, or compound purity, underscoring the necessity for standardized methodologies to accurately assess the antimicrobial efficacy of these compounds. We note that prior reports often used different strains/media or higher loads; harmonized protocols are needed for cross-study comparisons.

## 3. Materials and Methods

### 3.1. General Experimental Procedures

All used chemicals and solvents were obtained from commercial sources (e.g., Sigma-Aldrich, St. Louis, MO, USA; Merck, Darmstadt, Germany; Fisher Scientific, Waltham, MA, USA) and used as received, except for the solvents, which were distilled and dried before use. Silica gel 60, particle size distribution 40–63 mm (Acros Organics, Geel, Belgium), was used for dry-column vacuum chromatography, whereas Al silica gel plates precoated with silica gel 60 F_254_ (Merck, Darmstadt, Germany), were used for analytical TLC analyses. Visualization of TLC plates was performed under UV light (254 nm) and/or by spraying the plates with a 1:1 (*v*/*v*) aqueous sulfuric acid solution, followed by brief heating. The elemental analyses were carried out in a Vario EL III Elemental analyzer (ElementarAnalysensysteme GmbH, Hanau, Germany) to determine C, H, and N. ATR-IR measurements (attenuated total reflectance) were recorded on a Thermo Nicolet model 6700 FTIR instrument (Waltham, MA, USA). High-resolution mass spectrometry (HRMS) analysis was performed using an MStation JMS-700 mass spectrometer (JEOL, Peabody, MA, USA) with ionization energy of 70 eV, an ionization trap current of 300 μA, and a source temperature of 230 °C.

### 3.2. Gas Chromatography-Mass Spectrometry (GC-MS) Analyses

GC-MS analyses (3 repetitions) were carried out using a Hewlett-Packard 6890N gas chromatograph equipped with a fused silica capillary column DB-5MS (5% polydiphenylsiloxane and 95% polydimethylsiloxane, 30 m × 0.25 mm, film thickness 0.25 µm, Agilent Technologies, Palo Alto, CA, USA) and coupled with a 5975B mass selective detector from the same company. The injector and interface were operated at 250 and 300 °C, respectively. The oven temperature was raised from 70 to 290 °C at a heating rate of 5 °C/min, and the program ended with an isothermal period of 10 min. Helium was used as a carrier gas at 1.0 mL/min. The samples, 1.0 µL of extract/pure compound solutions in diethyl ether (ca. 1 mg of sample per 1.0 mL of solvent), were injected in a pulsed split mode (the flow was 1.5 mL/min for the first 0.5 min and then set to 1.0 mL/min throughout the remainder of the analysis; split ratio 40:1). MS conditions were as follows: ionization voltage 70 eV, acquisition mass range *m*/*z* 35–650, scan time 0.32 s. The constituents were initially identified by comparing their linear retention in_d_ices (relative to C_7_–C_40_ *n*-alkanes on a DB-5MS column) with values reported in the literature, and their mass spectra with those from the Wiley 11, NIST17, MassFinder 2.3, and an in-house MS library. Unambiguous identification was confirmed by co-injection with authentic reference standards.

### 3.3. NMR Measurements

^1^H and ^13^C NMR spectra were recorded on a Bruker Avance III 400 MHz NMR spectrometer (Fällanden, Switzerland; ^1^H at 400 MHz, ^13^C at 100.6 MHz), equipped with a 5 mm dual ^13^C/^1^H probe head at 20 °C. All the NMR spectra were recorded in CDCl_3_ or DMSO-*d*_6_ with tetramethylsilane (TMS) as an internal standard. Chemical shifts (δ) are reported in ppm and referenced to tetramethylsilane (δ_H_ = 0.00 ppm), or the (residual) solvent signal (CHCl_3_ or DMSO-*d*_5_), and ^13^CDCl_3_, in ^1^H NMR and ^13^C NMR and heteronuclear 2D spectra, respectively. Scalar couplings are reported in Hertz (Hz). The acquired NMR experiments, both 1D and 2D, were recorded using standard Bruker built-in pulse sequences.

The values of chemical shifts and coupling constants were determined by a simulation of the ^1^H NMR spectrum (manual iterative full spin analysis). ^1^H NMR full spin analysis of all products was performed by manually adjusting δ_H_ and *J* values to fit the experimentally available values and further optimized using MestReNova 11.0.3 software (Tools/Spin Simulation) [68]. This procedure led to a systematic refinement of all calculated NMR parameters until the simulation outcome was in excellent agreement (NRMSD < 0.05%) with these experimental data from the synthesized compounds. Four and two decimal places for δ_H_ and *J* values, respectively, were considered significant in these simulation experiments.

### 3.4. Preparation of Crude Extracts from NP10 Culture

Crude culture extracts of *Streptomyces* sp. NP10 were prepared by growing the strain in 500 mL of MSY medium (maltose 30 g/L, tryptic soy broth 8 g/L, yeast extract 4 g/L, CaCO_3_ 2 g/L, NaNO_3_ 3 g/L, MnSO_4_ × 7H_2_O 0.6 g/L, ZnSO_4_ 0.005 g/L, FeSO_4_ × 7H_2_O 0.3 g/L, CoCl_2_ × 7H_2_O 5 mg/L) in a 2.5 L Erlenmeyer flask (1:5 culture-to-flask volume ratio) containing a coiled stainless-steel spring to enhance aeration. Cultivation was performed for 6 days at 30 °C with shaking at 180 rpm. Upon incubation, the whole culture (mycelium and supernatant) was extracted with an equal volume of ethyl acetate (1:1, *v*/*v*) by vigorous mixing at room temperature for 60 min. Phase separation was achieved by centrifugation at 5000× *g* for 10 min at 25 °C using a Sorvall RC-5B Super Speed Centrifuge (Du Pont Instruments, Wilmington, DE, USA). The ethyl acetate phase was dried over Na_2_SO_4_, filtered, and concentrated under reduced pressure at 40 °C using a BUCHI Rotavapor R-300 (BÜCHI Labortechnik AG, Flawil, Switzerland) to obtain the crude extract as a powder.

### 3.5. Synthesis of Acetamides ***1***–***3*** and ***5***–***8***

Acetamides **1**–**3** and **5**–**8** were prepared using a general method based on the Schotten–Baumann reaction [69,70]. To a solution of the corresponding amine (1 eq, 2 to 23.5 mmol) in dichloromethane (DCM) under a N_2_ atmosphere at 0 °C, Et_3_N (1 eq) was added dropwise, followed by a solution of AcCl (1 eq) in DCM. The reaction mixture was stirred overnight at room temperature, then quenched with water and subsequently washed with 5% (*v*/*v*) aqueous HCl and 5% (*w*/*v*) aqueous NaHCO_3_. The organic layer was dried with anhydrous MgSO_4_, filtered, and concentrated under reduced pressure. The crude products were purified by gradient dry flash chromatography on silica gel using a stepwise elution system consisting of pure hexane, 50% Et_2_O in hexane (*v*/*v*), pure Et_2_O, and MeOH. The purity of the acetamides was checked by TLC and GC-MS. Due to its lower solubility, tyramine was reacted in a DCM/THF mixture (2:1, *v*/*v*) as the reaction solvent.

*N*-(2-Methylpropyl)acetamide (**1**): yield 809 mg (28%); retention index (RI) = 1024 (DB-5MS column); FTIR (neat; cm^−1^) 3287 (m; *ν*(N–H)), 2958 (w; *ν*(CH_3_)_as_), 2871 (w; *ν*(CH_3_)_s_), 1633 (s; *ν*(C=O), amide I), 1550 (s, amide II), 1435 (m, δ_s_(CH_2_)), 1372 (m), 1285 (m, amide III), 1159 (w), 1096 (w), 726 (br, amide V), 601 (m); MS (EI), *m*/*z* (%) 115 (29.6) [M^+^], 100 (60.3), 73 (21.4), 72 (92.1), 60 (47.4), 58 (44.6), 43 (100), 41 (27.1); analyzed C 62.43, H 11.30, N 12.25 calculated for C_6_H_13_NO, C 62.57, H 11.38, N 12.16, O 13.89%; ^1^H NMR (CDCl_3_) δ 5.9450 (br t, *J* = 6.15 Hz, 1H, NH), 3.0653 (dd, *J* = 6.88, 6.15 Hz, 2H, CH_2_), 1.9946 (s, 3H, CH_3_CO), 1.7676 (t septet, *J* = 6.88, 6.67 Hz, 1H, CH), 0.9148 (d, *J* = 6.67 Hz, 6H, CH(CH_3_)_2_); ^13^C NMR (CDCl_3_) δ 170.3 (m, C=O), 47.0 (tm, *J* = 137.6 Hz, CH_2_), 28.4 (d pseudo-nonet, *J* = 127.5, 3.8 Hz, CH), 23.3 (q, *J* = 127.8 Hz, CH_3_CO), 20.1 (qm, *J* = 124.8 Hz, CH(CH_3_)_2_); HRMS (EI) calcd for C_6_H_13_NO^+^: 115.0992, found 115.0999.

*N*-(2-Methylbutyl)acetamide (**2**): yield 671 mg (23%); retention index (RI) = 1128 (DB-5MS column); FTIR (neat; cm^−1^) 3285 (m; *ν*(N–H)), 2960 (w; *ν*(CH_3_)_as_), 2929 (w; *ν*(CH_2_)_as_), 2875 (w; *ν*(CH_3_)_s_), 1634 (s; *ν*(C=O), amide I), 1556 (s, amide II), 1435 (m, δ_s_(CH_2_)), 1374 (m), 1291 (m, amide III), 1148 (w), 1096 (w), 727 (br, amide V), 603 (m); MS (EI), *m*/*z* (%) 129 (10.4) [M^+^], 114 (9.2), 100 (60.2), 86 (3.1), 73 (70), 72 (100), 60 (68), 58 (31.1), 56 (14.8), 55 (18.5), 43 (66), 42 (19.8), 41 (31.6); analyzed C 64.94, H 11.79, N 10.81 calculated for C_7_H_15_NO, C 65.07, H 11.70, N 10.84, O 12.38%; ^1^H NMR (CDCl_3_) δ 5.8035 (br dd, *J* = 6.10, 6.00 Hz, 1H, NH), 3.1742 (ddd, *J* = −13.30, 6.30, 6.00 Hz, 1H, NCH_A_H_M_), 3.0485 (ddd, *J* = −13.30, 7.30, 6.10 Hz, 1H, NCH_A_H_M_), 1.9863 (s, 3H, CH_3_CO), 1.5358 (ddqdd, *J* = 8.00, 7.30, 6.45, 6.30, 5.22 Hz, 1H, CH), 1.3928 (dqd, *J* = −13.55, 7.45, 5.22 Hz, 1H, CH_A_H_M_CH_3_), 1.1385 (ddq, *J* = −13.55, 8.00, 7.45 Hz, 1H, CH_A_H_M_CH_3_) 0.8945 (pseudo t, *J* = 7.45, 7.45 Hz, 3H, CH_A_H_B_CH_3_), 0.8865 (d, *J* = 6.45 Hz, 3H, CHCH_3_); ^13^C NMR (CDCl_3_) δ 170.3 (m, C=O), 45.3 (tm, *J* = 136.4 Hz, NCH_2_), 34.8 (dm, *J* = 122.6 Hz, CH), 27.0 (tm, *J* = 124.5 Hz, CH_2_CH_3_), 23.3 (q, *J* = 127.8 Hz, CH_3_CO), 17.1 (q pseudo-sextet, *J* = 125.2, 4.7 Hz, CH_3_CH), 11.3 (q pseudo-q, *J* = 124.5, 3.8 Hz, CH_2_CH_3_); HRMS (EI) calcd for C_7_H_15_NO^+^: 129.1149, found 129.1143.

*N*-(3-Methylbutyl)acetamide (**3**): yield 992 mg (34%); retention index (RI) = 1135 (DB-5MS column); FTIR (neat; cm^−1^) 3283 (m; *ν*(N–H)), 2956 (w; *ν*(CH_3_)_as_), 2929 (w; *ν*(CH_2_)_as_), 2870 (w; *ν*(CH_3_)_s_), 1633 (s; *ν*(C=O), amide I), 1556 (s, amide II), 1435 (m, δ_s_(CH_2_)), 1367 (m), 1295 (m, amide III), 1170 (w), 1096 (w), 729 (br, amide V), 603 (m); MS (EI), *m*/*z* (%) 129 (9.6) [M^+^], 114 (23.3), 86 (35.3), 73 (100), 72 (81), 55 (20.6), 44 (49.5), 43 (90.8), 42 (18.3), 41 (25.6); analyzed C 65.19, H 11.77, N 10.90 calculated for C_7_H_15_NO, C 65.07, H 11.70, N 10.84, O 12.38%; ^1^H NMR (CDCl_3_) δ 5.7640 (br t, *J* = 5.60 Hz, 1H, NH), 3.2539 (AA′XX′, *J* = −13.50, 8.95, 6.10, 5.60 Hz, 2H, NCH_2_CH_2_), 1.9761 (s, 3H, CH_3_CO), 1.6175 (t septet, *J* = 6.85, 6.65 Hz, 1H, CH), 1.3909 (AA′XX′, *J* = −13.50, 8.95, 6.85, 6.10 Hz, 2H, NCH_2_CH_2_), 0.9139 (d, *J* = 6.65 Hz, 6H, CH(CH_3_)_2_); ^13^C NMR (CDCl_3_) δ 170.3 (m, C=O), 38.4 (tm, *J* = 124.5 Hz, NCH_2_CH_2_), 38.0 (t pseudo-p, *J* = 137.7, 3.6 Hz, NCH_2_), 25.8 (d pseudo-nonet, *J* = 124.4, 3.8 Hz, CH), 23.3 (q, *J* = 127.8 Hz, CH_3_CO), 22.4 (qm, *J* = 124.6 Hz, CH(CH_3_)_2_); HRMS (EI) calcd for C_7_H_15_NO^+^: 129.1149, found 129.1145.

*N*-Benzylacetamide (**5**): yield 1.76 g (42%); retention index (RI) = 1443 (DB-5MS column); FTIR (neat; cm^−1^) 3282 (m; *ν*(N–H)), 3086, 3064 and 3027 (w; *ν*(arC–H)), 2931 (w; *ν*(CH_2_)_as_), 1636 (s; *ν*(C=O), amide I), 1548 (s, amide II), 1491 (m, *ν*(arC=Car)), 1427 (m, δ_s_(CH_2_)), 1362 (m), 1287 (m, amide III), 1073 (w), 1026 (w), 746 (m, γ(C–H), 727 (m, amide V), 692 (s, γ(C–H), 612 (m); MS (EI), *m*/*z* (%) 149 (77.4) [M^+^], 107 (17.2), 106 (100), 91 (30.5), 79 (14.9), 77 (15.3), 65 (8), 51 (8.9), 43 (18.6); analyzed C 72.31, H 7.40, N 9.33 calculated for C_9_H_11_NO, C 72.46, H 7.43, N 9.39, O 10.72%; ^1^H NMR (CDCl_3_) δ 7.3216 (AA′BB′C, *J* = 8.15, 7.40, 1.50, 0.35 Hz, 2H, H-3 and H-5 in Ph group), 7.2696 (AA′BB′C, *J* = 7.40, 7.40, 1.20, 1.20 Hz, 1H, H-4 in Ph group), 7.2625 (AA′BB′C, *J* = 8.15, 2.00, 1.20, 0.35, −0.35 Hz, 1H, H-2 and H-6 in Ph group), 6.1055 (br t, *J* = 5.75 Hz, 1H, NH), 4.3956 (dt, *J* = 5.75, −0.35 Hz, 2H, CH_2_), 1.9925 (s, 3H, CH_3_CO); ^13^C NMR (CDCl_3_) δ 170.1 (m, C=O), 138.2 (m, C-1 in Ph group), 128.7 (dd, *J* = 160.2, 7.5 Hz, C-3 and C-5 in Ph group), 127.8 (d pseudo-p, *J* = 152.7, 5.5 Hz, C-2 and C-5in Ph group), 127.5 (dt, *J* = 160.5, 7.3 Hz, C-4 in Ph group), 43.7 (t pseudo-q, *J* = 138.6, 3.6 Hz, CH_2_), 23.2 (q, *J* = 127.8 Hz, CH_3_CO). The carbon atom numbering scheme for the monosubstituted phenyl ring in amide **5** follows IUPAC nomenclature conventions; HRMS (EI) calcd for C_9_H_11_NO^+^: 149.0836, found 149.0839.

*N-*(2-Phenylethyl)acetamide (**6**): yield 2.27 g (44%); retention index (RI) = 1513 (DB-5MS column); FTIR (neat; cm^−1^) 3283 (m; *ν*(N–H)), 3086, 3064 and 3032 (w; *ν*(arC–H)), 2928 (w; *ν*(CH_2_)_as_), 2868 (w; *ν*(CH_3_)_s_), 1640 (s; *ν*(C=O), amide I), 1550 (s, amide II), 1496 (m, *ν*(arC=Car)), 1435 (m, δ_s_(CH_2_)), 1364 (m), 1295 (m, amide III), 1197 (w), 1030 (w), 746 (s, γ(C–H), 699 (s, γ(C–H), 602 (m); MS (EI), *m*/*z* (%) 163 (21.7) [M^+^], 120 (1.9), 105 (10.7), 104 (100), 103 (6.9), 91 (25.5), 78 (6), 77 (6.8), 72 (13.7), 65 (12.9), 43 (22.2); analyzed C 73.72, H 8.08, N 8.63 calculated for C_10_H_13_NO, C 73.59, H 8.03, N 8.58, O 9.80%; ^1^H NMR (CDCl_3_) δ 7.3268 (AA′BB′C, *J* = 7.60, 7.30, 1.80, 0.65 Hz, 2H, H-3 and H-5 in Ph group), 7.2497 (AA′BB′C, *J* = 7.30, 7.30, 1.25, 1.25 Hz, 1H, H-4 in Ph group), 7.2098 (AA′BB′C, *J* = 7.60, 2.20, 1.25, 0.65, −0.40 Hz, 2H, H-2 and H-6 in Ph group), 5.7265 (br t, *J* = 6.09 Hz, 1H, NH), 3.5204 (td, *J* = 6.95, 6.09 Hz, 2H, NHCH_2_), 2.8298 (tt, *J* = 6.95, −0.40 Hz, 2H, PhCH_2_), 1.9487 (s, 3H, CH_3_CO); ^13^C NMR (CDCl_3_) δ 170.2 (m, C=O), 138.9 (m, C-4), 128.8 (d pseudo-p, *J* = 156.9, 6.4 Hz, C-2 and C-6 in Ph group), 128.7 (dd, *J* = 159.6, 7.8 Hz, C-3 and C-5 in Ph group), 126.5 (dt, *J* = 160.5, 7.4 Hz, C-4 in Ph group), 40.7 (t pseudo-q, *J* = 138.9, 5.7 Hz, NCH_2_), 35.6 (t pseudo-p, *J* = 128.1, 3.7 Hz, NCH_2_CH_2_), 23.3 (q, *J* = 127.8 Hz, CH_3_CO). The carbon atom numbering scheme for the monosubstituted phenyl ring in amide **6** follows IUPAC nomenclature conventions; HRMS (EI) calcd for C_10_H_13_NO^+^: 163.0992, found 163.0997.

*N*-Acetyltyramine (**7**): yield 360 mg (33%); retention index (RI) = 1874 (DB-5MS column); FTIR (neat; cm^−1^) 3326 (m; *ν*(O–H)), 3269 (w; *ν*(N–H)), 3097 (w; *ν*(arC–H)), 2934 (w; *ν*(CH_2_)_as_), 2868 (w; *ν*(CH_3_)_s_), 1613 (s; *ν*(C=O), amide I), 1592 (m, *ν*(arC=Car)), 1563 (s, amide II), 1513 (m, *ν*(arC=Car)), 1456 (m, δ_s_(CH_2_)), 1360 (m), 1243 (s, *ν*(C–O), 1171 (w), 1041 (w), 809 (s, γ(C–H), 710 (m, amide V); MS (EI), *m*/*z* (%) 179 (2.2) [M^+^], 121 (14.9), 120 (100), 107 (40.1), 77 (14.4), 65 (4.1), 53 (1.9), 51 (5.6), 43 (10.2), 42 (2.3); analyzed C 66.89, H 7.37,N 7.76 calculated for C_10_H_13_NO_2_, C 67.02, H 7.31, N 7.82, O 17.85%; ^1^H NMR (DMSO-*d*_6_) δ 9.0960 (br s, 1H, OH), 7.8680 (br t, *J* = 6.20 Hz, 1H, NH), 6.9790 (AA′XX′, *J* = 8.10, 2.40, 0.50 Hz, 2H, H-2 and H-6 in Ar group), 6.6728 (AA′XX′, *J* = 8.10, 2.40, 0.5 Hz, 2H, H-3 and H-5 in Ar group), 3.1735 (AA′XX′, *J* = −13.00, 8.40, 6.45, 6.20 Hz, 2H, NCH_2_), 2.5690 (AA′XX′, *J* = −13.40, 8.40, 6.45 Hz, 2H, ArCH_2_), 1.7778 (s, 3H, CH_3_CO); ^13^C NMR (DMSO-*d*_6_) δ 168.9 (m, C=O), 155.5 (tt, *J* = 9.3, 2.4 Hz, C-4 in Ar group), 129.4 (ddt, *J* = 155.6, 7.4, 5.2 Hz, C-2 and C-6 in Ar group), 129.3 (m, C-1 in Ar group), 115.0 (dd, *J* = 157.5, 4.6 Hz, C-3 and C-5 in Ar group), 40.5 (overlapped with DMSO, NCH_2_), 34.3 (t pseudo-p, *J* = 127.7, 3.6 Hz, ArCH_2_), 22.5 (q, *J* = 127.8 Hz, CH_3_CO). The carbon atom numbering scheme for the disubstituted phenyl ring in amide **7** follows IUPAC nomenclature conventions; HRMS (EI) calcd for C_10_H_13_NO_2_^+^: 179.0941, found 179.0938.

*N*-Acetyltryptamine (**8**): yield 151 mg (32%); retention index (RI) = 2196 (DB-5MS column); FTIR (neat; cm^−1^) 3399 (w; *ν*(N–H)),3269 (w; *ν*(N–H)), 3100 (w; *ν*(arC–H)), 2926 (w; *ν*(CH_2_)_as_), 1629 (s; *ν*(C=O), amide I), 1533 (s, amide II and *ν*(arC=Car)), 1431 (m, δ_s_(CH_2_)), 1367 (m), 1287 (m, amide III), 1228 (m), 1171 (w), 1094 (w), 1008 (w), 793 (s, γ(C–H); MS (EI), *m*/*z* (%) 202 (12) [M^+^], 144 (11.7), 143 (99), 131 (10.5), 130 (100), 115 (6.1), 103 (10.1), 102 (6.4), 77 (12), 43 (6.5); analyzed C 71.14, H 7.03, N 13.81 calculated for C_12_H_14_N_2_O, C 71.26, H 6.98, N 13.85, O 7.92%; ^1^H NMR (DMSO-*d*_6_) δ 10.8251 (br dt, *J* = 2.25, 0.80, 1H, NH in Ar group), 7.9815 (br t, *J* = 5.60, 1H, NH), 7.5269 (ddd, *J* = 8.10, 0.90, 0.85 Hz, 1H, H-4 in Ar group), 7.3431 (ddd, *J* = 8.15, 1.00, 0.85 Hz, 1H, H-7 in Ar group), 7.1501 (d, *J* = 2.25 Hz, 1H, H-2 in Ar group), 7.0672 (ddd, *J* = 8.15, 6.90, 0.90 Hz, 1H, H-6 in Ar group), 6.9825 (ddd, *J* = 8.10, 6.90, 1.00 Hz, 1H, H-5 in Ar group), 3.3231 (AA′XX′, *J* = −13.60, 8.40, 6.60, 5.60 Hz, 2H, NCH_2_), 2.8167 (AA′XX′, *J* = −13.50, 8.40, 6.60, 0.80 Hz, 2H, ArCH_2_), 1.8155 (s, 3H, CH_3_CO); ^13^C NMR (DMSO-*d*_6_) δ 169.6 (m, C=O), 136.7 (ddd, *J* = 10.7, 8.2, 3.6 Hz, C-7a in Ar group), 127.7 (m, C-3a in Ar group), 123.1 (d pseudo-q, *J* = 181.0, 5.0 Hz, C-2 in Ar group), 121.4 (dd, *J* = 157.7, 6.9 Hz, C-6 in Ar group), 118.7 (dd, *J* = 157.5, 7.7 Hz, C-4 and C-5 in Ar group), 112.3 (ddm, J = 5.8, 3,4 Hz, C-3 in Ar group), 111.8 (dd, *J* = 158.5, 7.9 Hz, C-7 in Ar group), 40.0 (overlapped with DMSO, NCH_2_), 25.7 (tm, *J* = 126.9 Hz, ArCH_2_), 23.2 (q, *J* = 127.8 Hz, CH_3_CO). The carbon atom numbering scheme for the monosubstituted indol ring in amide **8** follows IUPAC nomenclature conventions; HRMS (EI) calcd for C_12_H_14_N_2_O^+^: 202.1101, found 202.1105.

### 3.6. Synthesis of N-(3-Methyl-2-butenyl)acetamide (***4***)

Sodium hydride (340 mg, 60% dispersion in mineral oil) was washed with dry hexane (3 × 3 mL) under N_2_ immediately before use. The obtained NaH was suspended in dry THF (10 mL) and stirred vigorously under N_2_ in an acetone-cooled bath. A solution of acetamide (0.5 g) in dry THF (2 mL) was then added dropwise and stirred for 10 min at −78 °C, followed by an additional 10 min at 0 °C. Prenyl bromide (1.26 g, 0.98 mL) dissolved in dry THF (5 mL) was then added dropwise, and the reaction mixture was stirred at room temperature overnight. The reaction was quenched by the dropwise addition of water (10 mL), and the solvent was subsequently evaporated under reduced pressure. The aqueous residue was extracted with Et_2_O (3 × 30 mL), and the combined organic layers were dried with anhydrous MgSO_4_, filtered, and concentrated in vacuo to afford the crude product. Purification by dry flash chromatography on silica gel, employing a stepwise gradient elution system of pure hexane, 50% Et_2_O in hexane (*v*/*v*), pure Et_2_O, and MeOH, afforded pure *N*-(3-methylbut-2-en-1-yl)acetamide, as confirmed by NMR, GC-MS, and IR spectroscopy.

*N*-(3-Methyl-2-butenyl)acetamide (**4**): yield 247 mg (24%); retention index (RI) = 1181 (DB-5MS column); FTIR (neat; cm^−1^) 3268 (m; *ν*(N–H)), 3091 (w, *ν*(=C–H)), 2969 (w; *ν*(CH_3_)_as_), 2930 (w; *ν*(CH_2_)_as_), 1630 (s; *ν*(C=O), amide I), 1546 (s, amide II), 1434 (m, δ_s_(CH_2_)), 1367 (m), 1284 (m, amide III), 1080 (w), 740 (m), 606 (m); MS (EI), *m*/*z* (%) 127 (30.8) [M^+^], 112 (11.5), 98 (2.1), 84 (45.7), 70 (100), 67 (37.1), 60 (8.2), 53 (9.4), 43 (40); analyzed C 66.22, H 10.22, N 11.09 calculated for C_7_H_13_NO, C 66.11, H 10.30, N 11.01, O 12.58%; ^1^H NMR (400 MHz, CDCl_3_) δ 5.7470 (br t, *J* = 5.40 Hz, 1H, NH), 5.1519 (tqq, *J* = 7.10, 1.40, 1.30 Hz, 1H, CH), 3.7780 (dd, *J* = 7.10, 5.40 Hz, 2H, CH_2_), 1.9355 (s, 3H CH_3_CO), 1.6816 (br d, *J* = 1.30 Hz, 3H, CH_3_*^seqcis^*), 1.6310 (br d, *J* = 1.40 Hz, 3H, CH_3_*^seqtrans^*); ^13^C NMR (100.6 MHz, CDCl_3_) δ 170.1 (m, C=O), 136.5 (m, C), 130.2 (d pseudo-nonet, *J* = 154.7, 5.4 Hz, CH), 37.8 (t pseudo-t, *J* = 138.2, 2.5 Hz, CH_2_), 25.7 (qdq, *J* = 125.8, 8.5, 4.1 Hz, CH_3_*^seqtrans^*), 23.2 (q, *J* = 127.8 Hz, CH_3_CO), 17.9 (qdq, *J* = 125.5, 8.2, 4.1 Hz, CH_3_*^seqcis^*); HRMS (EI) calcd for C_7_H_13_NO^+^: 127.0992, found 127.0998.

### 3.7. Synthesis of 2-Phenylacetamide (***9***)

2-Phenylacetyl chloride was prepared by refluxing 2-phenylacetic acid (1.0 g) with SOCl_2_ (0.64 mL) for 2 h. Excess SOCl_2_ was removed under reduced pressure. The resulting crude acyl chloride was added dropwise to vigorously stirred concentrated aqueous ammonia (100 mL), maintained in an ice-salt cooling bath, resulting in the formation of a white precipitate of 2-phenylacetamide (**9**) [71]. The product was collected by filtration, dried, and purified by dry flash chromatography on silica gel using a stepwise gradient elution system consisting of pure hexane, 50% Et_2_O in hexane (*v*/*v*), pure Et_2_O, and MeOH.

2-Phenylacetamide (**9**): yield 389 mg (45%); retention index (RI) = 1389 (DB-5MS column); FTIR (neat; cm^−1^) 3348 and 3159 (m; *ν*(H–N–H)), 3062 and 3029 (w, *ν*(=C–H)),2969 (w; *ν*(CH_3_)_as_), 2930 (w; *ν*(CH_2_)_as_), 1634 (s; *ν*(C=O), amide I and δ_s_(NH_2_), amide II), 1496 (m, *ν*(arC=Car)), 1411 (s), 1283 (w), 1204 (w), 1030 (m), 746 (s, γ(C–H), 696 (s, γ(C–H); MS (EI), *m*/*z* (%) 135 (20.1) [M^+^], 93 (6.7), 92 (87.2), 91 (100), 90 (4.1), 89 (7.9), 77 (2.6), 65 (18), 63 (8.2), 51 (5.5), 44 (11.2); analyzed C 66.22, H 10.22, N 11.09 calculated for C_8_H_9_NO, C 71.09, H 6.71, N 10.36, O 11.84%; ^1^H NMR (CDCl_3_) δ 7.3518 (AA′BB′C, *J* = 7.60, 7.40, 1.40, 0.70 Hz, 2H, H-3 and H-5 in Ph group), 7.2957 (AA′BB′C, *J* = 7.40, 7.40, 1.25, 1.25 Hz, H-4 in Ph group), 7.2642 (AA′BB′C, *J* = 7.60, 2.10, 1.25, 0.70, −0.40 Hz, 2H, H-2 and H-6 in Ph group), 6.0450 (br s, 1H, NH_A_H_X_), 5.1650 (br s, 1H, NH_A_H_X_), 3.5630 (br t, *J* = −0.40 Hz, 2H, CH_2_); ^13^C NMR (CDCl_3_) δ 174.2 (m, C=O), 134.8 (m, C-1 in Ph group), 129.4 (d pseudo-pentet, *J* = 158.4, 5.8 Hz, C-2 and C-6 in Ph group), 129.0 (dd, *J* = 160.6, 7.6 Hz, C-3 and C-5 in Ph group), 127.5 (dt, *J* = 160.7, 7.5 Hz, C-4 in Ph group), 43.3 (tm, *J* = 127.8 Hz, CH_2_). The carbon atom numbering scheme for the monosubstituted phenyl ring in amide **9** follows IUPAC nomenclature conventions; HRMS (EI) calcd for C_8_H_9_NO^+^: 135.0679, found 135.0677.

### 3.8. Antiproliferative Effect

The antiproliferative effect of amides **1**–**9** was evaluated using the previously described method [72]. Human lung fibroblast MRC5 cells (obtained from ATCC) were seeded in 96-well flat-bottom plates at a density of 1 × 10^4^ cells per well. The cells were cultured in RPMI-1640 medium supplemented with 100 µg/mL streptomycin, 100 U/mL penicillin, and 10% (*v*/*v*) fetal bovine serum, and maintained as monolayer cultures in a humidified atmosphere of 95% air and 5% CO_2_ at 37 °C. After 24 h of incubation, cells were treated with three different concentrations (25, 50, and 100 μM) of each test compound for 48 h. Cytotoxicity was assessed using the MTT reduction assay. Optical density was measured at 540 nm using a Tecan Infinite 200 microplate reader (Tecan Group, Männedorf, Switzerland). The MTT viability assay was performed twice, each in quadruplicate, and results were expressed as the percentage viability relative to the untreated control, which was arbitrarily set to 100%. Cisplatin served as a positive control, while DMSO and growth medium were used as negative controls.

### 3.9. Antimicrobial Effect—Disk Diffusion Assay

The antimicrobial properties of the compounds were evaluated against five microbial strains: two Gram-positive bacteria (*Staphylococcus aureus* ATCC 25923 and *Klebsiella pneumoniae* ATCC 13883), two Gram-negative bacteria (*Escherichia coli* NCTC 9001 and *Pseudomonas aeruginosa* PAO1 NCTC 10332), and one fungal strain (*Candida albicans* ATCC 10231). All strains were obtained from recognized culture collections (ATCC—American Type Culture Collection; NCTC—National Collection of Type Cultures). Bacterial strains were cultured on LB agar plates, while the fungal strain was grown on Sabouraud dextrose agar (SAB: 40 g/L glucose, 10 g/L peptone, 20 g/L agar) at 37 °C overnight.

Antimicrobial activity was assessed using the disc diffusion method, following a standard procedure [73]. Compounds **1**–**9** were dissolved in DMSO and applied to sterile filter paper discs at a concentration of 250 μg per disc. DMSO alone was used as the negative control. Inoculated plates were incubated at 37 °C for 24 h before measuring the zones of inhibition. The experiment was performed in triplicate and repeated two times.

### 3.10. Antimicrobial Effect—Microdilution Method

To determine minimal inhibitory concentrations (MIC) of each amide against P. aeruginosa PAO1, the standard broth microdilution method has been used in Luria–Bertani (LB) broth [74]. The final inoculum density was adjusted to 5 × 10^5^ colony-forming units per milliliter (CFU/mL). Stock solutions of the amides (5 mg/mL) were prepared in DMSO. The compounds were tested at seven concentrations ranging from 7.5 to 500 μg/mL. DMSO was included as a negative control. After 24 h of incubation at 37 °C, bacterial growth was assessed by measuring optical density at 600 nm (OD_600_) using a Tecan Infinite 200 multiplate reader (Tecan Group, Männedorf, Switzerland). Each assay was performed in triplicate and independently repeated twice.

### 3.11. Static Biofilm Formation Assay

Biofilm quantification assays were performed in microtiter plates using a crystal violet (CV) staining method to evaluate adherent cells [75]. Overnight cultures of *P. aeruginosa* PAO1 strain were inoculated in Tryptic Soy Broth (TSB) medium at 37 °C, with and without the tested compounds a 96-well microtiter plate. After 24 h, free (detached) cells were removed, and wells were washed with Phosphate-Buffered Saline (PBS). Biofilms were fixed with 100 mL of 99% (*v*/*v*) MeOH, followed by staining with 0.4% CV (*v*/*v*). After washing, CV was solubilized with 150 μL of glacial acetic acid (33%, *v*/*v*) and the OD of samples was measured at 590 nm using a Tecan Infinite 200 multiplate-reader (Tecan Group, Männedorf, Switzerland). The experiment was performed in quintuplicate and repeated two times. Parallel wells without CV staining were used to record OD_600_; biofilm signals were interpreted only when growth was not significantly altered vs. DMSO (ANOVA with Dunnett’s test, α = 0.05).

### 3.12. Effect on Bacterial Pigments Production

The effect of the amides on bacterial pigment production was evaluated as previously described [63]. Specifically, their influence on violacein synthesis was assessed using the *Chromobacterium violaceum* CV026 indicator strain. Briefly, semisolid LB agar (0.3% *w*/*v*, 5 mL) was seeded with 50 μL of an overnight culture of *C. violaceum* CV026 supplemented with *N*-hexanoyl-L-homoserine lactone (final concentration: 5 μM) and then poured over the surface of standard LB agar plates. Once the overlay was solidified, sterile filter paper discs containing 250 μg of each test compound were placed onto the plates. Petri dishes were incubated upright at 30 °C overnight. Inhibition of violacein synthesis was indicated by the appearance of white halos surrounding the discs on the otherwise purple bacterial lawn.

An overnight culture of *Serratia marcescens* ATCC 27117 was diluted 100-fold in molten semi-solid LB agar (0.3% *w*/*v*) and poured over solid LB medium. Cellulose discs impregnated with 250 μg of each test compound were placed on solidified agar and incubated for 24 h at 30 °C or 37 °C. Inhibition of prodigiosin synthesis was determined by the absence of red pigmentation around the discs, indicating disruption of pigment biosynthesis.

A pyocyanin assay was performed with *P. aeruginosa* PAO1 as reported [76]. Pyocyanin in the supernatant was quantified using a UV−vis spectrophotometer Ultrospec 3300pro (Amersham Biosciences, Piscataway, NJ, USA) at 695 nm. The experiment was performed in triplicate and repeated at least three times.

### 3.13. Effects on AHLs and AHQs Production

The production of long-chain and short-chain *N*-acyl homoserine lactones (AHLs) and 2-alkyl-4-quinolones (AHQs) was detected and quantified using bioluminescent *Pseudomonas aeruginosa* biosensor strains. Specifically, *P. aeruginosa* PA14-R3Δ*lasIPrsaI::lux* was used to monitor 3-oxo-C12-HSL levels, *P. aeruginosa* PAO1 Δ*rhlIpKD-rhlA::lux* was used to assess C4-HSL production, and *P. aeruginosa* PAO1 Δ*pqsAmini-CTXluxPpqsA* was employed to measure AHQs production, as previously described [63,75].

Briefly, overnight cultures of these biosensor strains were diluted to an optical density at 600 nm (OD_600_) of 0.045 and incubated with the tested amides (250 or 50 μg/mL) in the presence of the corresponding specific autoinducers (final concentration of 5 μM) for 4 h at 37 °C on a rotary shaker set to 70 rpm. After incubation, OD_600_ and bioluminescence were measured simultaneously using a Tecan Infinite 200 multiplate reader (Tecan Group, Männedorf, Switzerland). Luminescence values were normalized to cell density and expressed as a percentage relative to the corresponding untreated control. All experiments were performed in triplicate and repeated at least three times.

### 3.14. Motility Assays

The effect of the amides **1**–**9** on various types of *Pseudomonas aeruginosa* PAO1 motility was evaluated as previously described [76]. Briefly, swarming motility was evaluated on M8 agar plates (0.6% *w*/*v*) supplemented with either the test compound or DMSO as a control. Each plate was point-inoculated with 2.5 μL of *Pseudomonas* culture adjusted to an OD_620_ of 0.2. Following incubation at 37 °C for 16 h, swarming activity was assessed by visual inspection and measurement of the migration diameter.

Swimming motility was assessed using an overnight *Pseudomonas* culture standardized to an OD_620_ of 0.2. Cultures were point-inoculated onto M8 medium plates (5 g/L Na_2_HPO_4_, 3 g/L KH_2_PO_4_, 0.5 g/L NaCl, 0.2% glucose, 0.5% casamino acids, 1 mM MgSO_4_), solidified with 0.3% (*w*/*v*) agar, and supplemented with the test compound. Following incubation at 37 °C for 18 h, swimming behavior was evaluated by measuring the diameter of the migration zone surrounding the inoculation point.

Twitching motility was assessed by inoculating an overnight *Pseudomonas* culture into LB agar plates (1% agar) containing the test compound, using a sterile toothpick. Plates were incubated at 37 °C for 20 h, followed by an additional 72 h incubation at 25 °C. After agar removal, bacterial migration along the underlying plastic surface was visualized by staining with 2% (*v*/*v*) CV, and the diameter of the stained zone was measured.

### 3.15. In Vitro DNA Interaction by Gel Electrophoresis Assay

Genomic DNA (gDNA) from *P. aeruginosa* PAO1 was purified with a DNeasy tissue kit (Qiagen, Hilden, Germany). The quality and the concentration of DNA were estimated by measuring UV absorbance with a NanoVue Plus spectrophotometer (GE Healthcare, Freiburg, Germany). The ability of amides **1**–**9** to bind gDNA from *P. aeruginosa* PAO1 was examined by using agarose gel electrophoresis [77]. For the gel electrophoresis experiments, gDNA (370 ng) was treated with the investigated compounds (400 μM) in phosphate buffer (pH 7.4), and the contents were incubated for 12 h at 37 °C, then subjected to gel electrophoresis on 0.8% (*w*/*v*) agarose gel containing 0.1 μg/mL of ethidium bromide in TAE buffer (40 mM Tris acetate/1 mM EDTA, pH 7.4) buffer at 60 V for 2 h. Gels were visualized and analyzed using the Gel Doc EZ system (Bio-Rad, Life Sciences, Hercules, CA, USA), equipped with the Image Lab™ Software 6.0.

### 3.16. Statistical Analyses

Results were expressed as mean ± standard deviation (SD). Statistical significance was determined using one-way analysis of variance (ANOVA), followed by Dunnett’s multiple comparisons test (GraphPad Prism, version 8.0.2, San Diego, CA, USA). Differences with *p* < 0.05 were considered statistically significant.

## 4. Conclusions

This study underscores the potential ecological role of volatile amides (**1**–**9**) released by *Streptomyces* sp. NP10 as modulators of QS and biofilm formation in *Pseudomonas aeruginosa* PAO1. Through comprehensive GC-MS profiling, supported by synthesis and spectral confirmation, nine minor volatile amides were identified, primarily as acetylated derivatives of common BAs. Among them, *N*-(3-methyl-2-butenyl)acetamide (**4**) is described here for the first time as a natural product, while *N*-(2-methylbutyl)acetamide (**2**) is newly identified as a *Streptomyces*-derived microbial metabolite. These findings expand the known chemical space of bacterial secondary metabolism. Detailed iterative simulation of their ^1^H NMR spectra enabled the resolution of complex splitting patterns and precise determination of chemical shifts and coupling constants, including long-range couplings.

At subinhibitory concentrations (250 μg/mL), all amides except *N*-acetyltryptamine (**8**) significantly stimulated biofilm formation in *P. aeruginosa* PAO1, with compounds **2**, **4** and **9** showing the strongest effects. Simultaneously, most amides also suppressed QS-regulated signaling, including both AHL and AHQ production. Aliphatic amides (**1**–**3**) selectively inhibited short-chain AHL biosynthesis (C4-HSL), whereas amides **7** and **9** more strongly affected the quinolone-based QS system. These effects may be attributed to the structural similarity to native autoinducers, allowing the amides to mimic or competitively interfere with QS receptor binding.

The apparent paradox between QS inhibition and biofilm stimulation suggests involvement of alternative regulatory mechanisms. *Pseudomonas aeruginosa* may interpret these volatile amides, likely sensed as environmental or competitive cues, as signals to induce protective biofilm formation. Additionally, their susceptibility to environmental degradation or intracellular metabolism may contribute to their complex biological effects.

Motility assays confirmed that the biofilm-enhancing effects of the tested amides are not attributable to alterations in swarming or twitching motility in *P. aeruginosa* PAO1. In cross-species assays, broader QS-inhibitory activity was not evident; however, *N*-acetyltryptamine (**8**) uniquely inhibited violacein production in *Chromobacterium violaceum* CV026, suggesting a degree of species- and compound-specific QS modulation. Notably, previously documented effects of some of the herein tested amides and structurally related natural products on bacterial motility and pigment biosynthesis have generally been reported at substantially higher concentrations than those used in this study.

Overall, this work provides new insights into the ecophysiological roles of volatile biogenic amides, emphasizing their capacity to be involved in bacterial communication systems. These findings support the emerging view that microbially produced VOCs act as context-dependent signaling molecules that can influence QS and virulence-related behaviors, offering promising leads for the development of novel anti-virulence strategies targeting QS pathways.

## Data Availability

The original contributions presented in this study are included in the article/Supplementary Material. Further inquiries can be directed to the corresponding authors.

## Supplementary Materials

The following supporting information can be downloaded at: https://www.mdpi.com/article/10.3390/molecules31010155/s1, Figure S1: MS spectrum of *N*-(2-methylpropyl)acetamide (**1**); Figure S2: IR spectrum of *N*-(2-methylpropyl)acetamide (**1**); Figure S3: ^1^H NMR spectrum (400 MHz, CDCl_3_) of *N*-(2-methylpropyl)acetamide (**1**); Figure S4: ^13^C NMR spectrum (100.6 MHz, CDCl_3_) of *N*-(2-methylpropyl)acetamide (**1**); Figure S5: MS spectrum of *N*-(2-methylbutyl)acetamide (**2**); Figure S6: IR spectrum of *N*-(2-methylbutyl)acetamide (**2**); Figure S7: ^1^H NMR spectrum (400 MHz, CDCl_3_) of *N*-(2-methylbutyl)acetamide (**2**); Figure S8: ^13^C NMR spectrum (100.6 MHz, CDCl_3_) of *N*-(2-methylbutyl)acetamide (**2**); Figure S9: MS spectrum of *N*-(3-methylbutyl)acetamide (**3**); Figure S10: IR spectrum of *N*-(3-methylbutyl)acetamide (**3**); Figure S11: ^1^H NMR spectrum (400 MHz, CDCl_3_) of *N*-(3-methylbutyl)acetamide (**3**); Figure S12: ^13^C NMR spectrum (100.6 MHz, CDCl_3_) of *N*-(3-methylbutyl)acetamide (**3**); Figure S13: MS spectrum of *N*-(3-methyl-2-butenyl)acetamide (**4**); Figure S14: IR spectrum of *N*-(3-methyl-2-butenyl)acetamide (**4**); Figure S15: ^1^H NMR spectrum (400 MHz, CDCl_3_) of *N*-(3-methyl-2-butenyl)acetamide (**4**); Figure S16: ^13^C NMR spectrum (100.6 MHz, CDCl_3_) of *N*-(3-methyl-2-butenyl)acetamide (**4**); Figure S17: MS spectrum of *N*-benzylacetamide (**5**); Figure S18: IR spectrum of *N*-benzylacetamide (**5**); Figure S19: ^1^H NMR spectrum (400 MHz, CDCl_3_) of *N*-benzylacetamide (**5**); Figure S20: ^13^C NMR spectrum (100.6 MHz, CDCl_3_) of *N*-benzylacetamide (**5**); Figure S21: MS spectrum of *N-*(2-phenylethyl)acetamide (**6**); Figure S22: IR spectrum of *N-*(2-phenylethyl)acetamide (**6**); Figure S23: ^1^H NMR spectrum (400 MHz, CDCl_3_) of *N-*(2-phenylethyl)acetamide (**6**); Figure S24: ^13^C NMR spectrum (100.6 MHz, CDCl_3_) of *N-*(2-phenylethyl)acetamide (**6**); Figure S25: MS spectrum of *N*-acetyltyramine (**7**); Figure S26: IR spectrum of *N*-acetyltyramine (**7**); Figure S27: ^1^H NMR spectrum (400 MHz, DMSO-*d*_6_) of *N*-acetyltyramine (**7**); Figure S28: ^13^C NMR spectrum (100.6 MHz, DMSO-*d*_6_) of *N*-acetyltyramine (**7**); Figure S29: MS spectrum of *N*-acetyltryptamine (**8**); Figure S30: IR spectrum of *N*-acetyltryptamine (**8**); Figure S31: ^1^H NMR spectrum (400 MHz, DMSO-*d*_6_) of *N*-acetyltryptamine (**8**); Figure S32: ^13^C NMR spectrum (100.6 MHz, DMSO-*d*_6_) of *N*-acetyltryptamine (**8**); Figure S33: MS spectrum of 2-phenylacetamide (**9**); Figure S34: IR spectrum of 2-phenylacetamide (**9**); Figure S35: ^1^H NMR spectrum (400 MHz, CDCl_3_) of 2-phenylacetamide (**9**); Figure S36: ^13^C NMR spectrum (100.6 MHz, CDCl_3_) of 2-phenylacetamide (**9**); Figure S37: Gas co-chromatography of synthetic amides **1**–**4** with the whole-culture ethyl acetate extract of *Streptomyces* sp. NP10; Figure S38: Gas co-chromatography of synthetic amides **5**, **6** and **9** with the whole-culture ethyl acetate extract of *Streptomyces* sp. NP10; Figure S39: Gas co-chromatography of synthetic amides **7** and **8** with the whole-culture ethyl acetate extract of *Streptomyces* sp. NP10; Figure S40: A comparison of the experimental (green) and simulated (violet) ^1^H NMR spectra of *N*-(2-methylpropyl)acetamide (**1**); Figure S41: A comparison of the experimental (green) and simulated (violet) ^1^H NMR spectra of *N*-(2-methylbutyl)acetamide (**2**); Figure S42: A comparison of the experimental (green) and simulated (violet) ^1^H NMR spectra of *N*-(3-methylbutyl)acetamide (**3**); Figure S43: A comparison of the experimental (green) and simulated (violet) ^1^H NMR spectra of *N*-(3-methyl-2-butenyl)acetamide (**4**); Figure S44: A comparison of the experimental (green) and simulated (violet) ^1^H NMR spectra of *N*-benzylacetamide (**5**); Figure S45: A comparison of the experimental (green) and simulated (violet) ^1^H NMR spectra of *N-*(2-phenylethyl)acetamide (**6**); Figure S46: A comparison of the experimental (green) and simulated (violet) ^1^H NMR spectra of *N*-acetyltyramine (**7**); Figure S47: A comparison of the experimental (green) and simulated (violet) ^1^H NMR spectra of *N*-acetyltryptamine (**8**); Figure S48: A comparison of the experimental (green) and simulated (violet) ^1^H NMR spectra of 2-phenylacetamide (**9**); Figure S49: The effect of amides **1**–**9** on pyocyanin production in *Pseudomonas aeruginosa* PAO1; Figure S50: Evaluation of DNA binding by amides **1**–**9** using gel electrophoresis; Figure S51: The effects of amides **1**–**9** on the swimming (A), swarming (B), and twitching (C) motilities of *Pseudomonas aeruginosa* PAO1; Figure S52: The effect of amides **1**–**9** on violacein production in *Chromobacterium violaceum* CV026; Figure S53: The effect of amides **1**–**9** on prodigiosin biosynthesis in *Serratia marcescens* ATCC 27117; Table S1: Values of the one-bond coupling constant between the NH proton and ^15^N [^1^*J*(^15^N–H)] and the three-bond coupling constant between ^15^N and the geminal methylene protons [^3^*J*(^15^N–H)] measured for acetamides **1**–**3** and **4**–**8**; Table S2: Overview of amide **1**–**9** concentrations applied in bioassays; ^1^H NMR Full Spin Analysis of Amides **1**–**9**.
