# Supplementary material for: Biogenic Quorum-Sensing Amides from Streptomyces sp. NP10
Source: Molecules. 2026 Jan 1;31(1):155. doi: 10.3390/molecules31010155 (PMC12787655; doi:10.3390/molecules31010155)
Supplement: Supplementary file 1 [file molecules-31-00155-s001.zip › molecules-4048787-supplementary.pdf]

Supplementary Material

for

Biogenic Quorum-Sensing Amides from *Streptomyces* sp. NP10

Marija S. Genčić [1], Tatjana Ilic-Tomic [2,*], Marko Z. Mladenović [1,3,*], Milena Z. Živković Stošić [1], Jasmina Nikodinovic-Runic [2] and Niko S. Radulović [1,*]

[1] Department of Chemistry, Faculty of Sciences and Mathematics, University of Niš, Višegradska 33,
18000 Niš, Serbia; denijum@yahoo.com (M.S.G.); mika.zivkovic1987@gmail.com (M.Z.Ž.S.)
[2] Institute of Molecular Genetics and Genetic Engineering, University of Belgrade, Vojvode Stepe 444a,
11042 Belgrade, Serbia; jasmina.nikodinovic@imgge.bg.ac.rs
[3] Department of Sciences and Mathematics, State University of Novi Pazar, Vuka Karadžića 9,
36300 Novi Pazar, Serbia
* Correspondence: tatjana.ilic-tomic@imgge.bg.ac.rs (T.I.-T.); markohem87@gmail.com (M.Z.M.);
nikoradulovic@yahoo.com (N.S.R.); Tel.: +381-11-397-60-34 (T.I.-T.); +381-18-533-015 (M.Z.M.);
+381-18-533-015 (N.S.R.); Fax: +381-11-397-58-08 (T.I.-T.); +381-18-533-014 (M.Z.M.);
+381-18-533-014 (N.S.R.)

Table of content:

1. **Figure S1.** MS spectrum of *N*-(2-methylpropyl)acetamide (**1**) S4
2. **Figure S2.** IR spectrum of *N*-(2-methylpropyl)acetamide (**1**) S4
3. **Figure S3.** ^{1}H NMR spectrum (400 MHz, $CDCl_3$) of *N*-(2-methylpropyl)acetamide (**1**) S5
4. **Figure S4.** ^{13}C NMR spectrum (100.6 MHz, $CDCl_3$) of *N*-(2-methylpropyl)acetamide (**1**) S5
5. **Figure S5.** MS spectrum of *N*-(2-methylbutyl)acetamide (**2**) S6
6. **Figure S6.** IR spectrum of *N*-(2-methylbutyl)acetamide (**2**) S6
7. **Figure S7.** ^{1}H NMR spectrum (400 MHz, $CDCl_3$) of *N*-(2-methylbutyl)acetamide (**2**) S7
8. **Figure S8.** ^{13}C NMR spectrum (100.6 MHz, $CDCl_3$) of *N*-(2-methylbutyl)acetamide (**2**) S7
9. **Figure S9.** MS spectrum of *N*-(3-methylbutyl)acetamide (**3**) S8
10. **Figure S10.** IR spectrum of *N*-(3-methylbutyl)acetamide (**3**) S8
11. **Figure S11.** ^{1}H NMR spectrum (400 MHz, $CDCl_3$) of *N*-(3-methylbutyl)acetamide (**3**) S9
12. **Figure S12.** ^{13}C NMR spectrum (100.6 MHz, $CDCl_3$) of *N*-(3-methylbutyl)acetamide (**3**) S9
13. **Figure S13.** MS spectrum of *N*-(3-methyl-2-butenyl)acetamide (**4**) S10
14. **Figure S14.** IR spectrum of *N*-(3-methyl-2-butenyl)acetamide (**4**) S10
15. **Figure S15.** ^{1}H NMR spectrum (400 MHz, $CDCl_3$) of *N*-(3-methyl-2-butenyl)acetamide (**4**) S11
16. **Figure S16.** ^{13}C NMR spectrum (100.6 MHz, $CDCl_3$) of *N*-(3-methyl-2-butenyl)acetamide (**4**) S11
17. **Figure S17.** MS spectrum of *N*-benzylacetamide (**5**) S12

18. **Figure S18.** IR spectrum of *N*-benzylacetamide (**5**) S12

19. **Figure S19.** ^{1}H NMR spectrum (400 MHz, $CDCl_3$) of *N*-benzylacetamide (**5**) S13

20. **Figure S20.** ^{13}C NMR spectrum (100.6 MHz, $CDCl_3$) of *N*-benzylacetamide (**5**) S13

21. **Figure S21.** MS spectrum of *N*-(2-phenylethyl)acetamide (**6**) S14

22. **Figure S22.** IR spectrum of *N*-(2-phenylethyl)acetamide (**6**) S14

23. **Figure S23.** ^{1}H NMR spectrum (400 MHz, $CDCl_3$) of *N*-(2-phenylethyl)acetamide (**6**) S15

24. **Figure S24.** ^{13}C NMR spectrum (100.6 MHz, $CDCl_3$) of *N*-(2-phenylethyl)acetamide (**6**) S15

25. **Figure S25.** MS spectrum of *N*-acetyltyramine (**7**) S16

26. **Figure S26.** IR spectrum of *N*-acetyltyramine (**7**) S16

27. **Figure S27.** ^{1}H NMR spectrum (400 MHz, DMSO-d_6) of *N*-acetyltyramine (**7**) S17

28. **Figure S28.** ^{13}C NMR spectrum (100.6 MHz, DMSO-d_6) of *N*-acetyltyramine (**7**) S17

29. **Figure S29.** MS spectrum of *N*-acetyltryptamine (**8**) S18

30. **Figure S30.** IR spectrum of *N*-acetyltryptamine (**8**) S18

31. **Figure S31.** ^{1}H NMR spectrum (400 MHz, DMSO-d_6) of *N*-acetyltryptamine (**8**) S19

32. **Figure S32.** ^{13}C NMR spectrum (100.6 MHz, DMSO-d_6) of *N*-acetyltryptamine (**8**) S19

33. **Figure S33.** MS spectrum of 2-phenylacetamide (**9**) S20

34. **Figure S34.** IR spectrum of 2-phenylacetamide (**9**) S20

35. **Figure S35.** ^{1}H NMR spectrum (400 MHz, $CDCl_3$) of 2-phenylacetamide (**9**) S21

36. **Figure S36.** ^{13}C NMR spectrum (100.6 MHz, $CDCl_3$) of 2-phenylacetamide (**9**) S21

37. **Figure S37.** Gas co-chromatography of synthetic amides **1-4** with the whole-culture ethyl acetate extract of *Streptomyces* sp. NP10 S22

38. **Figure S38.** Gas co-chromatography of synthetic amides **5**, **6** and **9** with the whole-culture ethyl acetate extract of *Streptomyces* sp. NP10 S22

39. **Figure S39.** Gas co-chromatography of synthetic amides **7** and **8** with the whole-culture ethyl acetate extract of *Streptomyces* sp. NP10 S23

40. **Figure S40.** A comparison of the experimental (green) and simulated (violet) ^{1}H NMR spectra of *N*-(2-methylpropyl)acetamide (**1**) S23

41. **Figure S41.** A comparison of the experimental (green) and simulated (violet) ^{1}H NMR spectra of *N*-(2-methylbutyl)acetamide (**2**) S24

42. **Figure S42.** A comparison of the experimental (green) and simulated (violet) ^{1}H NMR spectra of *N*-(3-methylbutyl)acetamide (**3**) S24

43. **Figure S43.** A comparison of the experimental (green) and simulated (violet) ^{1}H NMR spectra of *N*-(3-methyl-2-butenyl)acetamide (**4**) S25

44. **Figure S44.** A comparison of the experimental (green) and simulated (violet) ^{1}H NMR spectra of *N*-benzylacetamide (**5**) S25

45. **Figure S45.** A comparison of the experimental (green) and simulated (violet) ^{1}H NMR spectra of *N*-(2-phenylethyl)acetamide (**6**) S26

46. **Figure S46.** A comparison of the experimental (green) and simulated (violet) ^{1}H NMR spectra of *N*-acetyltyramine (**7**) S26

47. **Figure S47.** A comparison of the experimental (green) and simulated (violet) ^{1}H NMR spectra of S27

N-acetyltryptamine (**8**)
48. **Figure S48.** A comparison of the experimental (green) and simulated (violet) ^{1}H NMR spectra of 2-phenylacetamide (**9**) S27
49. **Figure S49.** The effect of amides **1-9** on pyocyanin production in *Pseudomonas aeruginosa* PAO1 S28
50. **Figure S50.** Evaluation of DNA binding by amides **1-9** using gel electrophoresis S28
51. **Figure S51.** The effects of amides **1-9** on the swimming (A), swarming (B), and twitching (C) motilities of *Pseudomonas aeruginosa* PAO1 S29
52. **Figure S52.** The effect of amides **1-9** on violacein production in *Chromobacterium violaceum* CV026 S29
53. **Figure S53.** The effect of amides **1-9** on prodigiosin biosynthesis in *Serratia marcescens* ATCC 27117 S30
54. **Table S1** Values of the one-bond coupling constant between the NH proton and ^{15}N [$^{1}J(^{15}$N–H)] and the three-bond coupling constant between ^{15}N and the geminal methylene protons [$^{3}J(^{15}$N–H)] measured for acetamides **1-3** and **4-8** S30
55. **Table S2** Overview of amide **1-9** concentrations applied in bioassays S31
56. ^{1}H NMR Full Spin Analysis of Amides **1-9** S32
57. **References** S34

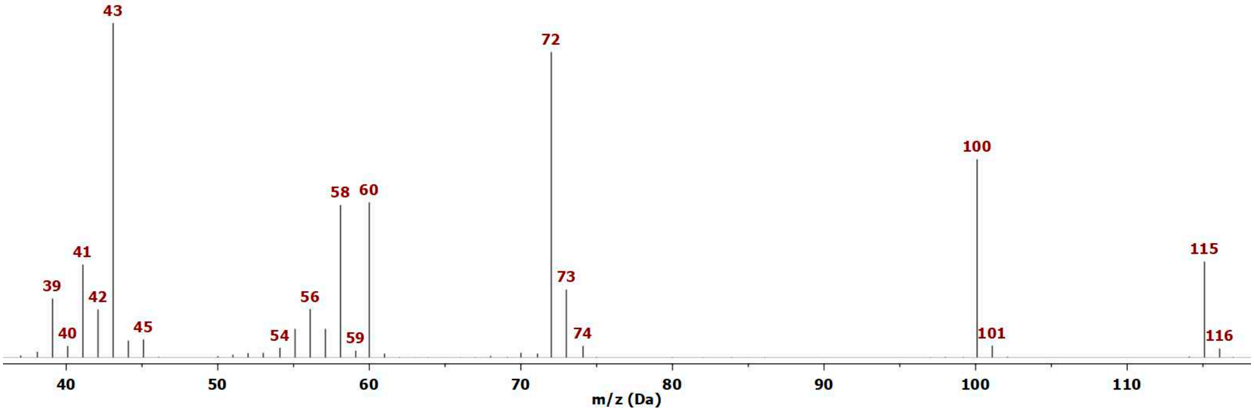

Figure S1. MS spectrum of *N*-(2-methylpropyl)acetamide (**1**)

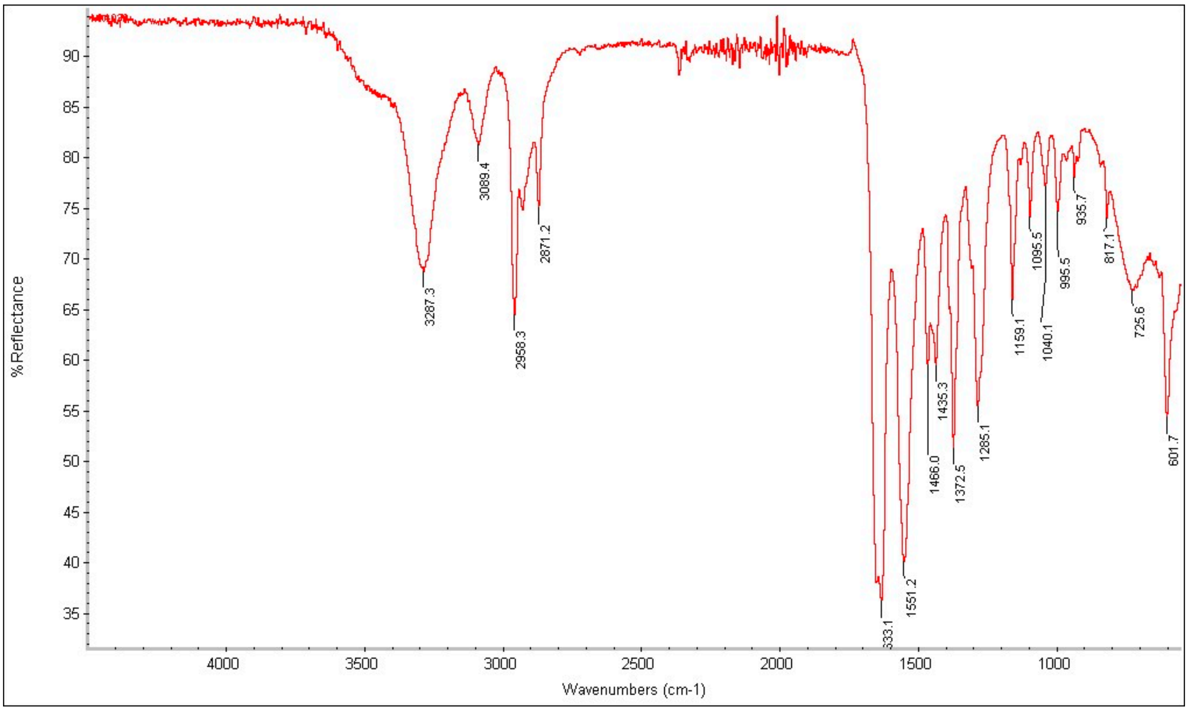

Figure S2. IR spectrum of *N*-(2-methylpropyl)acetamide (**1**)

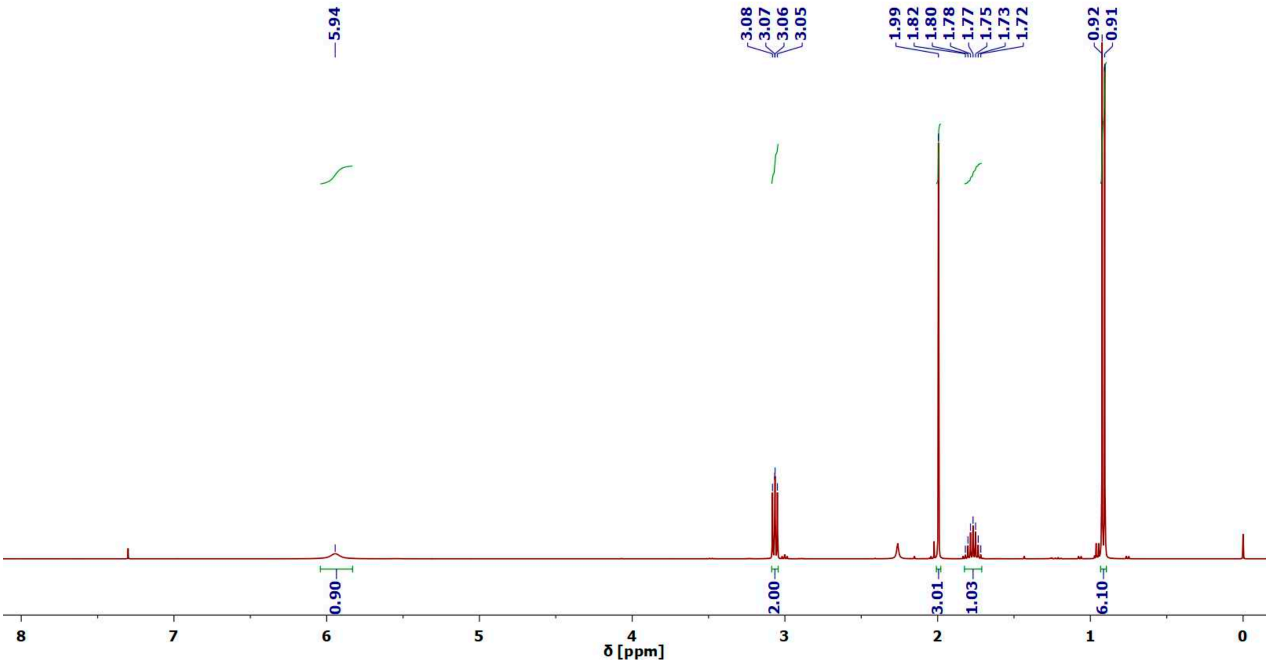

Figure S3. ^{1}H NMR spectrum (400 MHz, $CDCl_3$) of *N*-(2-methylpropyl)acetamide (**1**)

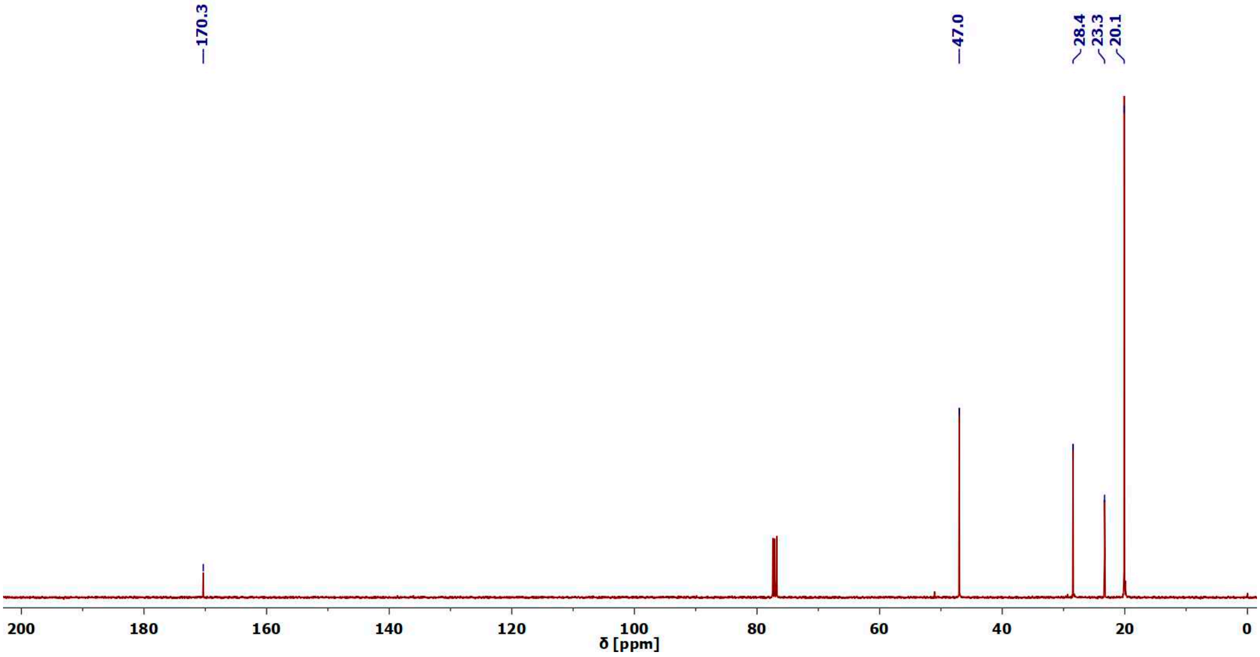

Figure S4. ^{13}C NMR spectrum (100.6 MHz, $CDCl_3$) of *N*-(2-methylpropyl)acetamide (**1**)

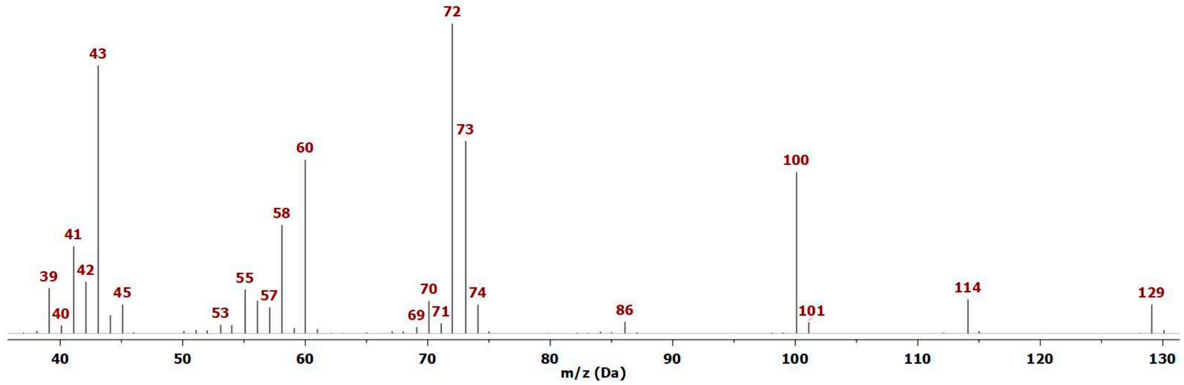

Figure S5. MS spectrum of *N*-(2-methylbutyl)acetamide (**2**)

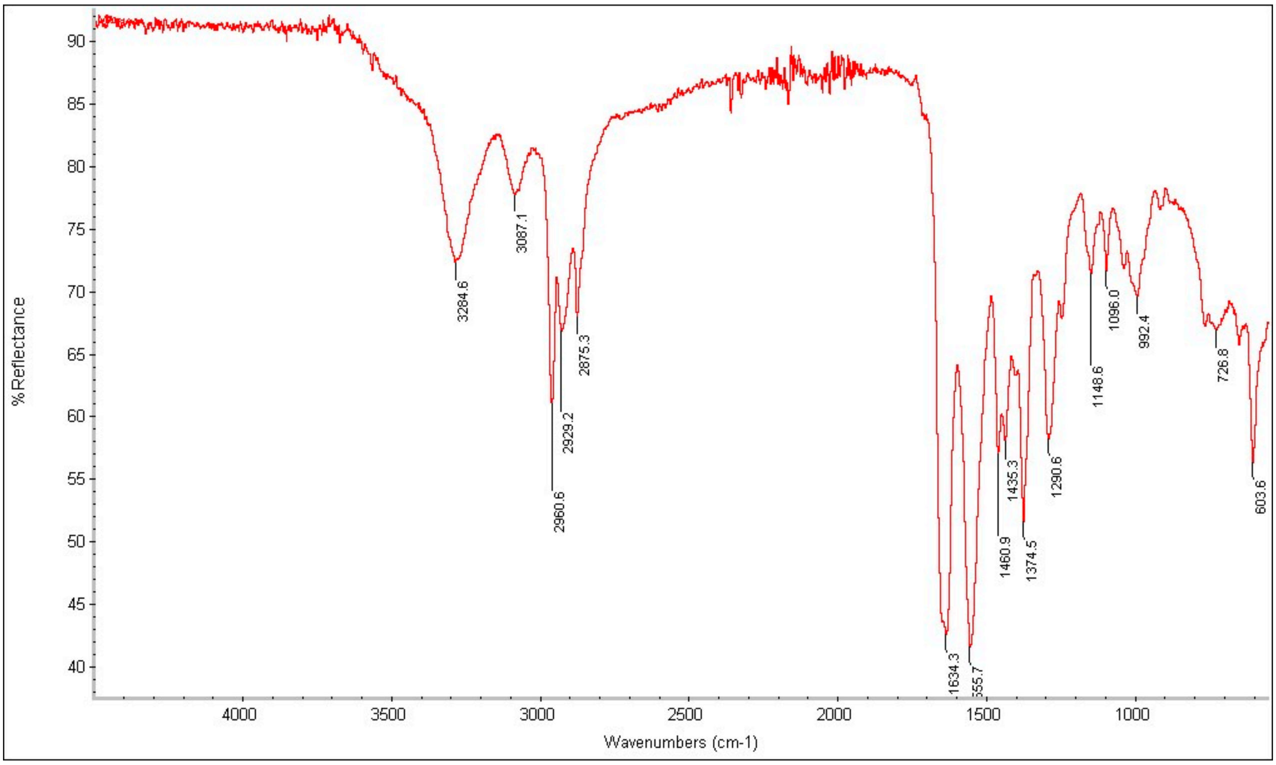

Figure S6. IR spectrum of *N*-(2-methylbutyl)acetamide (**2**)

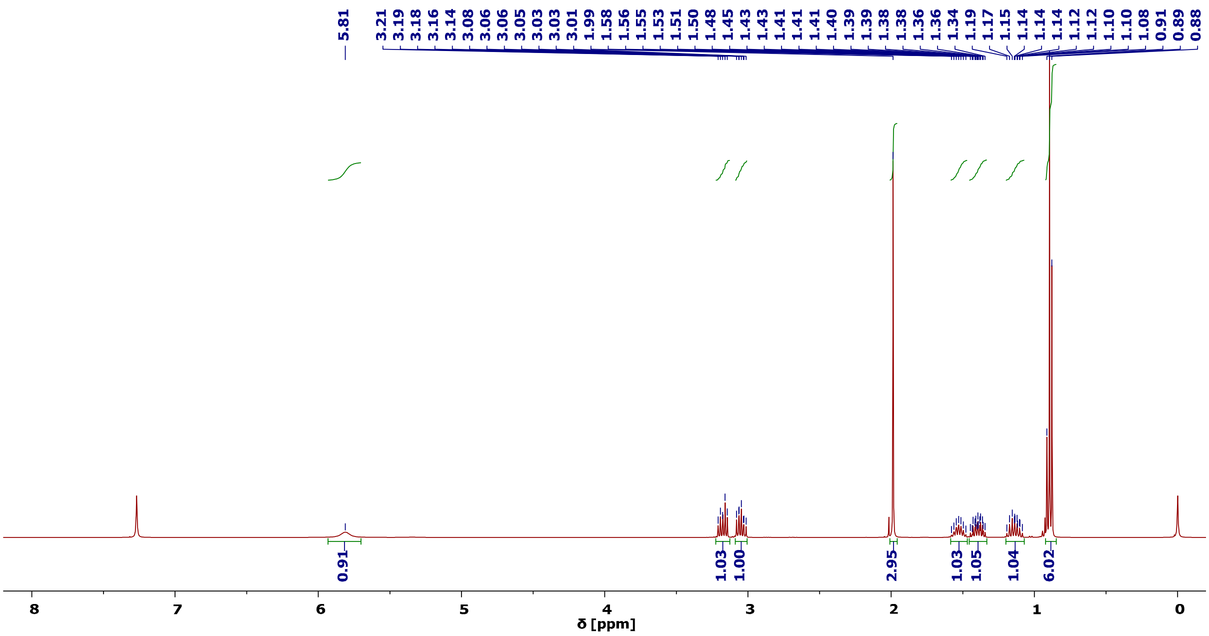

Figure S7. ^{1}H NMR spectrum (400 MHz, $CDCl_3$) of *N*-(2-methylbutyl)acetamide (**2**)

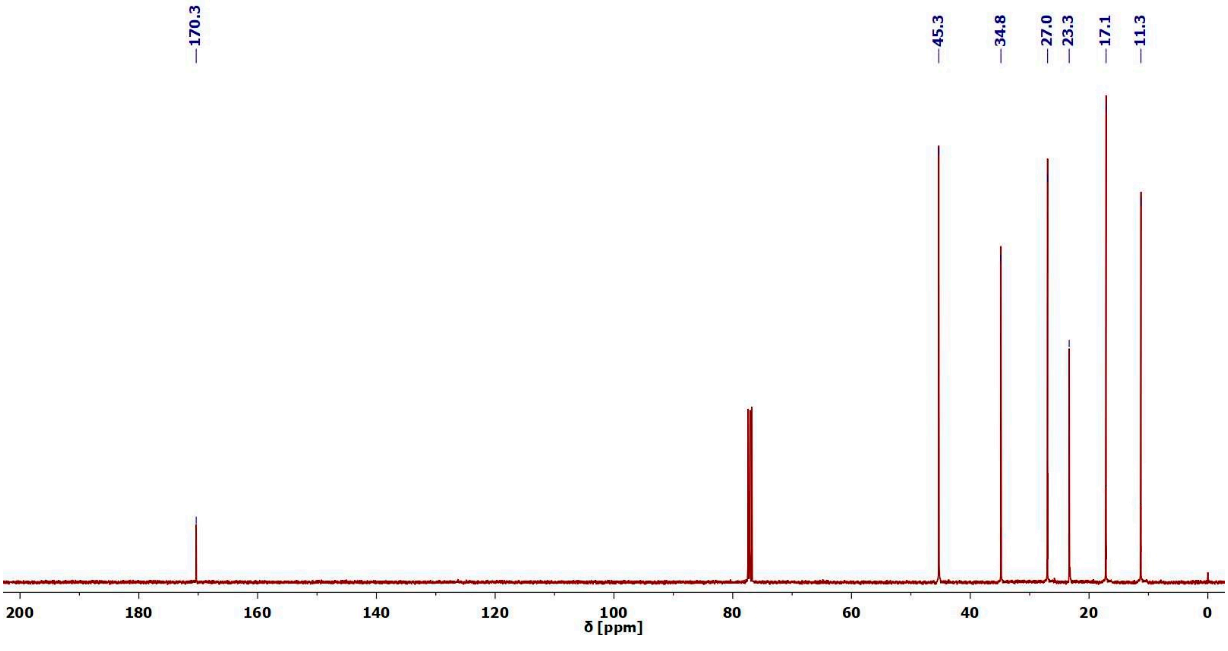

Figure S8. ^{13}C NMR spectrum (100.6 MHz, $CDCl_3$) of *N*-(2-methylbutyl)acetamide (**2**)

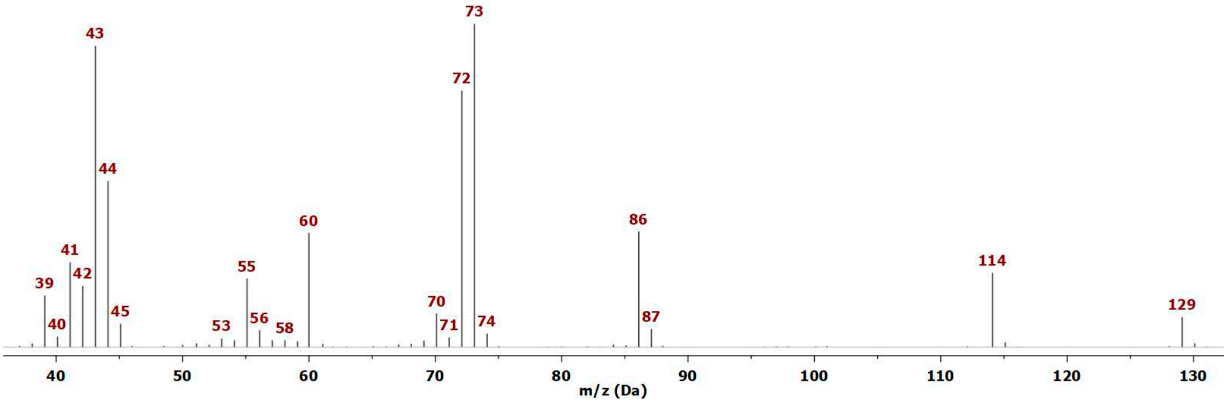

Figure S9. MS spectrum of *N*-(3-methylbutyl)acetamide (**3**)

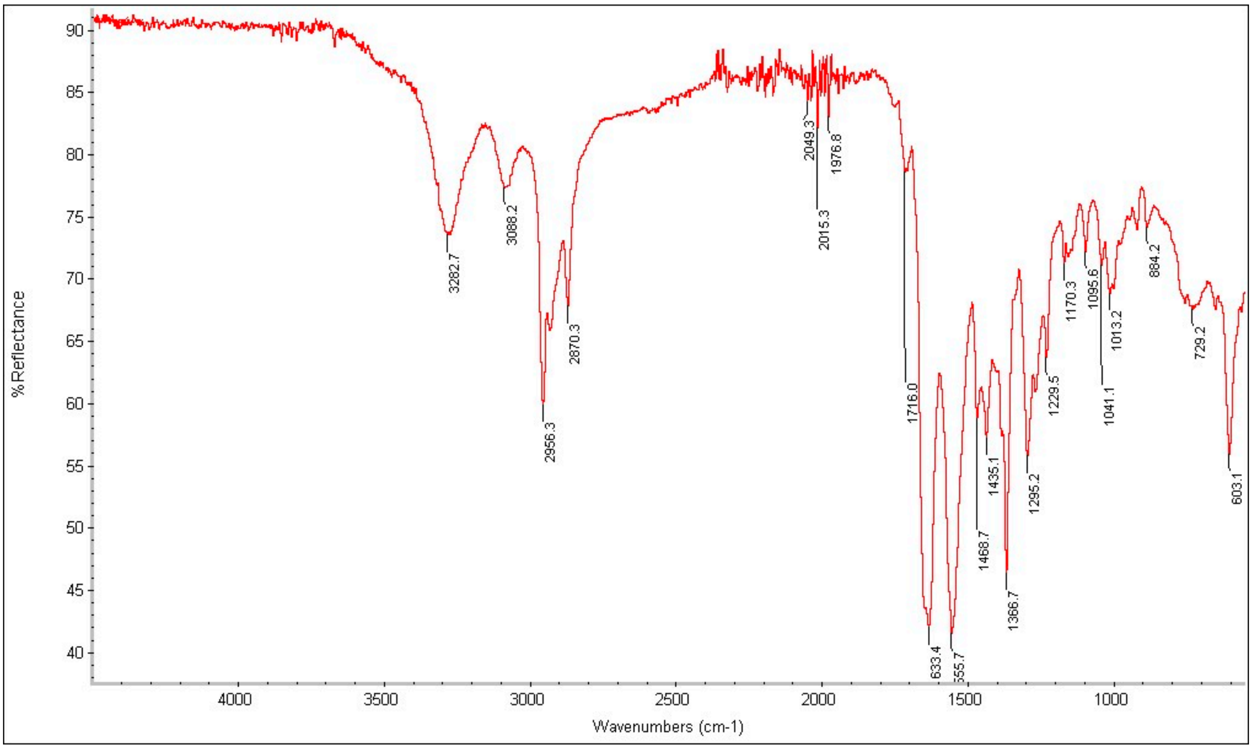

Figure S10. IR spectrum of *N*-(3-methylbutyl)acetamide (**3**)

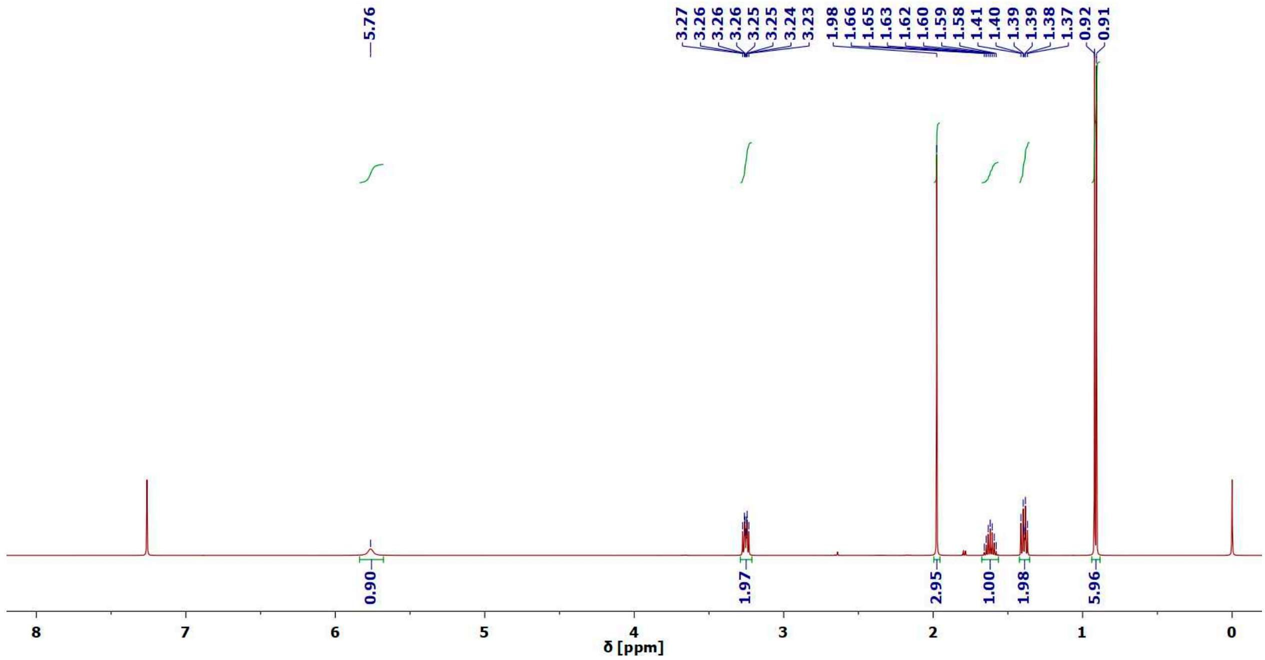

Figure S11. ^{1}H NMR spectrum (400 MHz, $CDCl_3$) of *N*-(3-methylbutyl)acetamide (**3**)

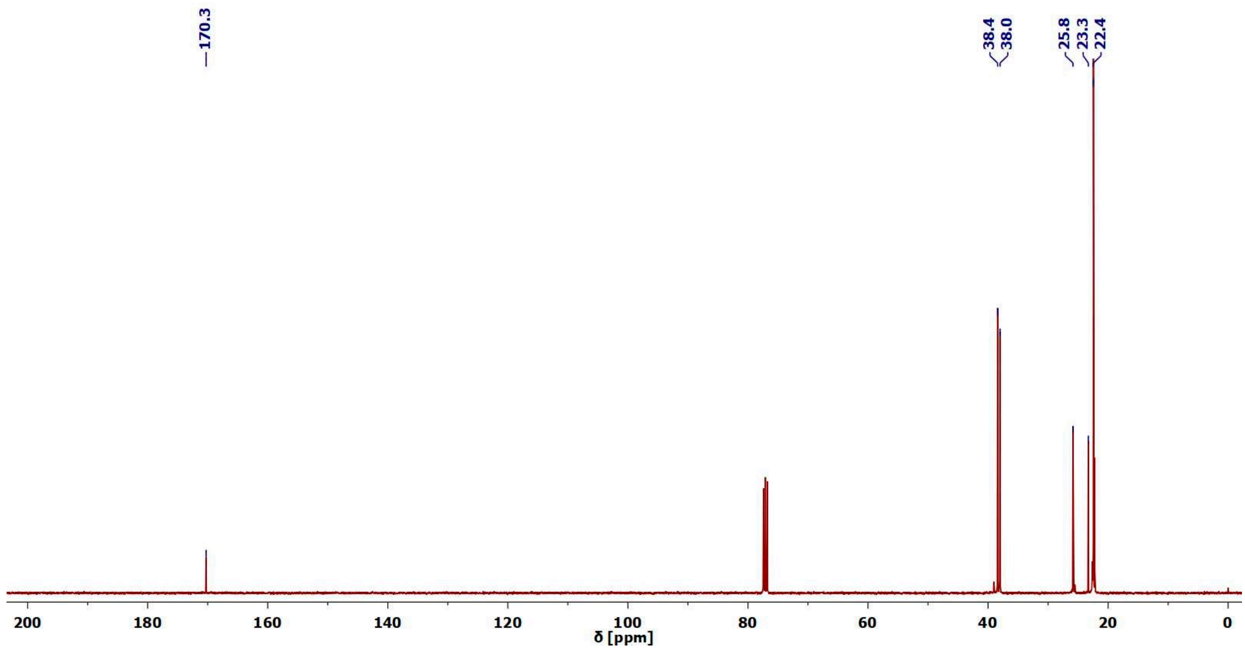

Figure S12. ^{13}C NMR spectrum (100.6 MHz, $CDCl_3$) of *N*-(3-methylbutyl)acetamide (**3**)

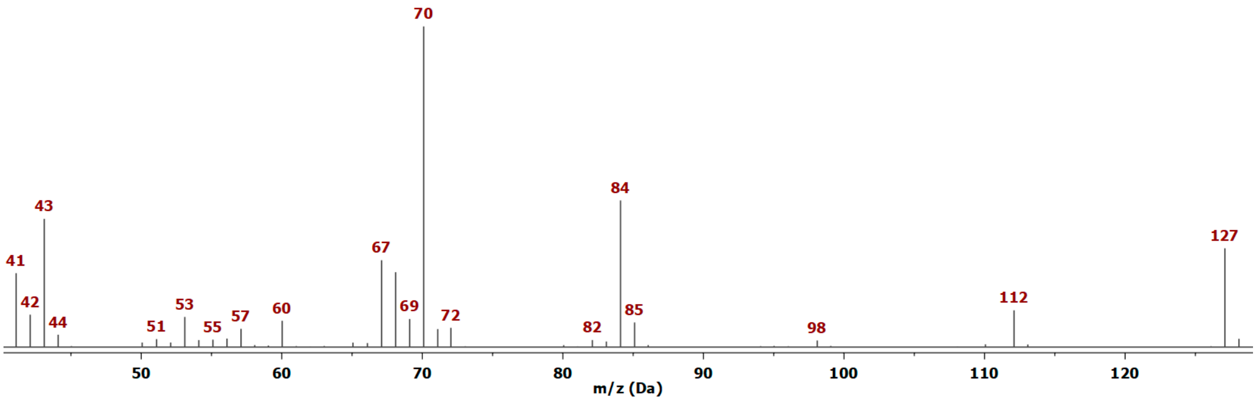

Figure S13. MS spectrum of *N*-(3-methyl-2-butenyl)acetamide (**4**)

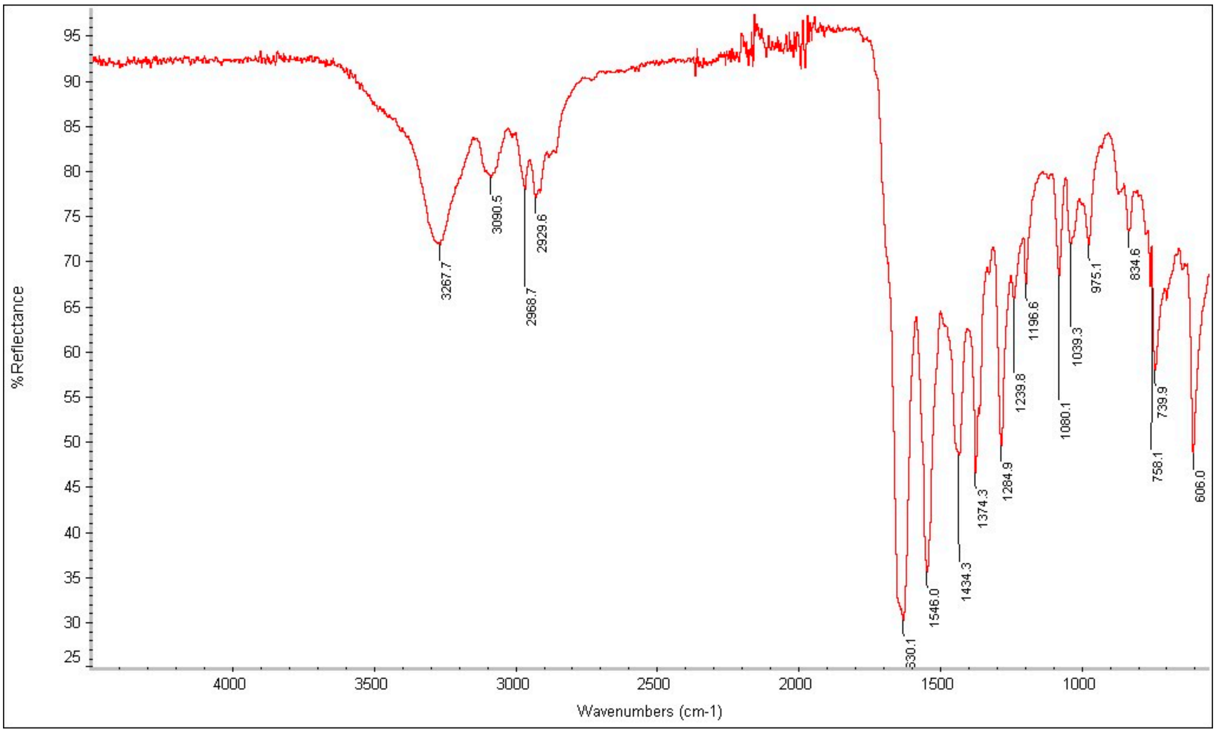

Figure S14. IR spectrum of *N*-(3-methyl-2-butenyl)acetamide (**4**)

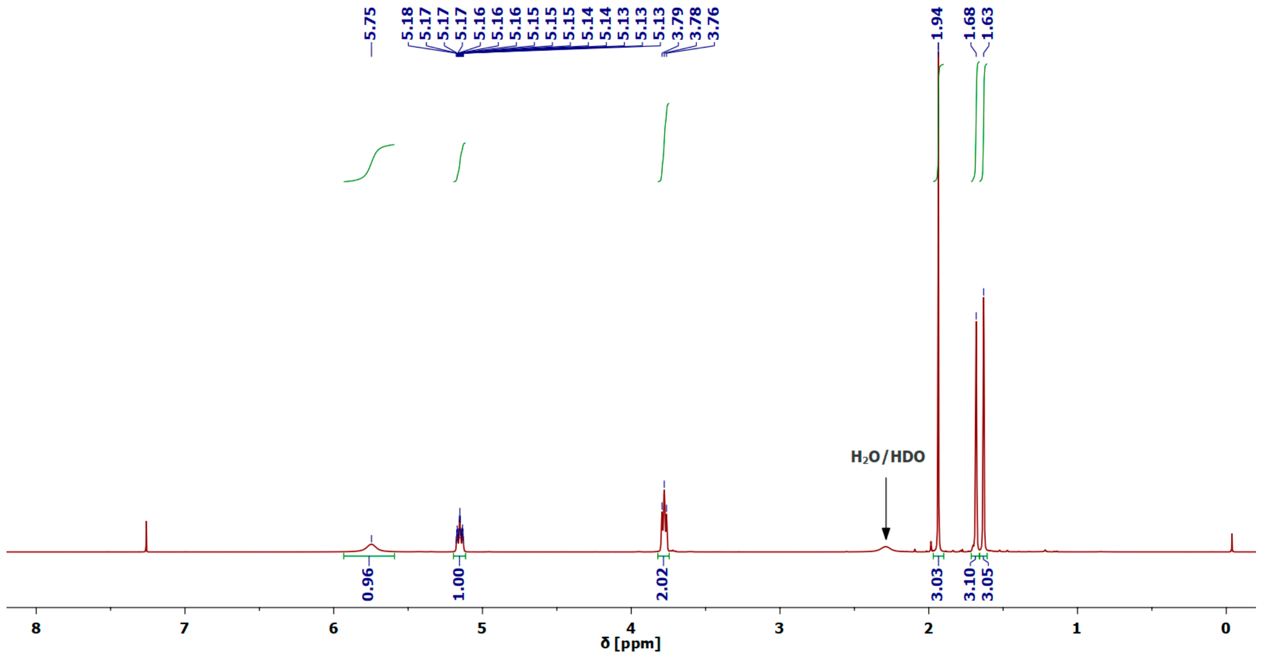

Figure S15. ^{1}H NMR spectrum (400 MHz, $CDCl_3$) of *N*-(3-methyl-2-butenyl)acetamide (**4**)

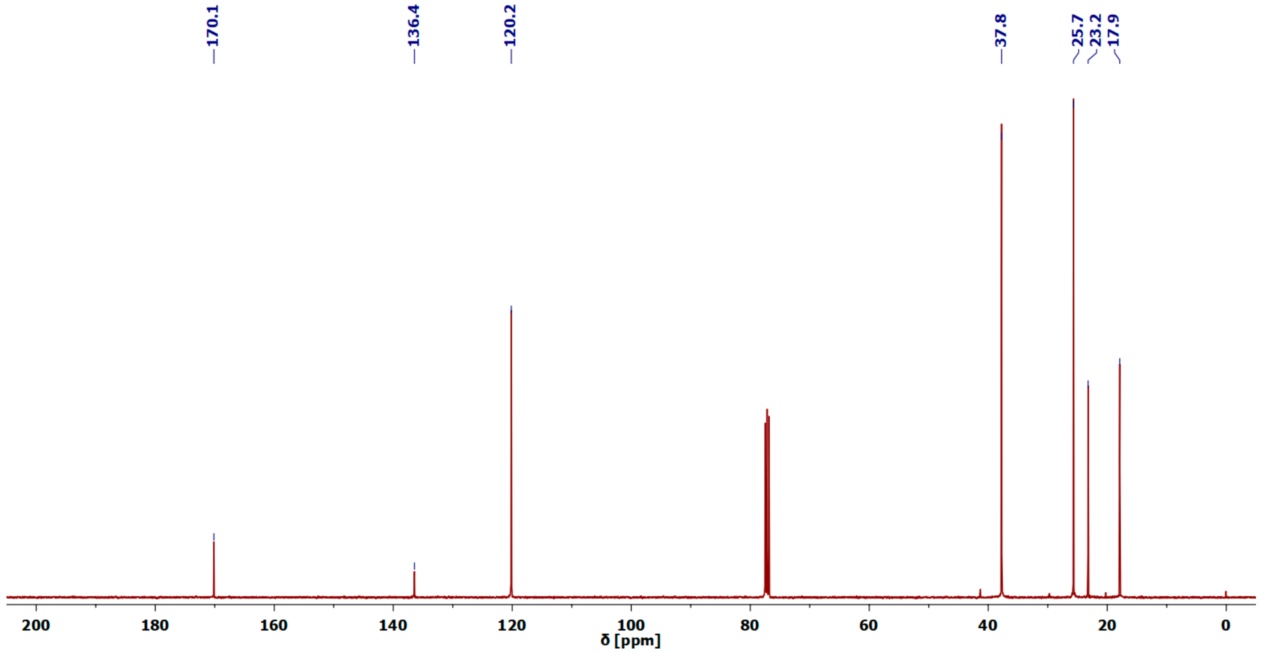

Figure S16. ^{13}C NMR spectrum (100.6 MHz, $CDCl_3$) of *N*-(3-methyl-2-butenyl)acetamide (**4**)

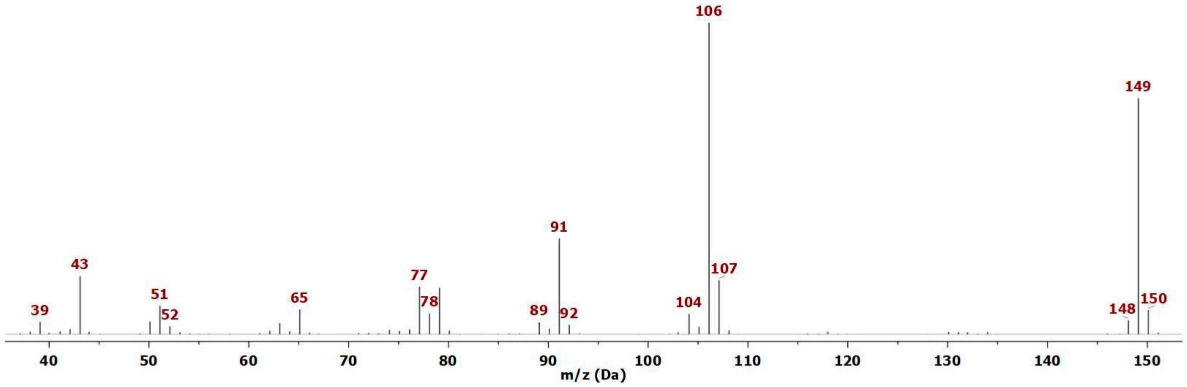

Figure S17. MS spectrum of *N*-benzylacetamide (**5**)

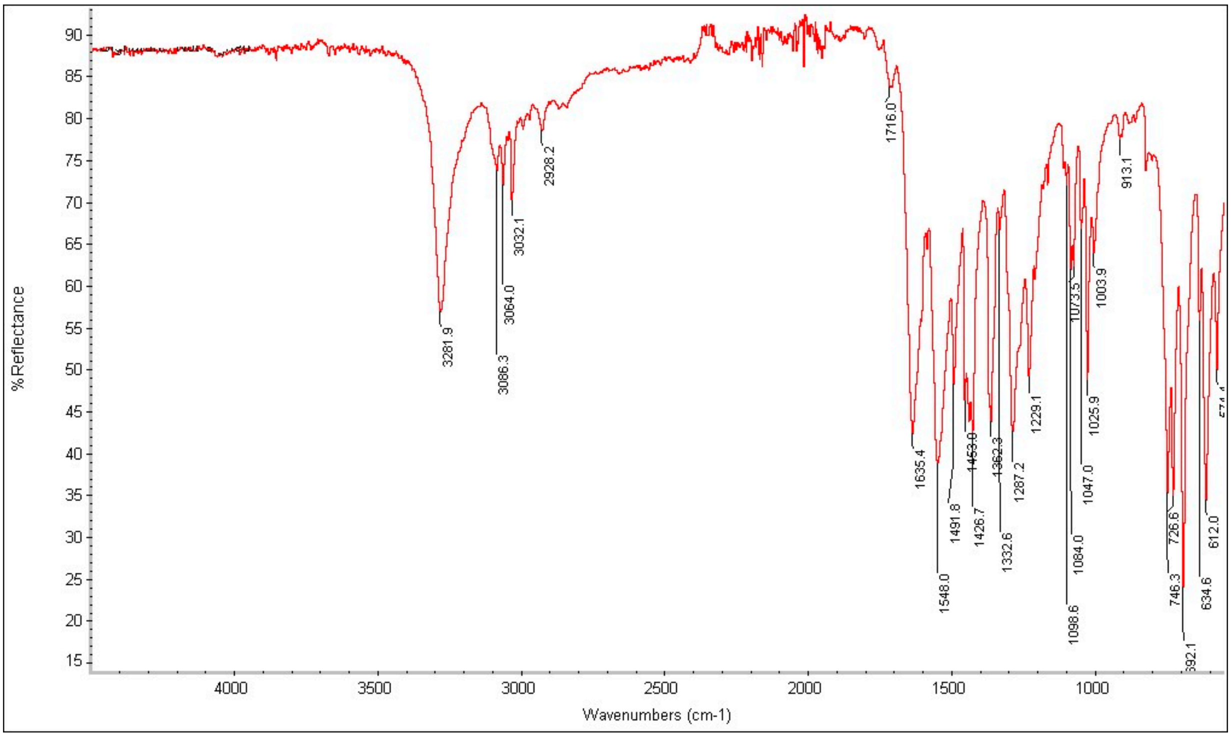

Figure S18. IR spectrum of *N*-benzylacetamide (**5**)

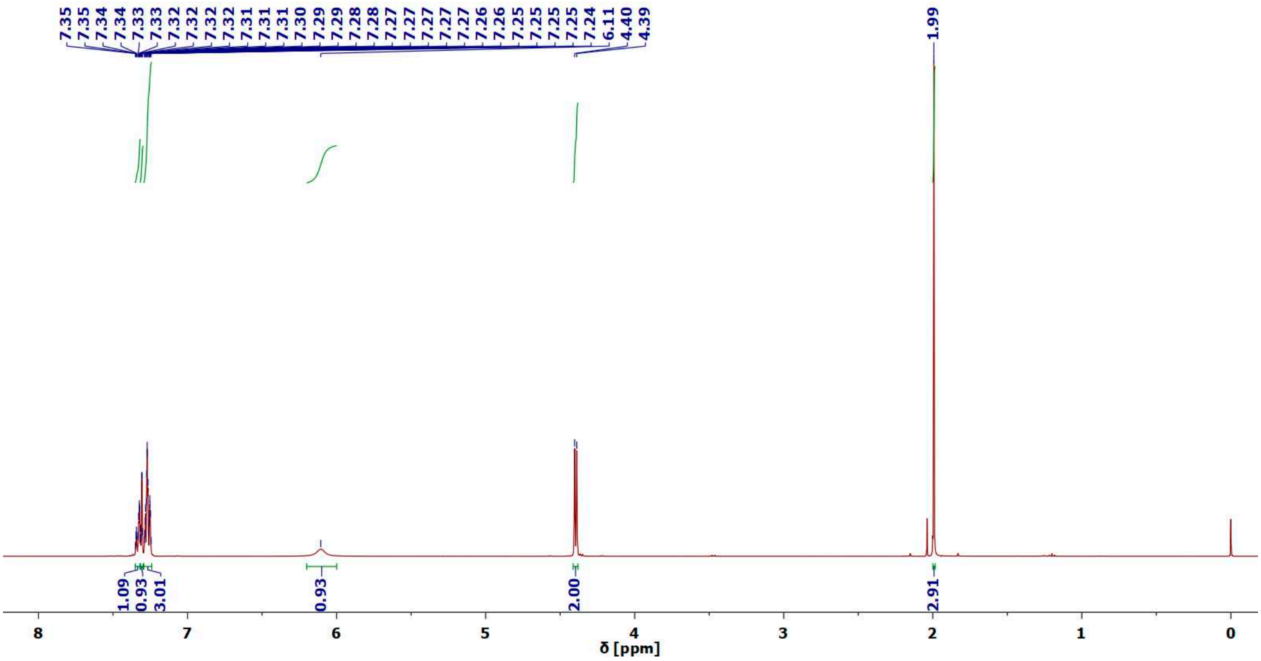

Figure S19. ^{1}H NMR spectrum (400 MHz, $CDCl_3$) of *N*-benzylacetamide (**5**)

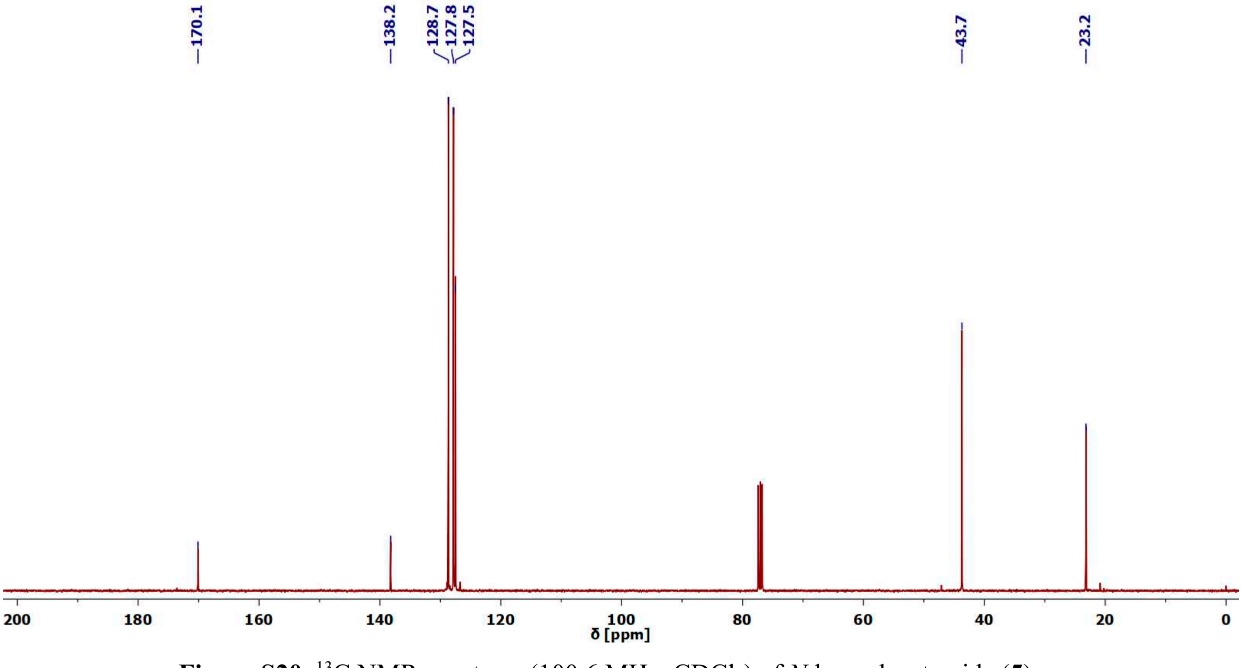

Figure S20. ^{13}C NMR spectrum (100.6 MHz, $CDCl_3$) of *N*-benzylacetamide (**5**)

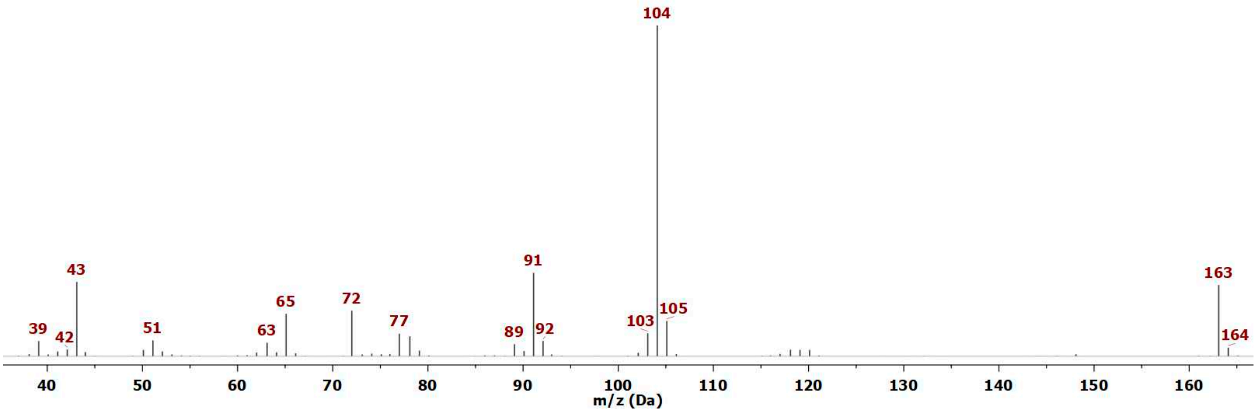

Figure S21. MS spectrum of *N*-(2-phenylethyl)acetamide (**6**)

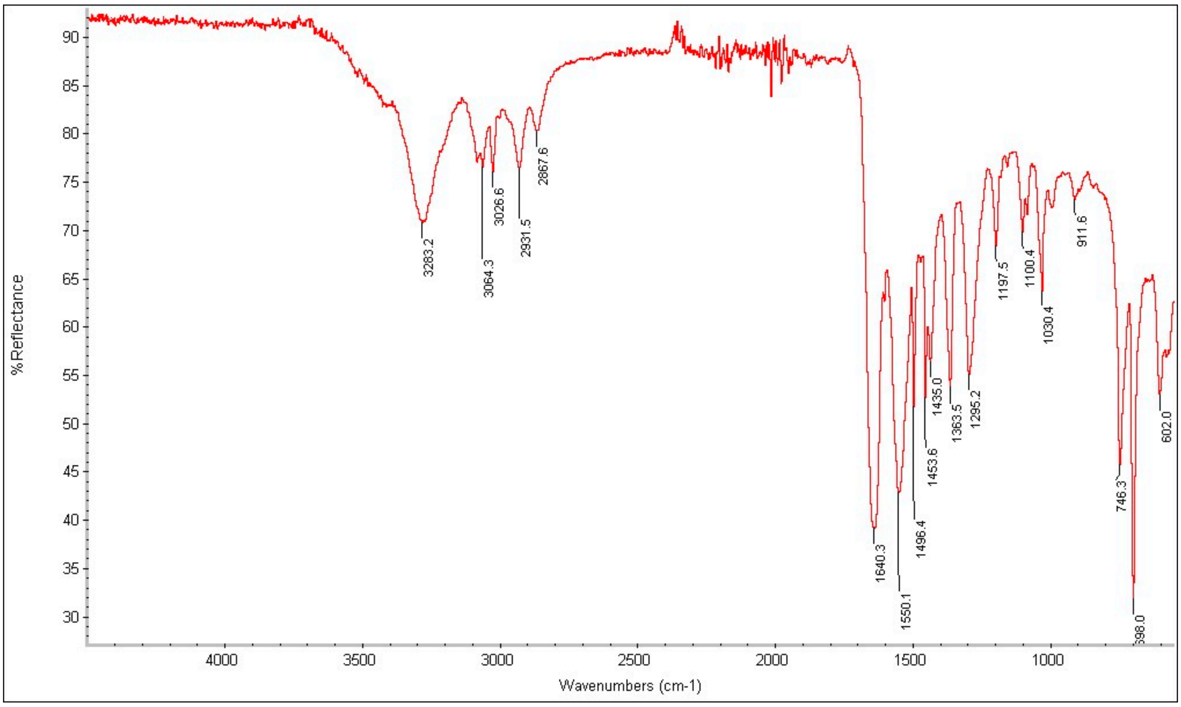

Figure S22. IR spectrum of *N*-(2-phenylethyl)acetamide (**6**)

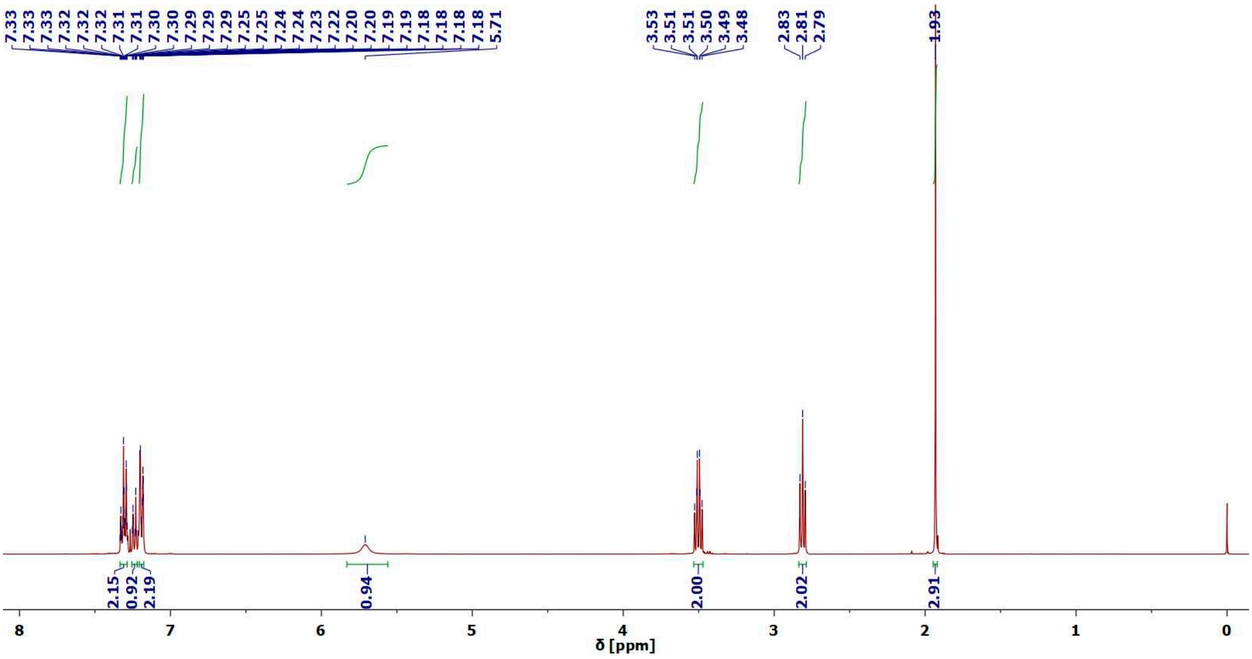

Figure S23. ^{1}H NMR spectrum (400 MHz, $CDCl_3$) of *N*-(2-phenylethyl)acetamide (**6**)

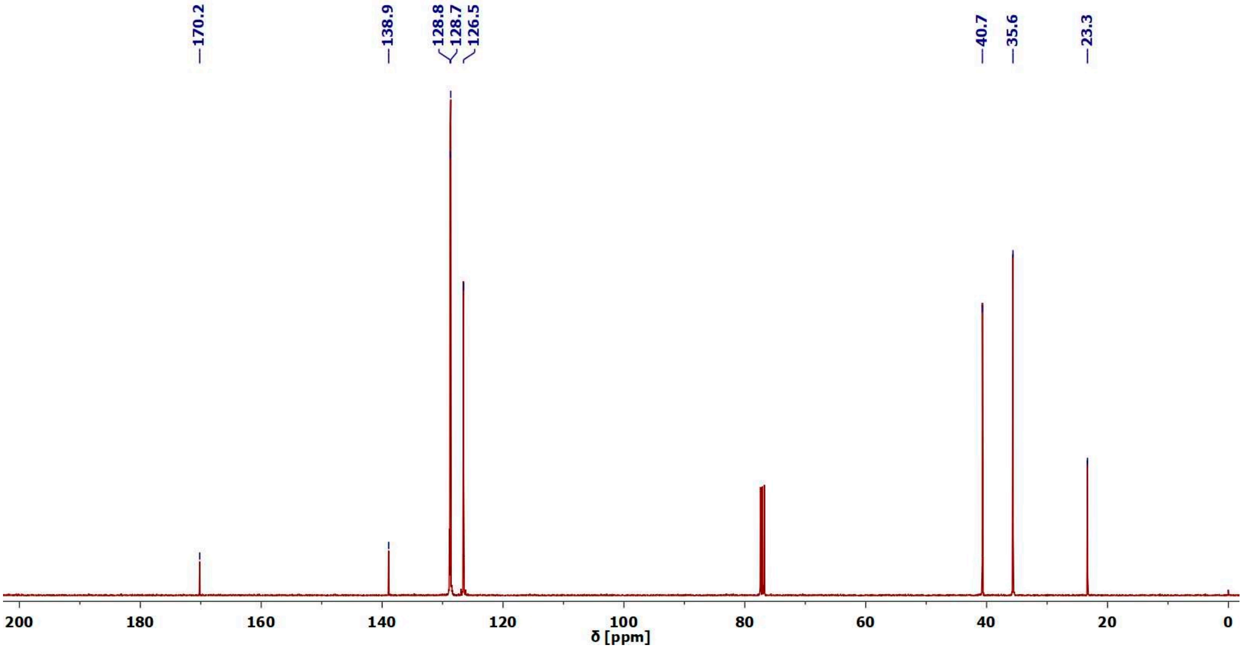

Figure S24. ^{13}C NMR spectrum (100.6 MHz, $CDCl_3$) of *N*-(2-phenylethyl)acetamide (**6**)

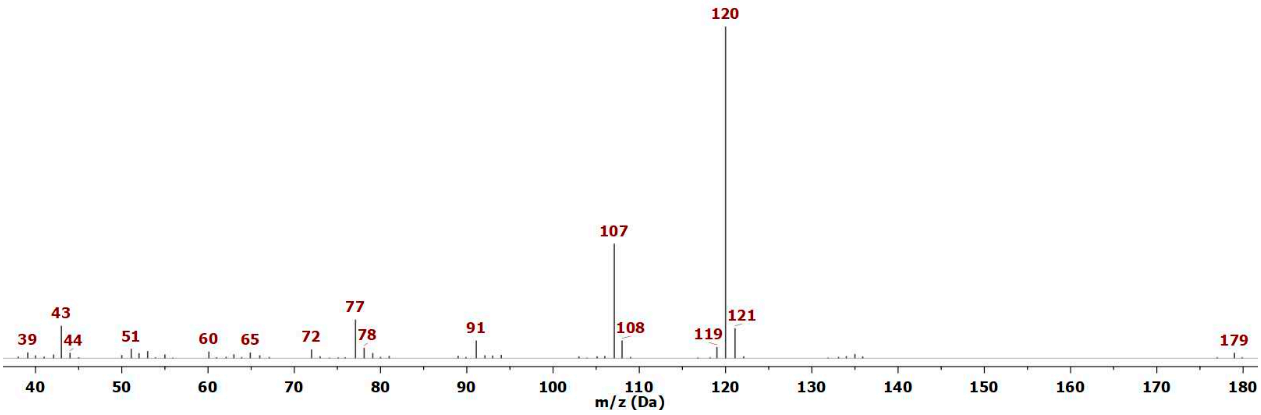

Figure S25. MS spectrum of *N*-acetyltyramine (**7**)

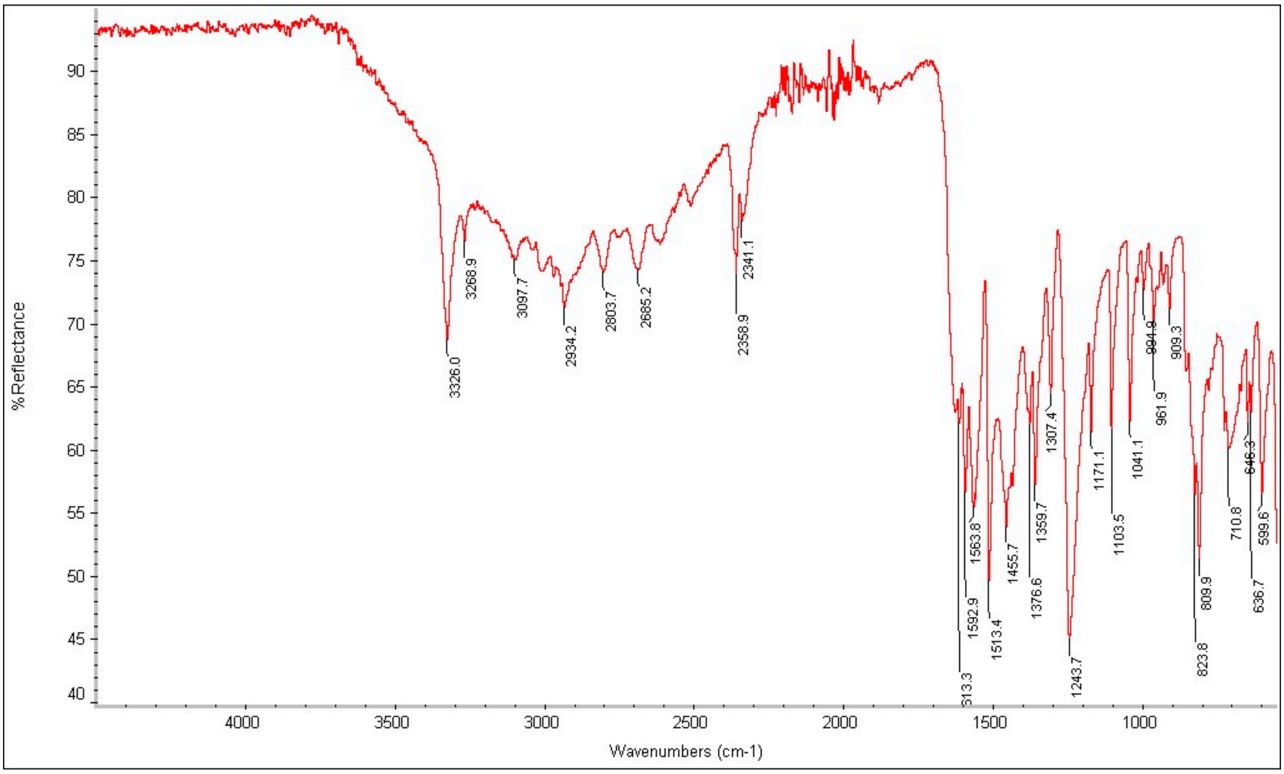

Figure S26. IR spectrum of *N*-acetyltyramine (**7**)

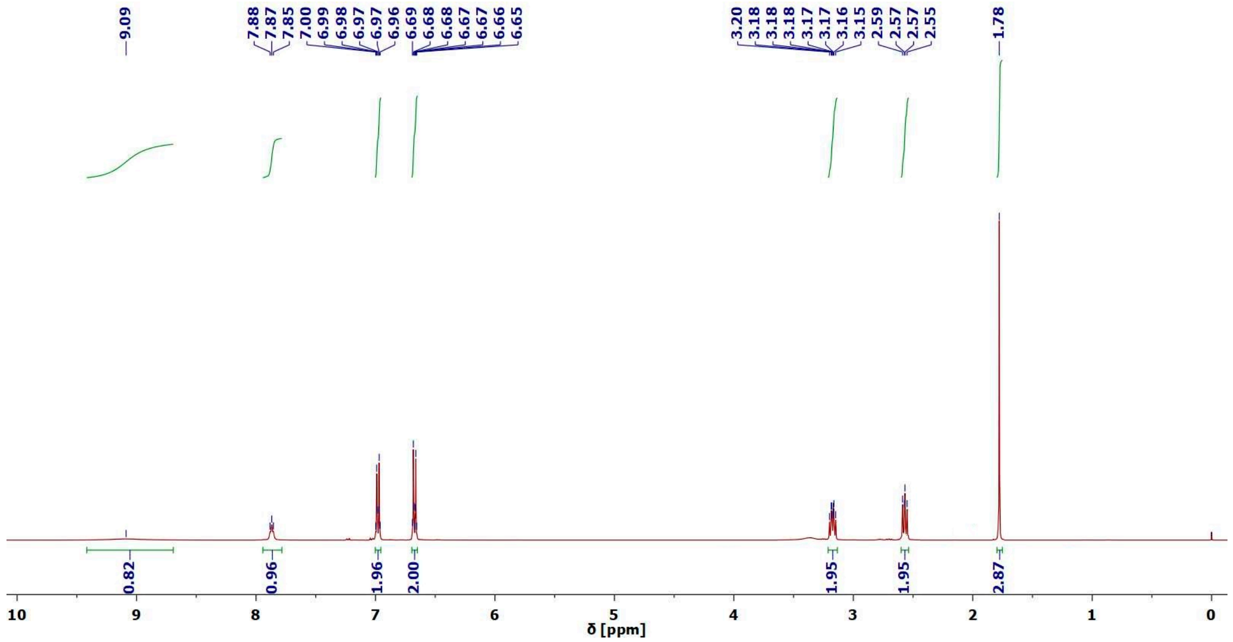

Figure S27. ^{1}H NMR spectrum (400 MHz, DMSO-d_6) of *N*-acetyltyramine (**7**)

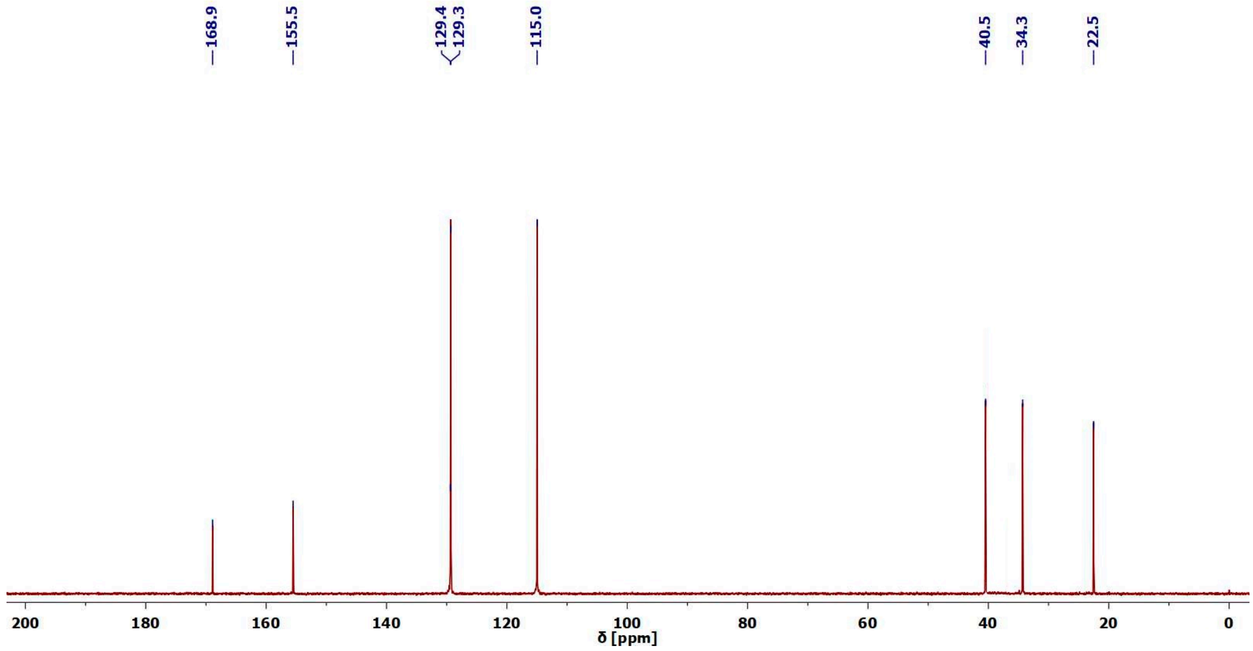

Figure S28. ^{13}C NMR spectrum (100.6 MHz, DMSO-d_6) of *N*-acetyltyramine (**7**)

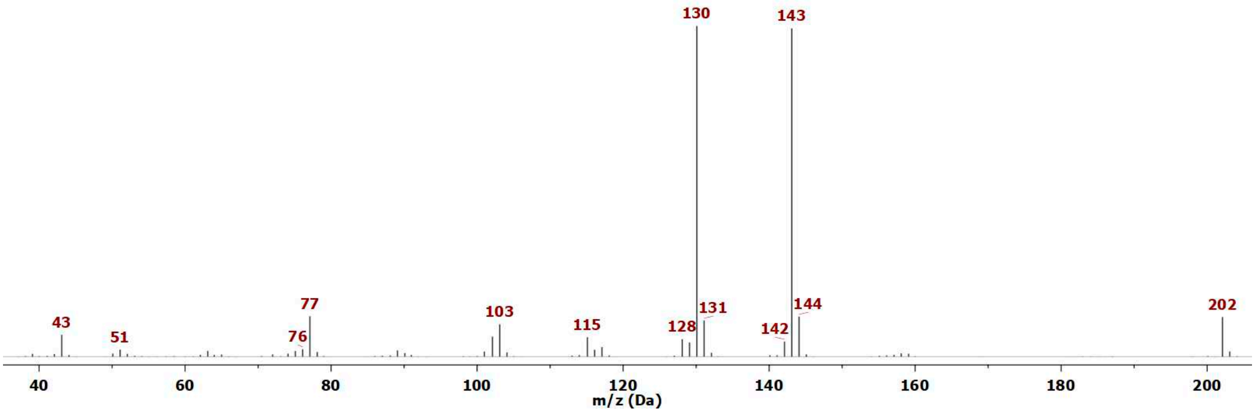

Figure S29. MS spectrum of *N*-acetyltryptamine (**8**)

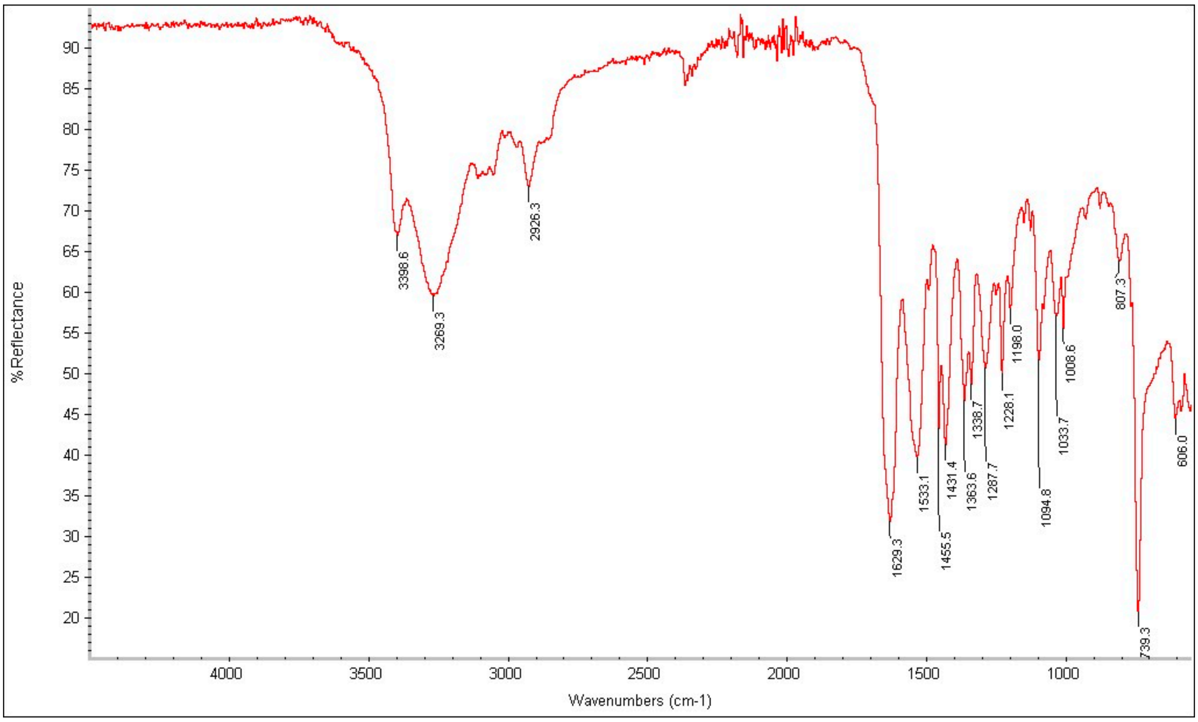

Figure S30. IR spectrum of *N*-acetyltryptamine (**8**)

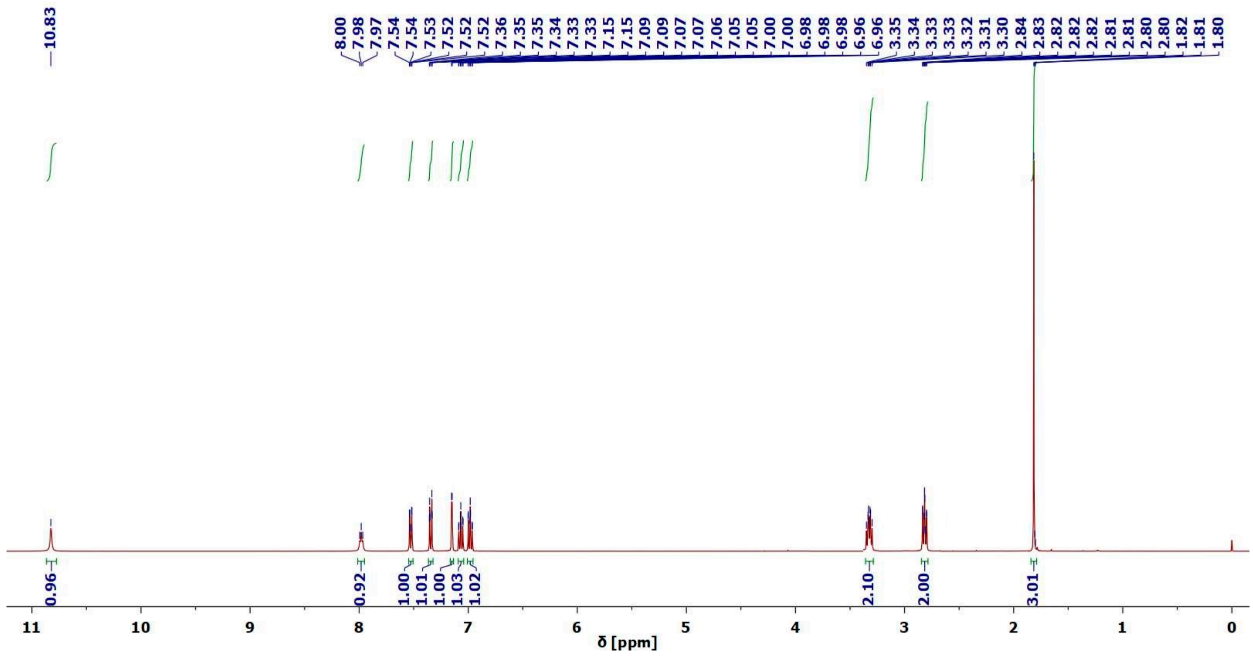

Figure S31. ^{1}H NMR spectrum (400 MHz, DMSO-d_6) of *N*-acetyltryptamine (**8**)

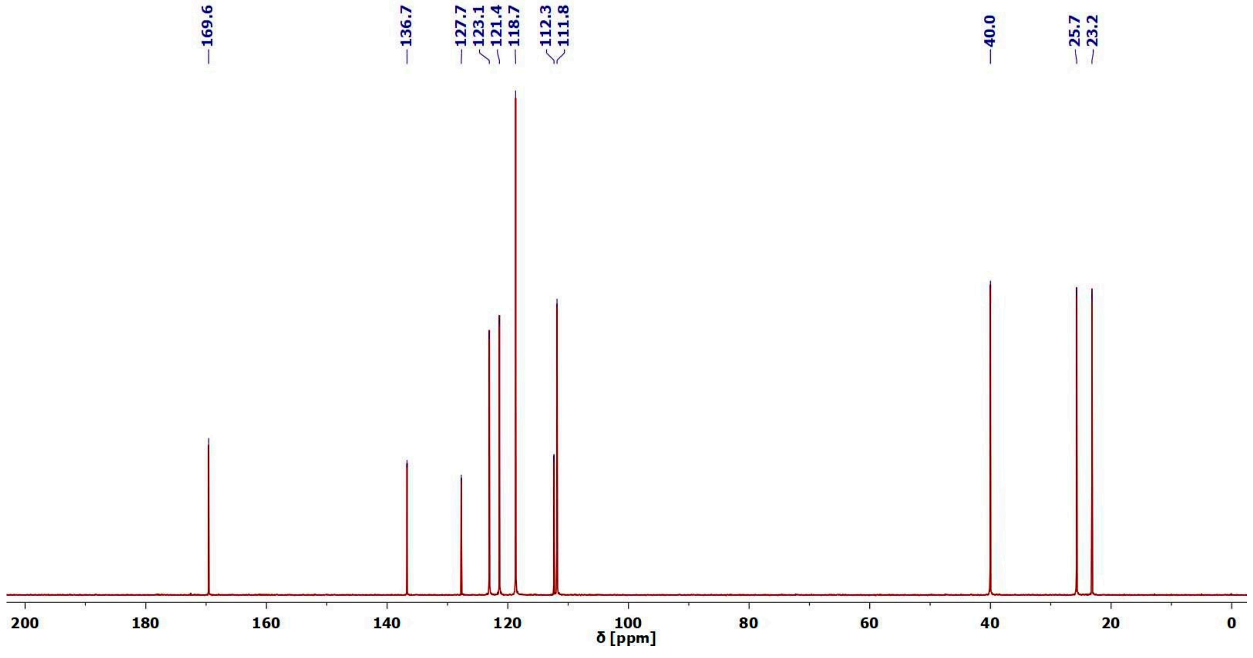

Figure S32. ^{13}C NMR spectrum (100.6 MHz, DMSO-d_6) of *N*-acetyltryptamine (**8**)

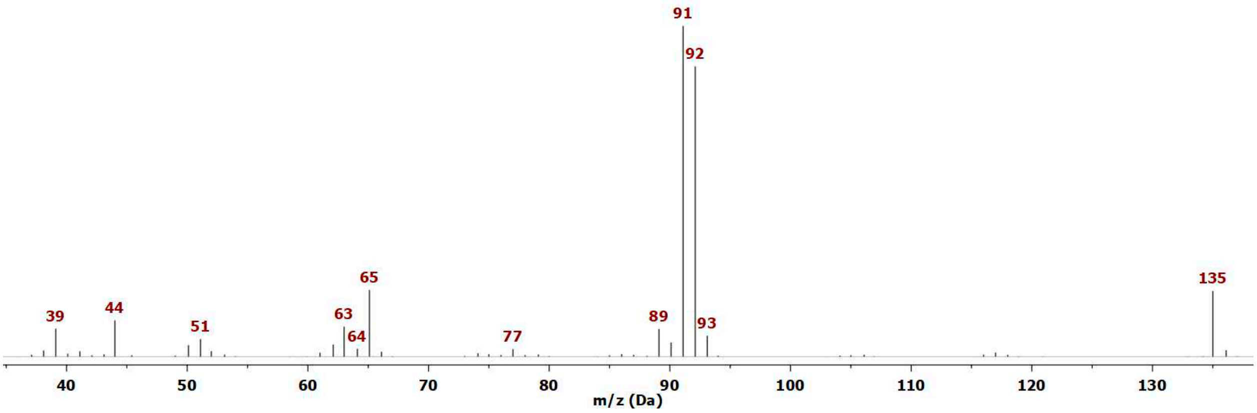

Figure S33. MS spectrum of 2-phenylacetamide (**9**)

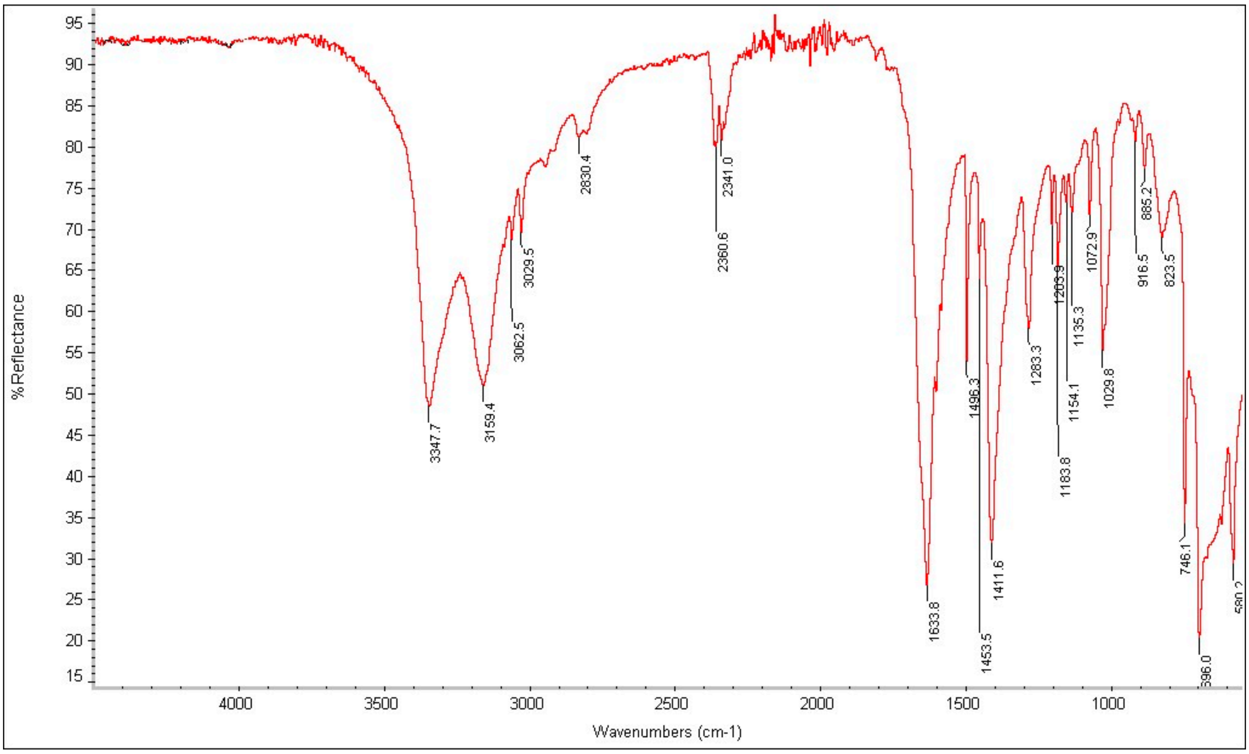

Figure S34. IR spectrum of 2-phenylacetamide (**9**)

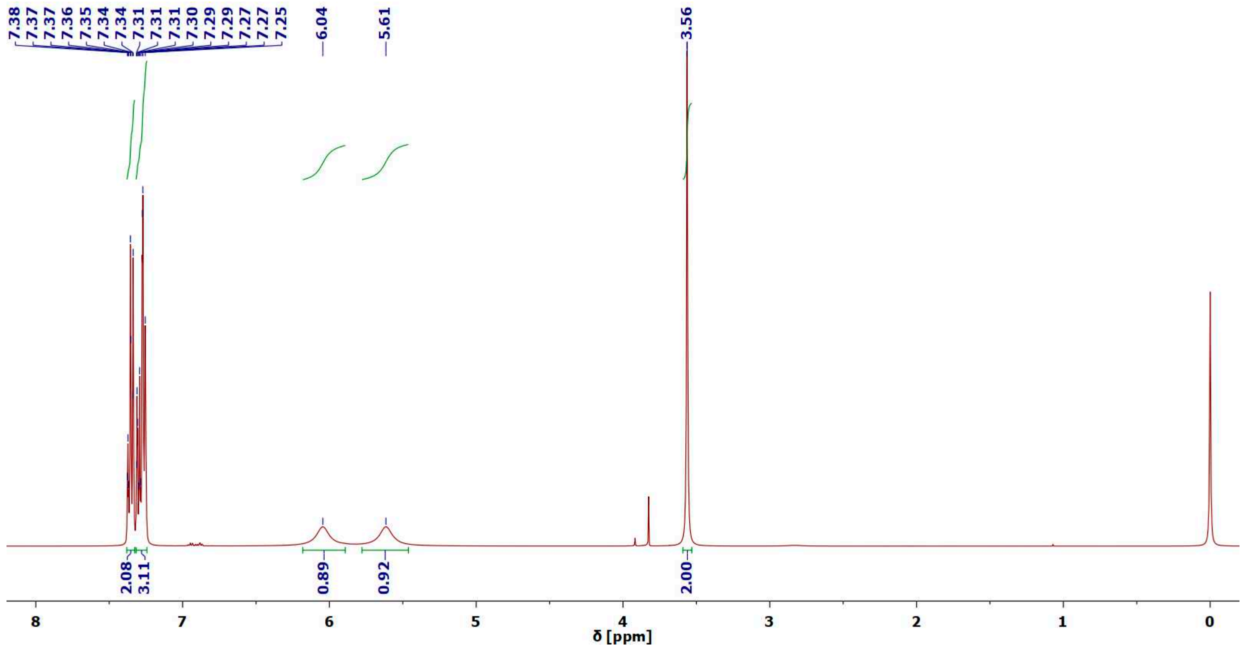

Figure S35. ^{1}H NMR spectrum (400 MHz, $CDCl_3$) of 2-phenylacetamide (**9**)

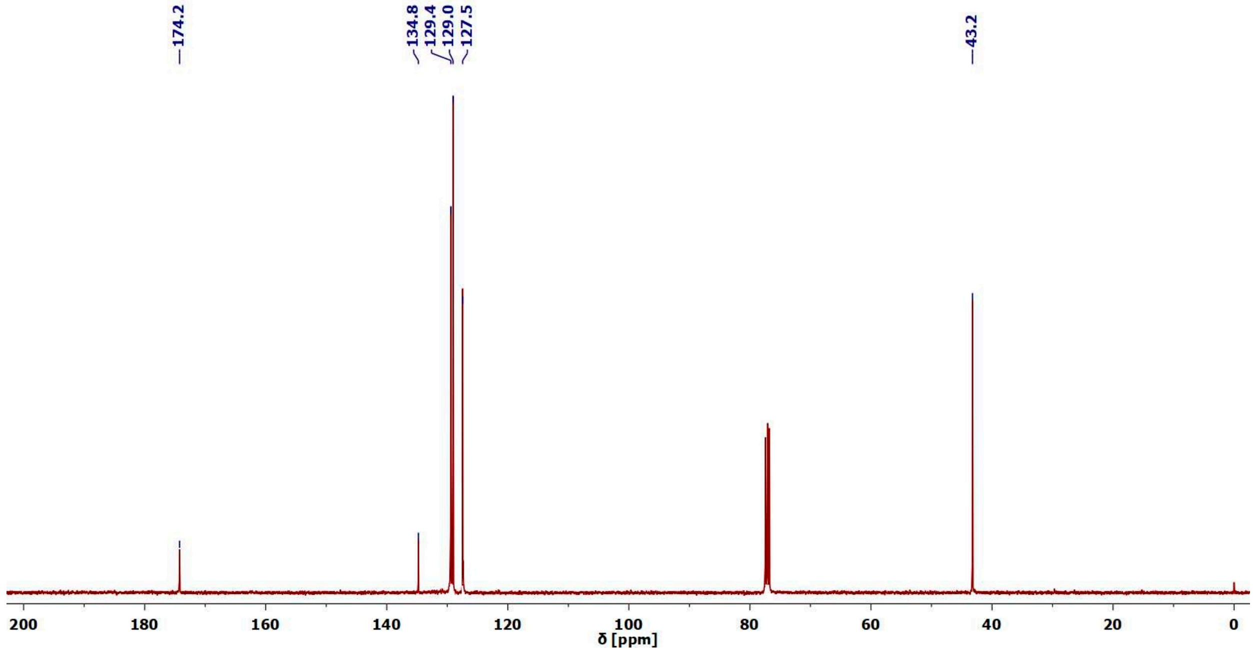

Figure S36. ^{13}C NMR spectrum (100.6 MHz, $CDCl_3$) of 2-phenylacetamide (**9**)

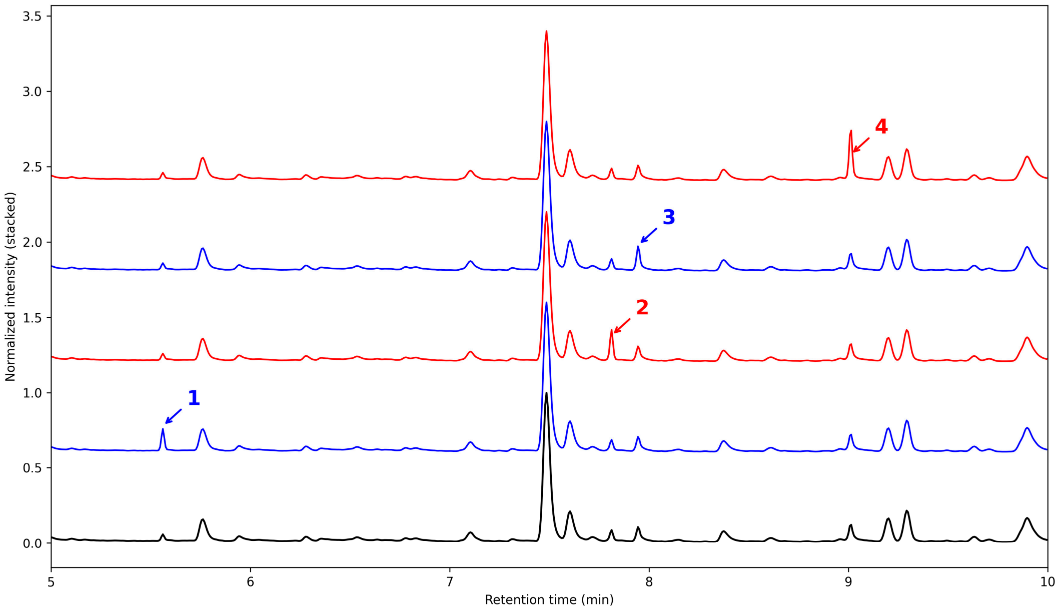

Figure S37. Gas co-chromatography of synthetic amides **1-4** with the whole-culture ethyl acetate extract of *Streptomyces* sp. NP10

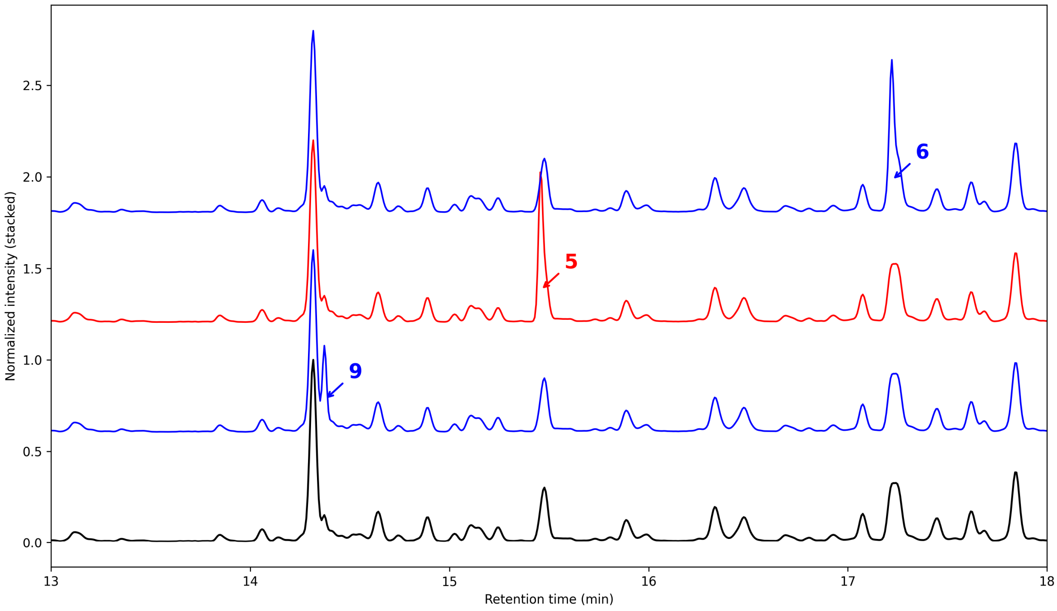

Figure S38. Gas co-chromatography of synthetic amides **5**, **6** and **9** with the whole-culture ethyl acetate extract of *Streptomyces* sp. NP10

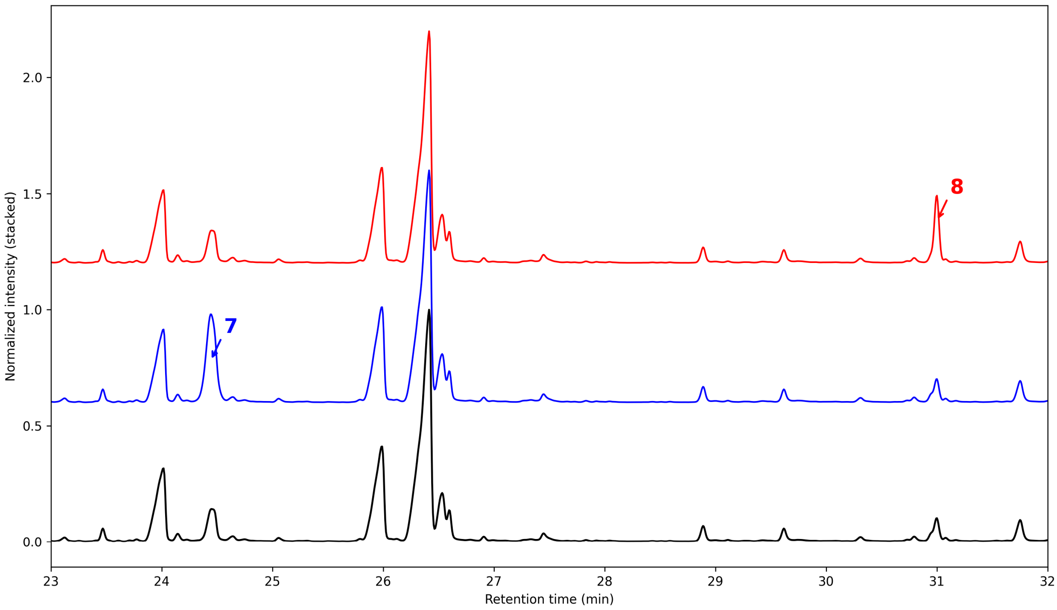

Figure S39. Gas co-chromatography of synthetic amides **7** and **8** with the whole-culture ethyl acetate extract of *Streptomyces* sp. NP10

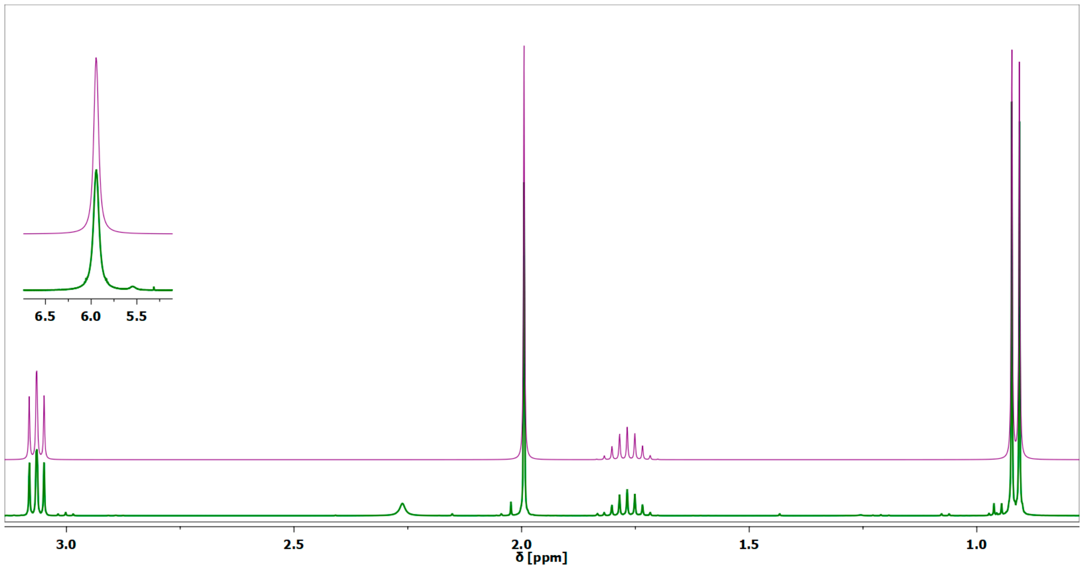

Figure S40. A comparison of the experimental (green) and simulated (violet) ^{1}H NMR spectra of *N*-(2-methylpropyl)acetamide (**1**)

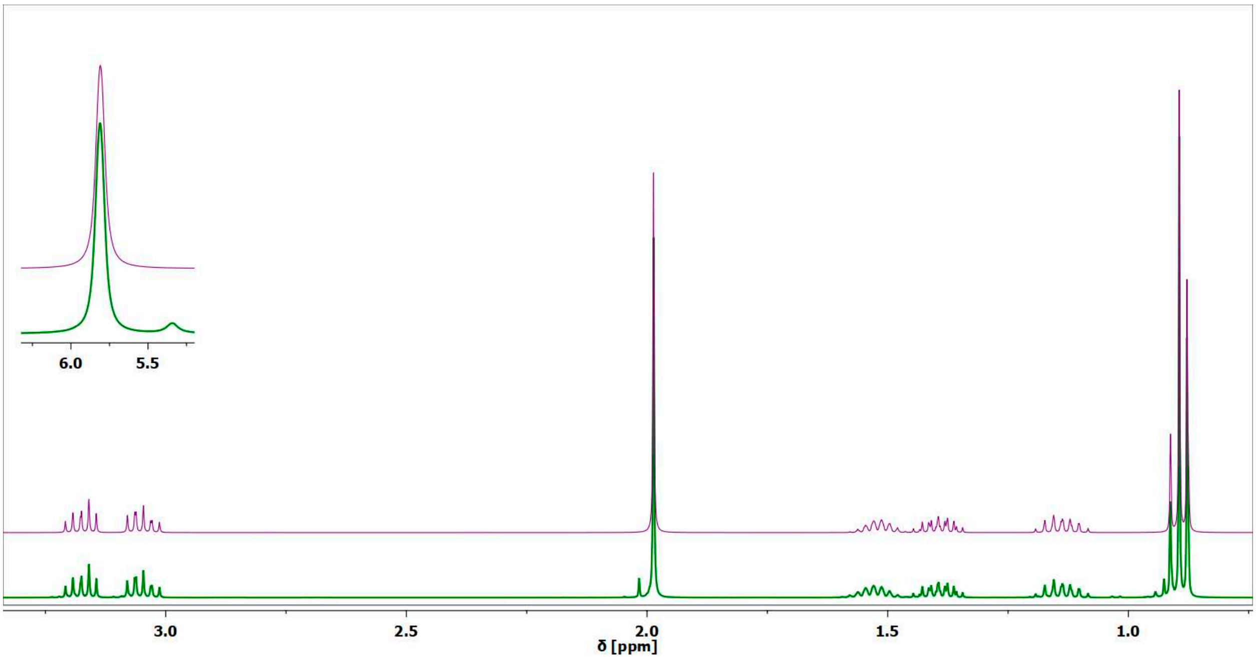

Figure S41. A comparison of the experimental (green) and simulated (violet) ^{1}H NMR spectra of *N*-(2-methylbutyl)acetamide (**2**)

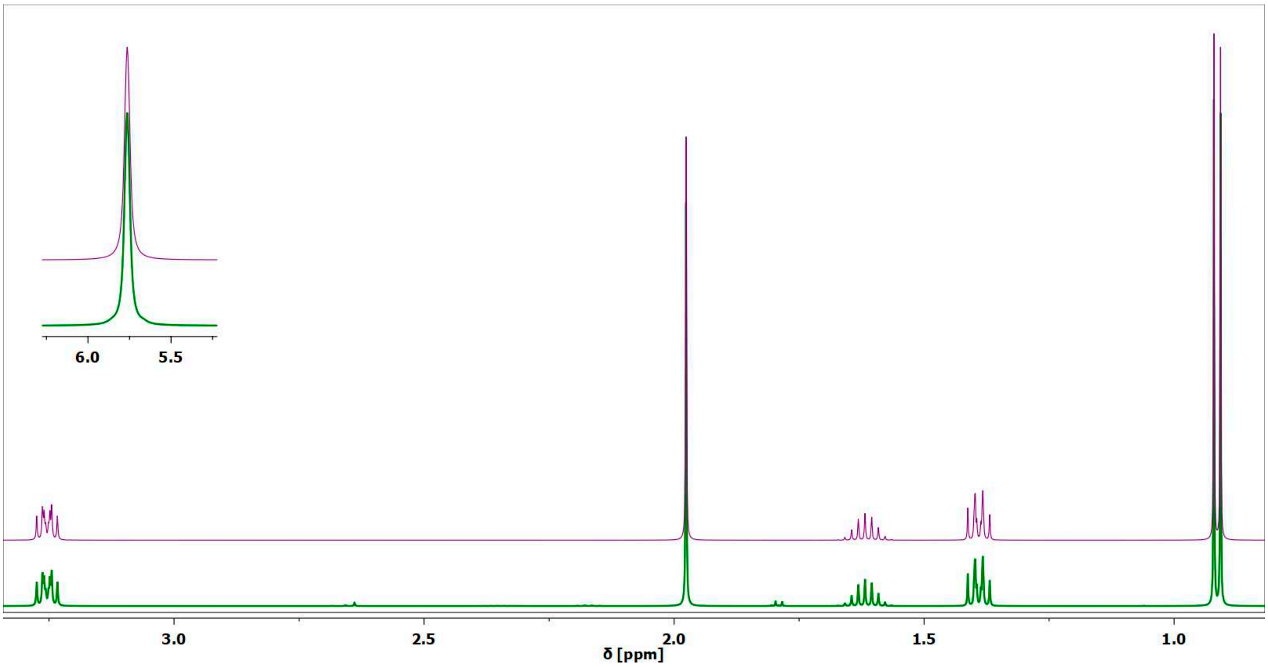

Figure S42. A comparison of the experimental (green) and simulated (violet) ^{1}H NMR spectra of *N*-(3-methylbutyl)acetamide (**3**)

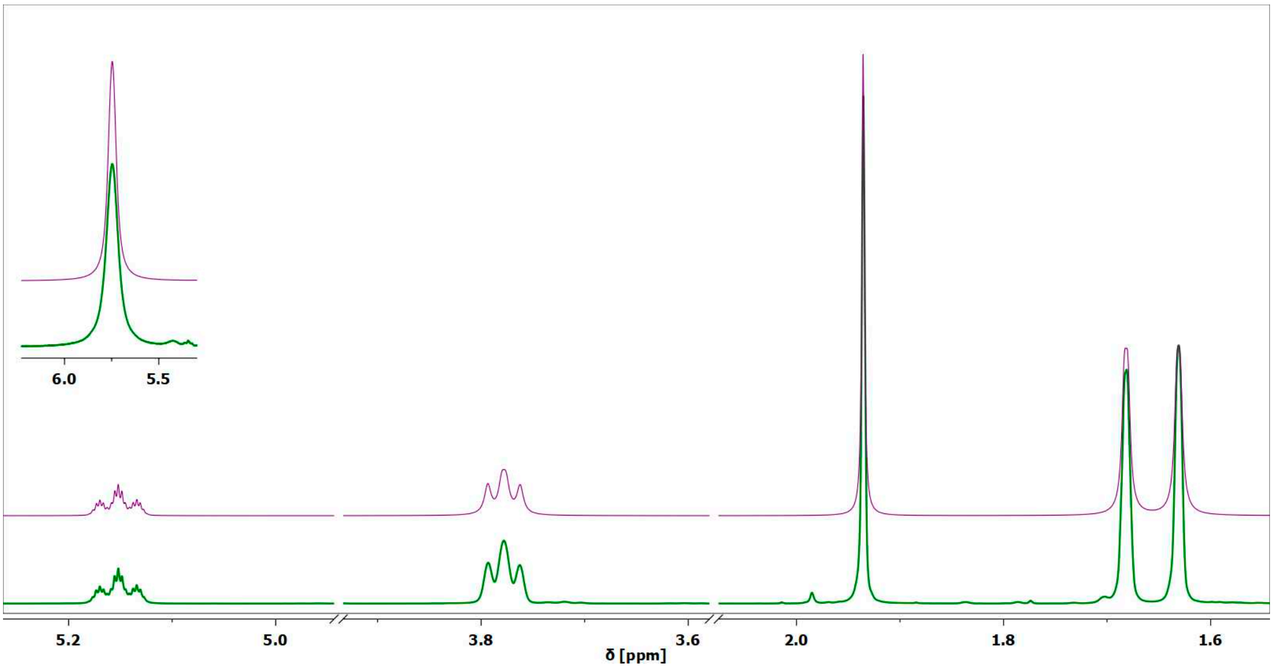

Figure S43. A comparison of the experimental (green) and simulated (violet) ^{1}H NMR spectra of *N*-(3-methyl-2-butenyl)acetamide (**4**)

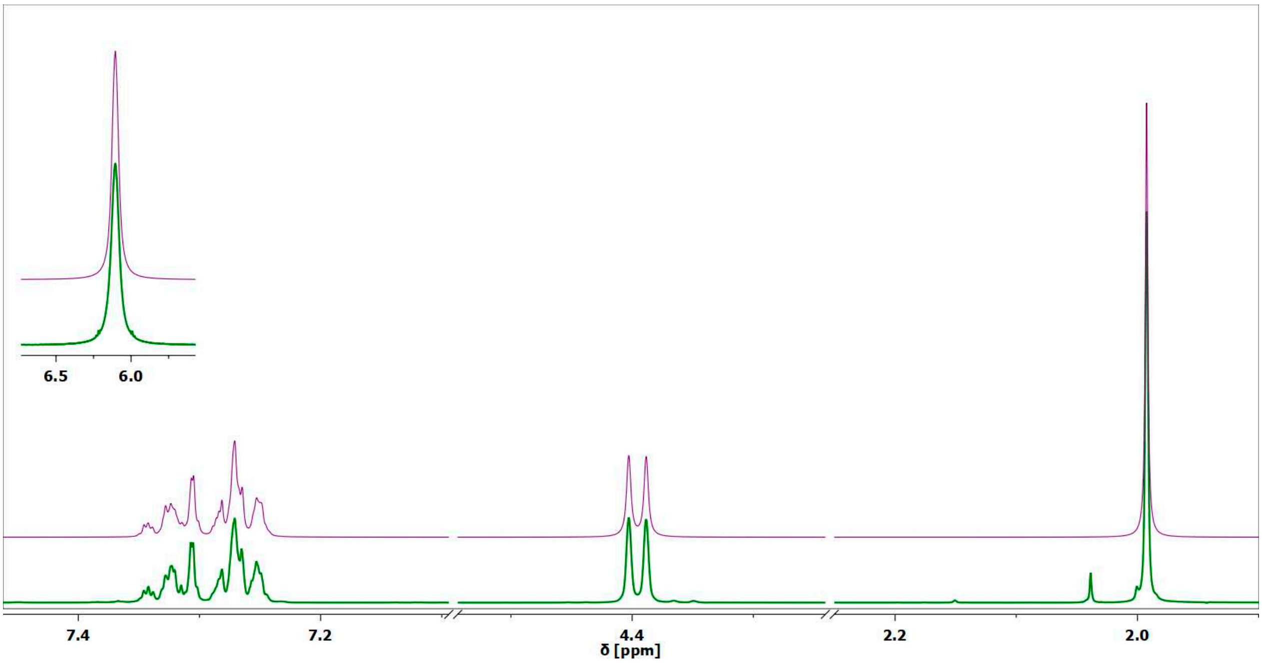

Figure S44. A comparison of the experimental (green) and simulated (violet) ^{1}H NMR spectra of *N*-benzylacetamide (**5**)

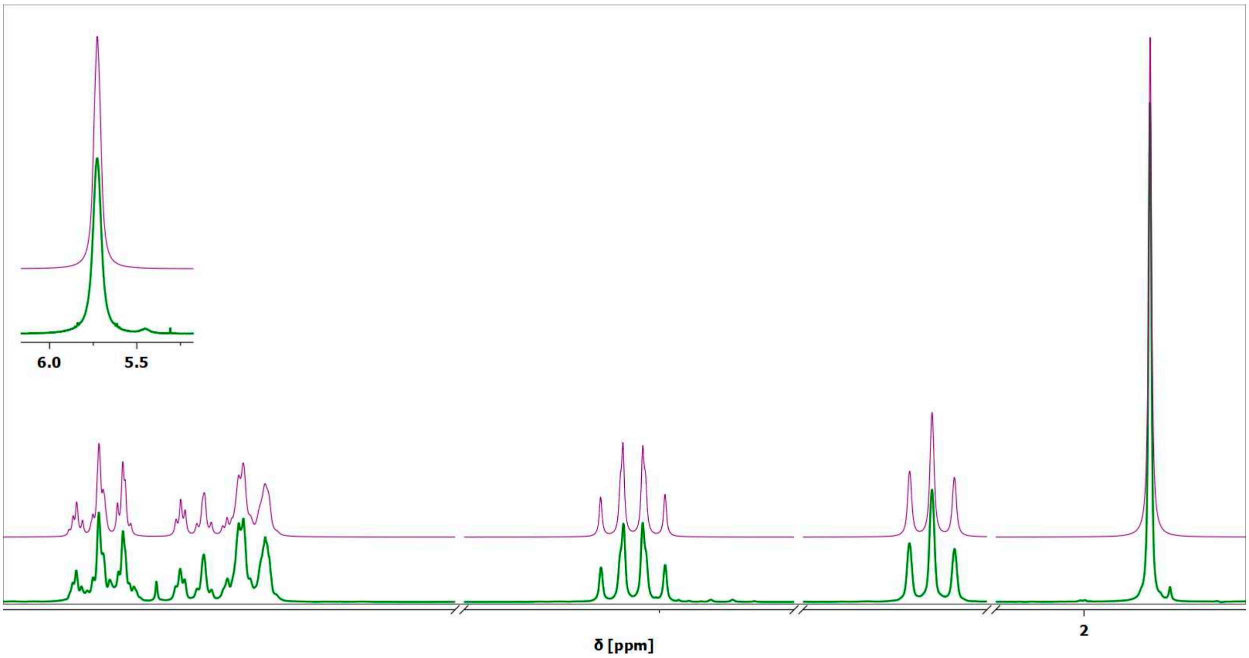

Figure S45. A comparison of the experimental (green) and simulated (violet) ^{1}H NMR spectra of *N*-(2-phenylethyl)acetamide (**6**)

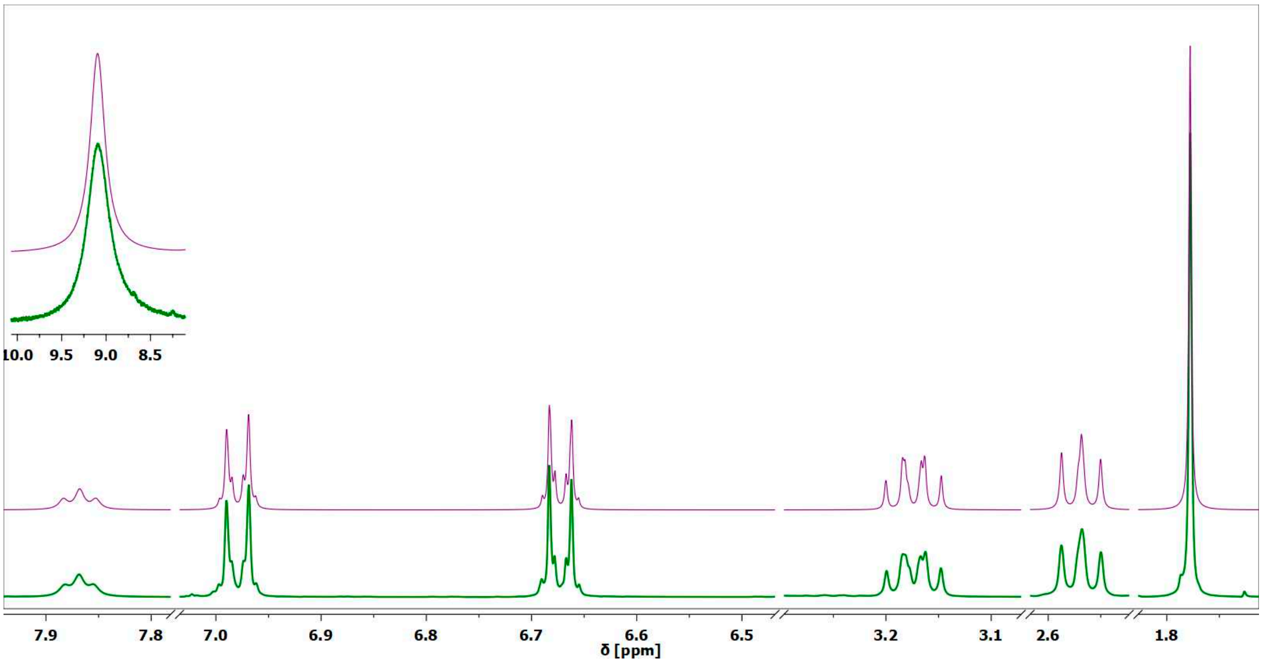

Figure S46. A comparison of the experimental (green) and simulated (violet) ^{1}H NMR spectra of *N*-acetyltyramine (**7**)

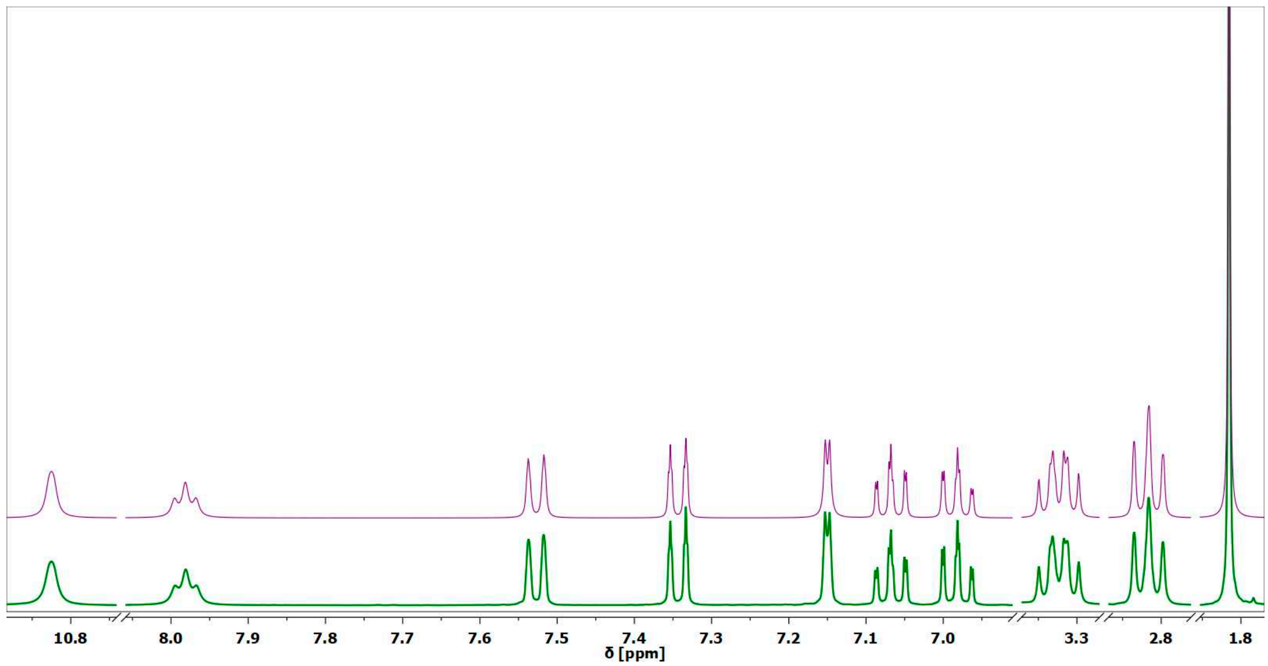

Figure S47. A comparison of the experimental (green) and simulated (violet) ^{1}H NMR spectra of *N*-acetyltryptamine (**8**)

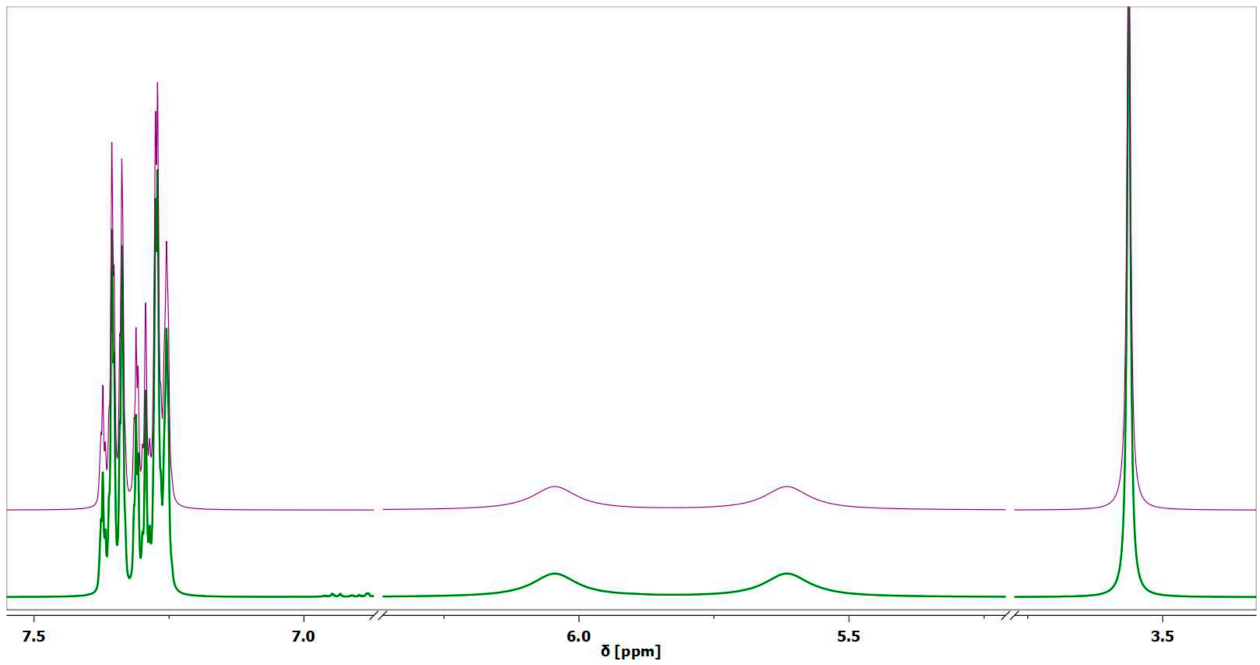

Figure S48. A comparison of the experimental (green) and simulated (violet) ^{1}H NMR spectra of 2-phenylacetamide (**9**)

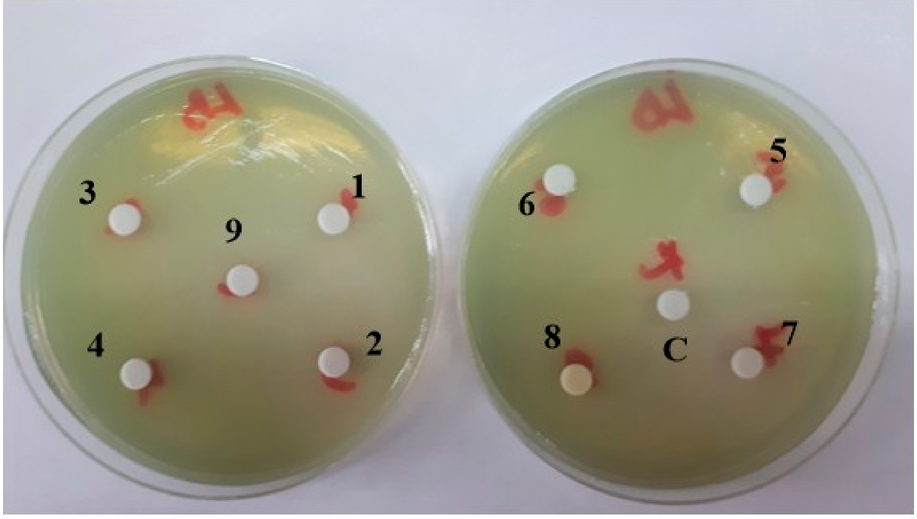

Figure S49. The effect of amides **1-9** on pyocyanin production in *Pseudomonas aeruginosa* PAO1

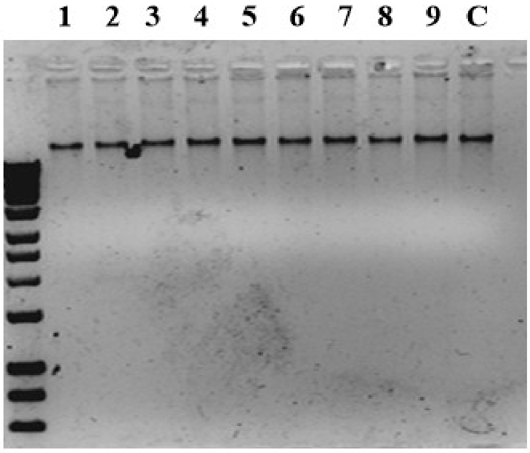

Figure S50. Evaluation of DNA binding by amides **1-9** using gel electrophoresis

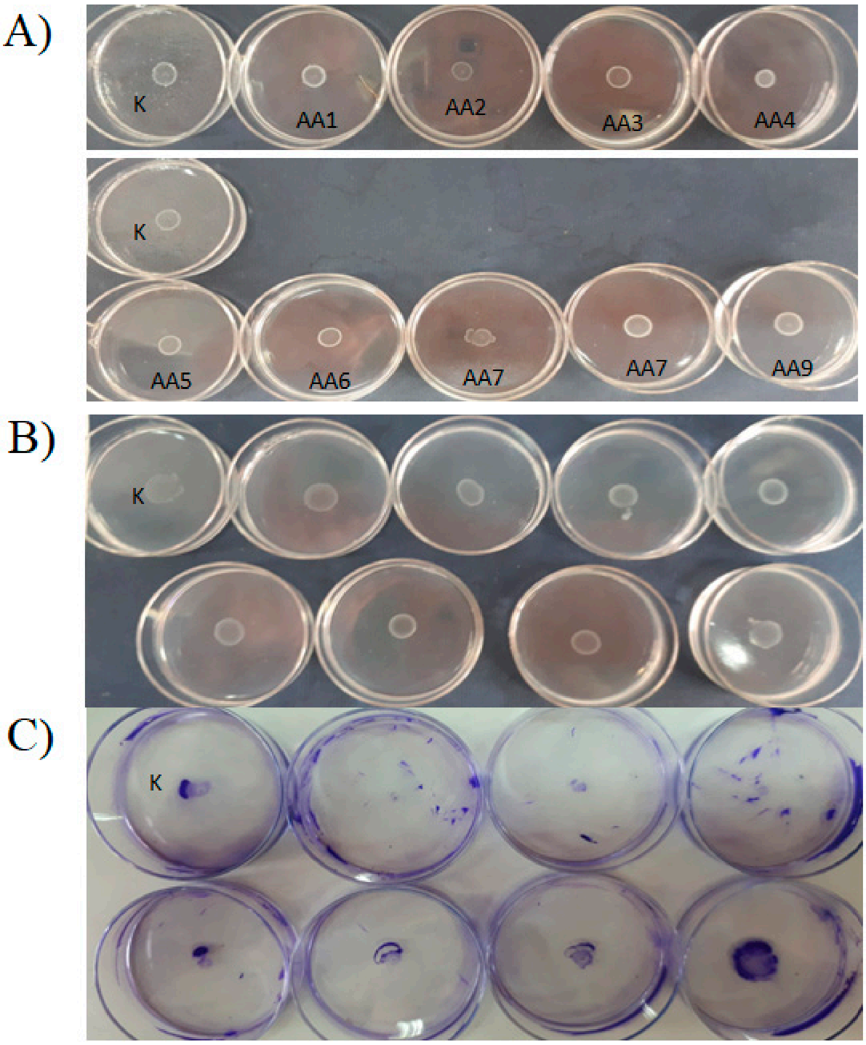

Figure S51. The effects of amides **1-9** on the swimming (A), swarming (B), and twitching (C) motilities of *Pseudomonas aeruginosa* PAO1

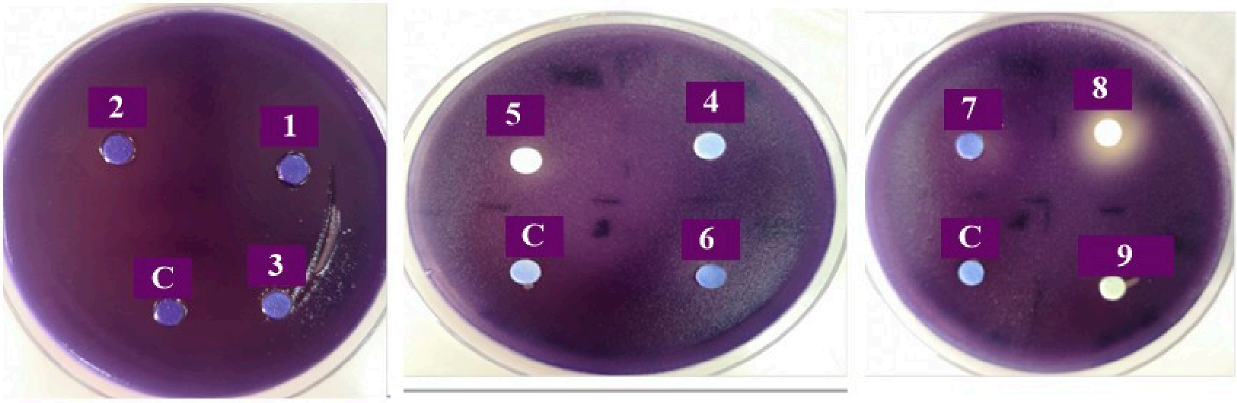

Figure S52. The effect of amides **1-9** on violacein production in *Chromobacterium violaceum* CV026

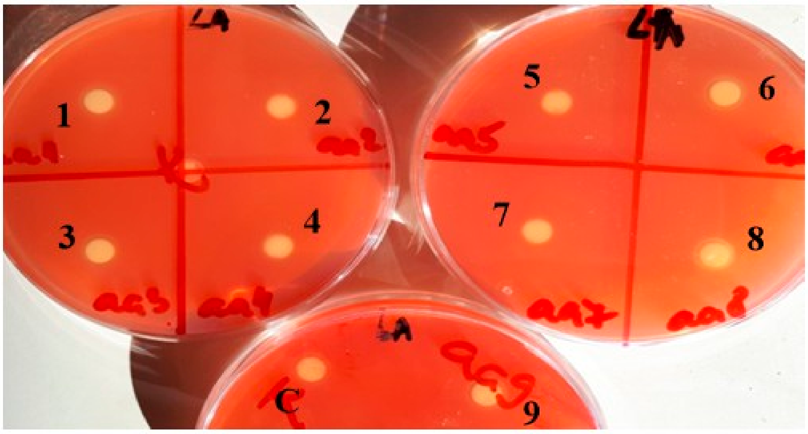

Figure S53. The effect of amides **1-9** on prodigiosin biosynthesis in *Serratia marcescens* ATCC 27117

Table S1 Values of the one-bond coupling constant between the NH proton and ^{15}N [$^{1}J(^{15}$N–H)] and the three-bond coupling constant between ^{15}N and the geminal methylene protons [$^{3}J(^{15}$N–H)] measured for acetamides **1-3** and **4-8**

**Compound**	**$^{1}J(^{15}$N–H) in Hz**	**$^{3}J(^{15}$N–H) in Hz**
**1**	89.8	6.0
**2**	89.7	6.0
**3**	89.6	5.6
**5**	90.7	5.7
**6**	90.1	5.9
**7**	91.3	6.4
**8**	91.5	5.7

Table S2 Overview of amide (**1-9**) concentrations applied in bioassays

**Amide**	**Molecular weight [g/mol]**	**Cytotoxicity assay**		**Antimicrobial disk diffusion assay**		**Antimicrobial microdilution method**		**Static biofilm formation assay**		**Autoinducers production assays**		**Motility assays**		**DNA interaction assay**		**Pyocyanin assay**		**Violacein and prodigiosin assays**	
		**µM**	**µg/mL**	**µg/disk**	**µmol/disk**	**µg/mL**	**µM**	**µg/mL**	**µM**	**µg/mL**	**µM**	**µg/mL**	**µM**	**µM**	**µg/mL**	**µg/disk**	**µmol/disk**	**µg/mL**	**µM**
**1**	115	100	11.5	250	2.2	500	4.3×10^3	250	2.2×10^3	250	2.2×10^3	250	2.2×10^3	400	46	250	2.2	250	2.2×10^3
**2**	129	100	12.9	250	1.9	500	3.9×10^3	250	1.9×10^3	250	1.9×10^3	250	1.9×10^3	400	51.6	250	1.9	250	1.9×10^3
**3**	129	100	12.9	250	1.9	500	3.9×10^3	250	1.9×10^3	250	1.9×10^3	250	1.9×10^3	400	51.6	250	1.9	250	1.9×10^3
**4**	127	100	12.7	250	2.0	500	3.9×10^3	250	2.0×10^3	250	2.0×10^3	250	2.0×10^3	400	50.8	250	2.0	250	2.0×10^3
**5**	149	100	14.9	250	1.7	500	3.4×10^3	250	1.7×10^3	250	1.7×10^3	250	1.7×10^3	400	59.6	250	1.7	250	1.7×10^3
**6**	163	100	16.3	250	1.5	500	3.1×10^3	250	1.5×10^3	250	1.5×10^3	250	1.5×10^3	400	65.2	250	1.5	250	1.5×10^3
**7**	179	100	17.9	250	1.4	500	2.8×10^3	250	1.4×10^3	250	1.4×10^3	250	1.4×10^3	400	71.6	250	1.4	250	1.4×10^3
**8**	202	100	20.2	250	1.2	500	2.5×10^3	250	1.2×10^3	250	1.2×10^3	250	1.2×10^3	400	80.8	250	1.2	250	1.2×10^3
**9**	135	100	13.5	250	1.9	500	3.7×10^3	250	1.9×10^3	250	1.9×10^3	250	1.9×10^3	400	54	250	1.9	250	1.9×10^3

^{1}H NMR Full Spin Analysis of Amides **1-9**

Although the NH signal in acetamides **1**-**6** appeared as a very broad singlet, decoupling experiments unambiguously revealed scalar coupling with vicinal methylene protons. The corresponding ^{3}J coupling constants were determined through spectral simulation and found to range from 5.40 to 6.15 Hz. In the case of *N*-acetyltyramine (**7**) and *N*-acetyltryptamine (**8**), NMR spectra were recorded in DMSO-d_6, a highly polar solvent capable of strong interactions with NH protons. These interactions disrupt intramolecular hydrogen bonding, thereby allowing the multiplicity of the NH signal to be more clearly observed [1]. Under these conditions, the NH resonance appeared as a broad triplet at 7.87 ppm for amide **7** and at 7.98 ppm for amide **8**, with corresponding ^{3}J coupling constants of 5.60 and 6.20 Hz, respectively.

The broadening of the NH signal can be attributed to residual scalar coupling with the quadrupolar ^{14}N nucleus, as well as the presence of ^{15}N satellite signals. Through analysis of the proton NMR spectra, both the one-bond coupling constant between the NH proton and ^{15}N [$^{1}J(^{15}N–H)$] and the three-bond coupling constant between ^{15}N and the geminal methylene protons [$^{3}J(^{15}N–H)$] were determined for acetamides**1**-**3** and **5**-**8**. The measured $^{1}J(^{15}N–H)$ values ranged from 89.6 to 91.5 Hz, while $^{3}J(^{15}N–H)$values were found to be between 5.6 and 6.4 Hz (Table S1).

As far as we are aware, scalar coupling between the NH proton and geminal methylene protons has been noted in only a few studies involving acetamides **4** and **8**. In the former case, concerning amide **4** [2], the coupling constant was not determined, whereas in the latter, for amide **8**, values of 6.0 [3] and 6.2 Hz [4], were reported. In contrast, to date, ^{15}N–H coupling constants in these systems have not been characterized.

Due to the presence of a chiral center, *N*-(2-methylbutyl)acetamide (**2**) exhibits two diastereotopic methylene groups. The methylene protons adjacent to the amide functionality appeared as a doublet of doublet of doublets (ddd) and were observed at higher frequency (δ ~3.0-3.2 ppm). In contrast, the second -CH_2- group appeared as doublet of doublet of quartets (ddq) at δ ~1.1-1.4 ppm. Notably, the coupling constants with the methine proton differed significantly, with values of 5.15 and 8.00 Hz, respectively. Despite the close resonance and apparent overlap of the two methyl groups originating from the amine moiety, spectral simulation enabled their clear resolution. To the best of our knowledge, although^{1}H NMR chemical shifts for compound **2** have been previously reported in the literature, the corresponding signal multiplicitiesremain unresolved and have not been fully characterized [5–8].

In acetamides **3**, **7**, and **8**, the ethylene (–CH_2–CH_2–) moieties give rise to an AA'XX' spin system, observed as complex multiplet patterns in the ^{1}H NMR spectra. Although the two protons on each methylene group are chemically equivalent, they are magnetically non-equivalent due to restricted rotation around the C–N bond, resulting in distinct spin–spin splitting. This magnetic non-equivalence primarily arises from significant geminal (^{2}J) coupling between the protons within each methylene group, with observed coupling constants ranging from –13.00 to –13.70 Hz. These findings provide new insights into the spin system complexity and magnetic behavior of ethylene fragments in such acetamide structures - an aspect that has largely been overlooked in previous studies.

In previous NMR studies of acetamides **3**, **7**, and **8**, the multiplicities of the methylene signals were either unresolved, only partially resolved, or incorrectly assigned. For instance, in four related studies on Queensland

fruit fly volatiles, the signals corresponding to the two methylene groups in *N*-(3-methylbutyl)acetamide (**3**) were generally described as unresolved multiplets [5–7,9]. In another study, the more downfielded methylene signal was assigned as a multiplet, while the other was reported as a quartet with a coupling constant of 7.1 Hz [10].

N-Acetyltyramine (**7**) and *N*-acetyltryptamine (**8**) have been extensively studied by NMR spectroscopy under various solvent conditions and at different magnetic field strengths. Nevertheless, the signals corresponding to their ethylene moieties have frequently been described as unresolved multiplets [11,12] or interpreted using a first-order approximation. In cases where coupling with the NH proton was observed, the patterns were variably reported as a quartet and a triplet [4], or as a doublet of triplets and a triplet [3]. This limited resolution of the methylene signals in earlier studies is likely due to the use of less polar solvents, such as $CDCl_3$, and/or NMR instruments operating at lower magnetic field strengths, both of which can reduce spectral resolution and obscure subtle spin-spin coupling patterns.Moreover, geminal coupling was often neglected in spectral interpretation, with the methylene signals frequently assigned as two simple triplets [13,14], thereby oversimplifying the actual spin system.

Interestingly, such second-order splitting patterns are not observed in acetamide **6**. The methylene group adjacent to the amide nitrogen, which appears more downfield, shows a doublet of triplets, while the methylene group next to the aromatic ring appears as a broad triplet, more precisely, a triplet of triplets, due to additional long-range benzylic coupling (^4J = –0.4 Hz). However, another second-order AA’BB’C spin system corresponding to the phenyl group was observed in the ^{1}H NMR spectrum of *N*-(2-phenylethyl)acetamide (**6**). The coupling constants measured were consistent with those reported for several phenethyl esters [15]. Similar complex phenyl group signals were also detected in the ^{1}H NMR spectra of compounds **5** and **9**, and these patterns were successfully resolved through spectral simulation.

In addition to the previously described AA’XX’ spin system originating from the ethylene moiety in *N*-acetyltyramine (**7**), a similar second-order coupling pattern was observed for the *para*-substituted benzene ring. In earlier studies, only the vicinal ($^3\boldsymbol{J}$) coupling between *ortho*-protons have been reported [14,16]. Likewise, in the case of *N*-acetyltryptamine (**8**), only ^3J coupling constants for protons within the indole ring have been previously determined [3,4]. In the present study, spectral simulations enabled the resolution and assignment of long-range aromatic couplings, including four- and five-bond interactions (^4J and ^5J), as well as homoallylic coupling between the indole NH and the methylene group in compound **8**. These findings provide a more comprehensive understanding of the aromatic spin systems in these acetamides.

N-(3-Methyl-2-butenyl)acetamide (**4**) has previously been reported only once, as a synthetic intermediate in the preparation of a structurally related natural product, where the double bond was subsequently dihydroxylated and the compound spectrally characterized [2]. In that study, the ^{1}H NMR spectrum was recorded at 100 MHz, resulting in limited resolution: the CH, CH_2, and CH_3 signals were reported simply as a triplet, triplet, and two singlets, respectively. In contrast, at the higher magnetic field strength used in our study, allylic couplings were clearly resolved, giving rise to more complex splitting patterns - namely, a doublet of doublets, a triplet of quartets of quartets, and two doublets - reflecting the true coupling interactions within the molecule.

References

1. Aksić, J., Genčić, M., Stojanović, N., Radulović, N., Zlatković, D., Dimitrijević, M., Stojanović-Radić, Z., Srbljanović, J., Štajner, T., Jovanović, L. New iron twist to chloroquine - Upgrading antimalarials with immunomodulatory and antimicrobial features. *J. Med. Chem.* **2023**, *66*, 2084–2101. DOI: 10.1021/acs.jmedchem.2c01851
2. Eichholzer, J.V., Lewis, I.A., Macleod, J.K., Oelrichs, P.B., Vallely, P.J. Galegine and a new dihydroxyalkylacetamide from *Verbesina enceloiodes*. *Phytochemistry* **1982**, *21*, 97–99. DOI: 10.1016/0031-9422(82)80021-6
3. Li, Y., Li, X.F., Kim, D.S., Choi, H.D., Son, B.W. Indolyl alkaloid derivatives, N_b-acetyltryptamine and oxaline from a marine-derived fungus. *Arch. Pharm. Res.* **2003**, *26*, 21–23. DOI: 10.1007/BF03179925
4. Astolfi, P., Panagiotaki, M., Rizzoli, C., Greci, L. Reactions of indoles with nitrogen dioxide and nitrous acid in an aprotic solvent. *Org. Biomol. Chem.* **2006**, *4*, 3282–3290. DOI: 10.1039/B607680G
5. Park, S.J., Pérez, J., Mendez, V., Taylor, P.W. Rectal glands and tergal glands as sources of volatile pheromones in cucumber fruit fly, *Zeugodacus cucumis*. *Sci. Rep.* **2025**, *15*, 743. DOI: 10.1038/s41598-024-84356-6
6. Noushini, S., Park, S.J., Jamie, I., Jamie, J., Taylor, P.W. Sampling technique biases in the analysis of fruit fly volatiles: A case study of Queensland fruit fly. *Sci. Rep.* **2020**, 19799. DOI: 10.1038/s41598-020-76622-0
7. Pérez, J., Park, S.J., Taylor, P.W. Domestication modifies the volatile emissions produced by male Queensland fruit flies during sexual advertisement. *Sci. Rep.* **2018**, *8*, 16503. DOI: 10.1038/s41598-018-34569-3
8. Yamazaki, H., Horikawa, H., Nishitani, T., Iwasaki, T. A facile synthesis of optically pure amines by reduction of *N*-acyl-α-methoxyalkylamines derived from α-amino acids using triethylsilane. *Chem. Pharm. Bull.* **1990**, *38*, 2024–2026. DOI: 10.1248/cpb.38.2024
9. Park, S.J., Pandey, G., Castro-Vargas, C., Oakeshott, J.G., Taylor, P.W., Mendez, V. Cuticular chemistry of the Queensland fruit fly *Bactroceratryoni* (Froggatt). *Molecules* **2020**, *25*, 4185. DOI: 10.3390/molecules25184185
10. Milan, M., Carboni, G., Salamone, M., Costas, M., Bietti, M. Tuning selectivity in aliphatic C–H bond oxidation of *N*-alkylamides and phthalimides catalyzed by manganese complexes. *ACS Catalysis* **2017**, *7*, 5903–5911. DOI: 10.1021/acscatal.7b02151
11. Häring, A.P., Biallas, P., Kirsch, S.F. An unconventional reaction of 2,2-diazido acylacetates with amines. *Eur. J. Org. Chem.* **2017**, *2017*, 1526–1539. DOI: 10.1002/ejoc.201601625
12. Li, C., Wang, M., Lu, X., Zhang, L., Jiang, J., Zhang, L. Reusable brønsted acidic ionic liquid efficiently catalyzed *N*-formylation and *N*-acylation of amines. *ACS Sustain. Chem. Eng.* **2020**, *8*, 4353–4361. DOI: 10.1021/acssuschemeng.9b06591
13. Schuck, D.C., Jordão, A.K., Nakabashi, M., Cunha, A.C., Ferreira, V.F., Garcia, C.R. Synthetic indole and melatonin derivatives exhibit antimalarial activity on the cell cycle of the human malaria parasite *Plasmodium falciparum*. *Eur. J. Med. Chem.* **2014**, *78*, 375–382. DOI: 10.1016/j.ejmech.2014.03.055
14. Gutiérrez, M., Capson, T.L., Guzmán, H.M., González, J., Ortega-Barría, E., Quiñoá, E., Riguera, R. Antiplasmodial metabolites isolated from the marine octocoral *Muricea austera*. *J. Nat. Prod.* **2006**, *69*, 1379–1383. DOI: 10.1021/np060007f
15. Genčić, M., Aksić, J., Mladenović, M., Stošić, M.Ž., Radulović, N. Phenethyl angelate – A new ester from immortale essential oil? *Facta Universitatis, Series: Physics, Chemistry and Technology*, **2021**, *19*, 17–29. DOI: 10.2298/FUPCT2101017G
16. Ivanova, V., Graefe, U., Schlegel, R., Schlegel, B., Gusterova, A., Kolarova, M., Aleksieva, K. Isolation and structure elucidation of tyramine and indole alkaloids from antarctic strain *Microbisporaaerata* IMBAS-11A. *Biotechnol. Biotechnol. Equip.* **2003**, *17*, 128–133. DOI: 10.1080/13102818.2003.10817070
